# Evaluating language models for mathematics through interactions

**DOI:** 10.1073/pnas.2318124121

**Published:** 2024-06-03

**Authors:** Katherine M. Collins, Albert Q. Jiang, Simon Frieder, Lionel Wong, Miri Zilka, Umang Bhatt, Thomas Lukasiewicz, Yuhuai Wu, Joshua B. Tenenbaum, William Hart, Timothy Gowers, Wenda Li, Adrian Weller, Mateja Jamnik

**Affiliations:** ^a^University of Cambridge, Cambridge CB2 1TN, United Kingdom; ^b^University of Oxford, Oxford OX1 4BH, United Kingdom; ^c^Massachusetts Institute of Technology, Cambridge, MA 02139; ^d^The Alan Turing Institute, London NW1 2DB, United Kingdom; ^e^New York University, New York, NY 10011; ^f^Vienna University of Technology, Vienna 1040, Austria; ^g^x.AI, New York, NY 10038; ^h^Collége de France, Paris 75001, France

**Keywords:** human–computer interaction, theorem proving, language models, AI

## Abstract

There is much excitement about the opportunity to harness the power of large language models (LLMs) when building problem-solving assistants. However, the standard methodology of evaluating LLMs relies on static pairs of inputs and outputs; this is insufficient for making an informed decision about which LLMs are best to use in an interactive setting, and how that varies by setting. Static assessment therefore limits how we understand language model capabilities. We introduce CheckMate, an adaptable prototype platform for humans to interact with and evaluate LLMs. We conduct a study with CheckMate to evaluate three language models (InstructGPT, ChatGPT, and GPT-4) as assistants in proving undergraduate-level mathematics, with a mixed cohort of participants from undergraduate students to professors of mathematics. We release the resulting interaction and rating dataset, MathConverse. By analyzing MathConverse, we derive a taxonomy of human query behaviors and uncover that despite a generally positive correlation, there are notable instances of divergence between correctness and perceived helpfulness in LLM generations, among other findings. Further, we garner a more granular understanding of GPT-4 mathematical problem-solving through a series of case studies, contributed by experienced mathematicians. We conclude with actionable takeaways for ML practitioners and mathematicians: models that communicate uncertainty, respond well to user corrections, and can provide a concise rationale for their recommendations, may constitute better assistants. Humans should inspect LLM output carefully given their current shortcomings and potential for surprising fallibility.

## Significance

Large language models (LLMs) are increasingly powerful, but their evaluation is often static, which does not consider LLMs’ performance when interacting with humans. We develop a platform to empower interactive evaluation, observe real mathematicians interacting with and evaluating LLMs in theorem proving to study how people solve problems with their assistance, and taxonomize the interactions to enable in-depth analyses. Our work systematically studies the interactive evaluation of LLMs in target settings and provides takeaways for people using and developing LLMs.

Foundation models (1)—in particular large language models (LLMs) (2–4)—are increasingly human-facing, permitting users to interact with and elicit natural language responses (5, 6). Such interactive systems admit a plethora of new possibilities for human-machine collaboration (7–9). However, many existing evaluation approaches for LLMs are insufficient for use in informing how best to harness such models’ power in human-machine interaction. In particular, capability assessments for LLMs typically assume a noninteractive view: models are primarily evaluated statically with “ground truth” input–output pairs, and metrics are aggregated over a dataset (10), which may be misaligned with their use cases.

**Fig. 1.**
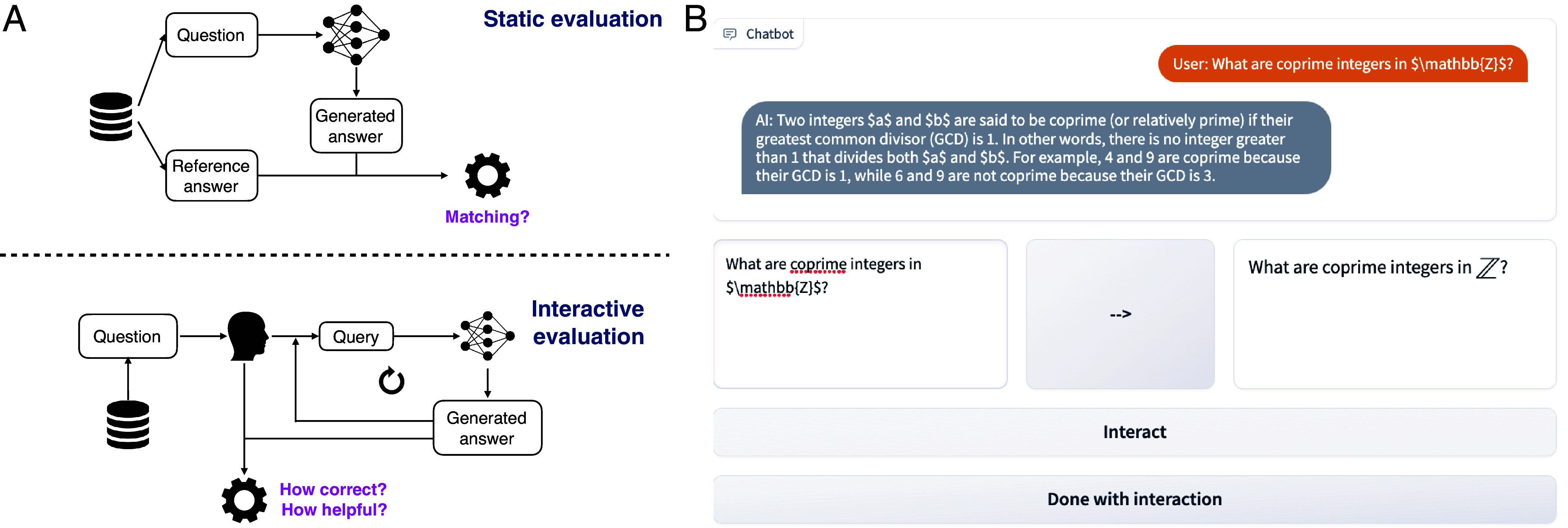
(*A*) Contrasting typical static evaluation (*Top*) with interactive evaluation (*Bottom*), wherein a human iteratively queries a model and rates the quality of responses. (*B*) Example subset of the chat interface from CheckMate where users interact with an LLM. The participant can type their query (*Lower Left*), which is compiled in LaTeX (*Lower Right*). When ready, the participant can press “Interact” and have their query routed to the model. The user can continue for multiple interactions. The entire chat history is presented for the user to refer to. When the user is done with their interaction, they can press “Done with interaction” to be proceed to rating the interaction. The participant also sees the problem text above the chat window; we include full screenshots in *SI Appendix*, Figs. S2 and S3.

To address this problem, we argue in this paper that *interactive* and *dynamic* evaluation of LLMs (11, 12) is essential for grasping their capabilities. We carry out such an interactive and dynamic evaluation of how humans use LLMs for assistance (see Fig. 1*A* for an overview of how interactive evaluation differs from conventional static evaluation) to better characterize their limitations, undesirable behaviors, and potential harms.

Evaluating LLM interactions is especially warranted in the case of informal mathematical theorem proving, wherein an agent is given a mathematical theorem and needs to propose a proof that is acceptable to the mathematical community. Informal theorem proving is special in that there is a formal notion of correctness at its core, yet most statements are expressed in natural language (informally). Important quality measures for the task include helpfulness and correctness, neither of which can be satisfactorily captured by automatic metrics (e.g., BLEU and ROUGE scores) (13–15). Letting humans communicate and evaluate these systems is crucial for their assessment.

Further, mathematics is an interactive practice. Recent works (16, 17) have shown that LLMs can benefit from feedback on their own language output (i.e., “generations”) in mathematical tasks, and this benefit can only be seen in multiturn interactive evaluations. Hence, we choose mathematics as a domain to highlight the value of human interactive evaluations; however, CheckMate and our broader evaluation methodology can be extended to other domains and chatbot-based systems (*SI Appendix*).

Concretely, we apply two methods to analyze human–LLM mathematical reasoning interactions: 1) structured evaluation, that is, rating every LLM generation in a conversation; and 2) free-form evaluation, where three domain experts (two leading mathematicians, and one computer scientist with expertise in formal mathematics) conduct instance-level case studies. The latter approach is inspired by the burgeoning literature of involving domain experts alongside ML practitioners in understanding model behavior (18, 19), and directly responds to the call from Burnell et al. (10) to engage with non-ML scholars to better understand LLM systems. Our study is interdisciplinary at its core.

Despite the large number of LLM-based chatbots, there is a paucity of open and unified platforms for eliciting fine-grained evaluations of interactions with users at scale. Hence, we develop a lightweight prototype interactive evaluation platform that is highly adaptable, called CheckMate.^*^ We leverage CheckMate to conduct an empirical study on undergraduate-level theorem proving (see an example problem in *SI Appendix*, *An Example Survey Problem*), over a suite of popular language models: InstructGPT (20), ChatGPT (5),^†^ and GPT-4 (21). We release the resulting interactions and evaluations on 261 human–model interactions in a dataset called MathConverse, from which we derive a preliminary taxonomy of user behaviors. We do not claim completeness for our taxonomy, because of the limited size of MathConverse. Our study is particularly compelling as it not only engages a group of participants with a wide range of mathematical experience, but engages with problems at a level of difficulty is higher than what is typically explored (22–24). We emphasize that CheckMate can be conveniently extended to domains other than mathematics. We also invite our domain experts (two leading mathematicians, and one computer scientist specializing in formal mathematics) to contribute in-depth interaction case studies to help better characterize current LLM mathematical reasoning capabilities. Throughout, we emphasize that we are not trying to draw broad conclusions across the entire LLM landscape. Rather, we aim to highlight the feasibility and value of incorporating interactions into the evaluation process, particularly when involving domain experts, and to elucidate potential human and model behavior patterns specifically in mathematics.

**Our three key contributions are as follows:**

- We introduce an adaptable platform, CheckMate, for evaluating language models by their interactions with human users. We demonstrate that scalable and valuable dynamic interactive evaluations are feasible by applying CheckMate to evaluate three language models on mathematical theorem proving.
- With interactions and evaluations collected from CheckMate via a mixed cohort study, we derive a taxonomy of user behaviors which identify crucial expected abilities of LLM-based mathematical assistants. We release the dataset of CheckMate interactions and evaluations, MathConverse.^‡^
- Through case studies conducted by domain experts, we add empirical evidence for several weaknesses of the LLMs that we explore, including algebraic manipulations, oververbosity, and overreliance on memorized solutions. We urge ML practitioners to help develop solutions to these challenges (such as better communication of uncertainty and ability to update user corrections) and to suggest good practices for LLM users (e.g., to heed caution when inspecting generations, as mistakes can be subtle). We encourage further interactive evaluation with LLMs, in mathematics and beyond, to inform how, when, and whether to deploy these models in assistive settings.

## Results

**Fig. 2.**
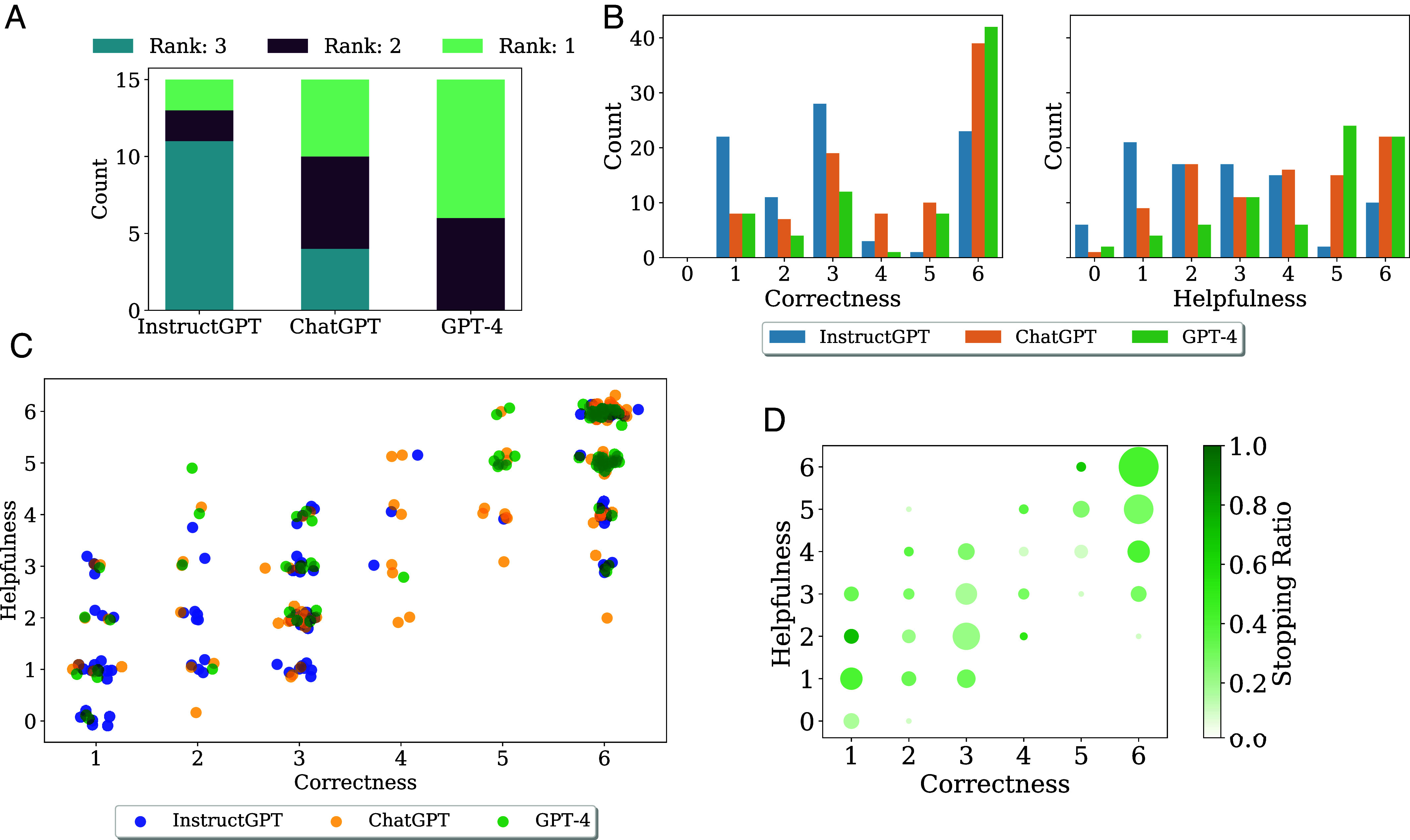
(*A*) Counts of model preference ratings postinteractions; participants ranked models based on preference as a mathematical assistant (lower rank is better). Ties were allowed and are included: Participants were permitted to assign the same rank to multiple models (*SI Appendix*, Additional Survey Observations). (*B*) Mathematical correctness and perceived helpfulness scores (all scores are an integer ∈{0,1,...,6}; higher is better) received for each model. Full details about the text associated with the scales of each score are included in *SI Appendix*, Additional Survey Details. (*C*) Comparing participants’ scores of the mathematical correctness against perceived helpfulness of each models’ generations. Each dot is a score for a single human–model interaction. We add slight jitter for visual ease given that points overlap. Interestingly, we observe cases where the perceived helpfulness and correctness of a generation diverge; that is, particular instances can be deemed incorrect yet somewhat helpful, or correct, but somewhat unhelpful. (*D*) The relationship between correctness and helpfulness scores and whether the step is terminal (i.e., the step after which the participant stopped interacting for a particular problem). The size of the bubbles indicates the number of that particular score pair (correctness, helpfulness). For a fixed score pair, the opacity indicates the ratio of stopping steps, that is, the number of terminal steps divided by the number of total steps.

We first present results from both of our evaluation methods—structured multistep interactive ratings and the free-form instance-based evaluation (Fig. 2)—before synthesizing key insights across the studies.

### Observations from Applying CheckMate.

We highlight our primary findings from applying CheckMate in the domain of mathematics, particularly assistance in undergraduate-level theorem-proving, through which we collect and introduce the MathConverse dataset (see example interface screen in Fig. 1 and *SI Appendix*).

#### Systems optimized for chat are preferred.

Participants were not informed of which model they were interacting with. Nonetheless, we nicely observe in Fig. 2*A* that, as expected, models optimized for chat (ChatGPT and GPT-4) are consistently rated as preferable to those not (InstructGPT), with GPT-4 being most frequently favored and much less often least preferable. That is, the lower bound (“worst case”) behavior of GPT-4 is consistently better than that of the other models (e.g., the model is never ranked as the worst assistant). We emphasize that these evaluations are not meant to be definitive assessments of model performance, but rather, highlight that interactive evaluation can yield a more nuanced understanding of model behavior beyond the common “snapshot” evaluation on standard benchmark datasets.

#### Perceived utility per model.

We next look at individual interactions. Participants were asked to rate the mathematical correctness and perceived helpfulness of each generation; we depict the helpfulness and correctness ratings across models in Fig. 2*B*. These data further reveal distinctions across models; notably, GPT-4 achieves consistently high helpfulness ratings, underscoring its potential perceived utility.

The generation in *SI Appendix*, *An Incorrect but Helpful Response*^§^ is rated to have “Multiple critical maths errors” (correctness score 2), while being “Somewhat useful” (helpfulness score 4). This response from the assistant is indeed plagued with errors and misunderstandings, but it also contains the structure of a correct proof as well as the Rank-Nullity Theorem, which is useful if used properly.

We observe in Fig. 2*C* that the perceived helpfulness and correctness correlate positively for all three models; although, interestingly, some generations can be deemed completely mathematically correct, but not particularly helpful (e.g., six correctness, three helpfulness). This can occur, for instance, for overly verbose responses (e.g., see the example in *SI Appendix*, *An Overly Verbose Response*).

These data further assert the value of multifactorial LLM evaluations, beyond a single scalar “goodness” score. We expand on these two points in our expert case studies.

### Taxonomizing User Behavior from MathConverse.

Additionally, we qualitatively study the *characteristics* of the participants’ interactions with the LLMs. A core motivation is to get a grasp on how mathematicians may actually *use* these systems in the wild—what queries they may initiate, and how they follow up with the model over the course of the interaction. Following Lee et al. (11), we offer a preliminary taxonomy of the kinds of queries found in our data. All interaction traces are released anonymously in our repository to support further study of human-machine interaction, along with our annotated taxonomy. Details on how we constructed and annotated such a taxonomy are included in *Materials and Methods* and in *SI Appendix*, *Additional Details on Taxonomy Construction*.

#### Initial interaction behavior.

We find that participants typically take one of four approaches in their first query to the AI assistant. Remarkably, over 90% of participants’ first interaction for each problem fall into one of the following interaction behavior types:

1. Seeking specific definitions of a concept mentioned in the problem (e.g., “Definition of Hall subgroup” or “What is the definition of ‘nullity’ in linear algebra?”).
2. Asking a general question about mathematics related to the problem (e.g., “When is a plane in $R^{3}$ parallel to another plane in $R^{3}$” or “In mathematics, what does it mean to Let $A\in K^{n\times n}$”).
3. Simply copy-pasting the entire problem statement, or a slight rephrasing of the original statement, optionally with prepended instructions (e.g., “Can you assist me in proving the following statement? [...]”).
4. Prompting the model for a single step of the problem, rather than the entire problem all at once (e.g., “We will first prove a lemma, let us call it Lemma 1 [...]”).

#### Mid-interaction behavior.

We observe a wider array of interaction modes after the first interaction. In addition to all of the behaviors above, such as users asking for more definitions, we find the following general patterns:

1. Asking a clarifying question (e.g., “Does it hold even when p is not a prime number?”).
2. Correcting the model output, occasionally with a clarifying question (e.g., “I understand. But your example is misleading. In your example, f has degree 2 and it has 2 roots, so it does not represent a valid counterexample. Can you show an example in which a polynomial has more roots than its degree?”).
3. Asking for clarification specifically about the generation from the model (e.g., what a particular symbol means—“What is τ here?”).
4. Asking why the model did something (e.g., “so why do you need to add the whole set at step 2?”).
5. Implicitly correcting the model (e.g., “That sounds like there being a homeomorphism. But a contraction is not a homeomorphism?”).
6. Asking for instances of a particular construction (e.g., “Can you exhibit an example to demonstrate that?”).

We also find that a few participants ask the model to “continue” if it stopped midway through, and some participants seemed to attempt popular prompt engineering tricks, for example, attempting to get the model to “restart” by telling it to forget what it had done before: “Forget what you’ve said before and try again. Start with $n^{x}+n^{y}=n^{z}$, divide both sides by $n^{z}$, and reason from there.” Further, we note that one participant asked for *intuition* about a concept: “What is the intuition behind the proof of this statement?” Here (full response in *SI Appendix*, *A Response about Intuition*), the model (GPT-4) provided a response rated as “definitely helpful,” indicative of a potential exciting assistive case going forward. In addition to revealing the kinds of interactions that mathematicians may make to help motivate the design of tools better equipped to handle such interactions (e.g., when participants ask for clarification), we see these observations as pointers to broader public education and “AI literacy” efforts as to what AI systems can be leveraged to help with and how to best query for this help (e.g., through particular prompt techniques).

#### Rating dynamics over the interaction trace.

As noted, we observe that several participants attempt to correct the model’s output or ask for clarification. Sometimes these occurrences would go on for a few successive trials; we refer to such correction-mistake interaction ruts as “frustration cycles.” We can see some of this behavior by inspecting the rating dynamics across interaction traces. In *SI Appendix*, Fig. S6, we see that in general, participants’ ratings begin to fall off over the course of interactions, and through Fig. 2*D*, we see that participants seem to stop when both ratings are higher than 4, indicating the model clearly can solve the problem and “assist” them), or with both ratings lower than 2 (indicative of the model completely failing to provide any further useful mathematical knowledge). We include participant testimonials about why they chose to stop in *SI Appendix*, *Post-Survey Testimonials from Participants*.

### Investigations into the MathConverse Annotated Taxonomy.

We build an annotated taxonomy by coding each user query; details are included in *Materials and Methods*, as well as in *SI Appendix*, *Additional Details on Taxonomy Construction*. The taxonomy enables us, and other researchers, to understand the kinds of queries users make. We ask a few questions here: 1) how do the queries made in the first interaction compare to those in the second; 2) is there a relationship between the kinds of queries made by participants who had extensive prior experience interacting with AI systems versus those who did not; and 3) is there a relationship between particular interaction types and the scores assigned to the models’ corresponding responses?

**Fig. 3.**
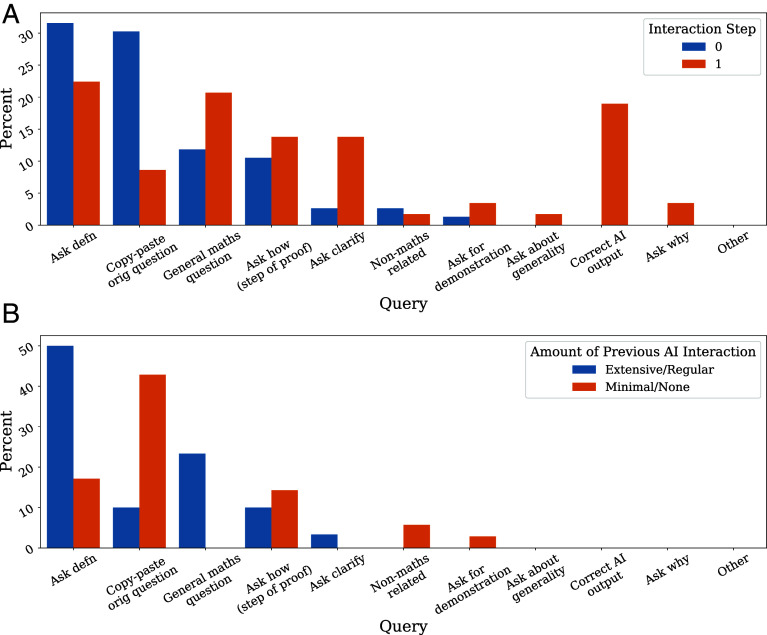
(*A*) Query profiles as a function of the interaction step. Users prefer to ask for definitions or general mathematics questions, and to paste in the full text, in the first interaction, compared to correcting the model’s output, asking why, etc. in the second interaction. Interaction step 0 is the initial interaction; step 1 is the query made after receiving the first AI response to the query made in step 0. (*B*) Query profiles for the first interaction step (i.e., step 0) as a function of the amount of experience the user has with AI systems prior to participating.

We address questions (1) and (2) by investigating “query profiles”—a “signature” of the kinds of queries made by a user (or subset of users) over a particular interaction duration. We compare query profiles for the kinds of queries participants made in the first interaction versus the second in Figure 3a to address question (1). We observe a marked difference across query profiles between the first and the second interaction, indicating the importance of studying AI systems’ behavior through interactions; query patterns can evolve in time.

To address question (2), we notice in Fig. 3*B* that users who had minimal prior AI expertise (i.e., responded that they either had never interacted with an AI system, or had only done so rarely) were more likely to simply paste in the full prompt text, in contrast to those with more experience with AI systems, who more regularly asked for decomposed tasks (e.g., asking for a definition or help on a single step of the proof). These differences in behavior hint at the potential importance of improving general AI literacy across users about the kinds of prompting behavior that induces desirable responses. Students and expert mathematicians alike stand to benefit from improved prompting tactics if it means empowering more *effective* use of this class of tools. Mathematicians at all levels can make more effective use of AI systems by learning how to delegate tasks to such systems and steering them with good prompting techniques.

We explore question (3) in *SI Appendix*, *Additional Investigations into the MathConverse Taxonomy* and find that particular queries are associated with differential correctness and helpfulness ratings. Varied model behavior in response to particular queries highlights the importance of evaluating language models through their interactions with users: if we are to deploy these systems alongside humans, we want to ensure they are robust and performant across a range of queries users naturally entertain. We leave a further systematic study of the relationship between query type and model performance for future work.

### Qualitative Observations from Case Studies.

We next include takeaways provided by each expert in relation to their freeform interactions. We then synthesize the core insights across the case studies.

### Per-Expert Conclusions.

Each domain expert provided takeaway remarks following their interactions with GPT-4.

#### Dr. William Hart.

While GPT-4 is able to regurgitate some very commonly found elementary number theoretical material and can handle straightforward problems, it has a major difficulty with algebraic manipulation and little or no ability to work on unseen problems that require backtracking, proving intermediate lemmas or extensive planning.

This is clear when GPT-4 is asked to show that

$$\begin{array}{c} 3=\sqrt{1+2\sqrt{1+3\sqrt{1+\cdots }}}. \end{array}$$

*SI Appendix*, *ProofWiki prob. 28*

A consistent problem here is an inability to write down a correct expression for a recursive relation to describe the nested radical. GPT-4 seems to be convinced that the expression under each square root is the same, so that if we write the initial expression $3=\sqrt{A}$ then we also have $3=\sqrt{1+2\sqrt{A}}$ and $3=\sqrt{1+2\sqrt{1+3\sqrt{A}}}$, etc. To probe a little deeper, GPT-4 was instead prompted in a direction that might allow it to make partial progress. The hint was given to try peeling the expression on the right-hand side one square root at a time, working backward from the desired result that the full nested radical should have the value 3 to see whether some pattern could be found in the values of the inner nested radicals. It was easy to prompt it so that it heads in that direction but on every generation it made hopeless algebraic and numerical errors, once again illustrating that very often what holds it back is high school algebra rather than the depth of the mathematics.

#### Dr. Wenda Li.

We found GPT-4’s performance at variations of several ProofWiki problems quite satisfactory: it can reliably retrieve definitions of concepts used in the problem as well as in its own proof; it can correctly assess whether loosening certain assumptions breaks the proof; it can also instantiate variables quite robustly, given the opportunity of inspection of its own answers. There have been debates (25, 26) about to what extent, shall we say, language models “understand,” given the nature of their stochastic generation. In our study, we find a couple of simple^¶^ cases where the language-model-based assistant possesses the mathematical understanding of assumptions and variable instantiations beyond mere memorization.

For instance, we asked GPT-4 to solve the following standard probability theory problem: let X be a random variable. Assume E(X)=μ for some μ∈R and $var\left(X\right)=\sigma^{2}$ for some $\sigma^{2}\in R_{>0}$. Show that for all k>0: $\mathrm{Pr}\left(\left|X-\mu \right|\geq k\sigma \right)\leq \frac{1}{k^{2}}.$ GPT-4 started by stating that we can use Chebyshev’s inequality, and then restated the problem in an almost identical way but with different variable names. This demonstrates a certain level of variable unification. We then checked whether the assistant knew how to instantiate variables by asking it whether the proof still holds when the following concrete values are assigned to k: 2, $\sqrt{2},\sqrt{2}-1,\sqrt{2}-2$, and ${\left(\sqrt{2}-2\right)}^{2}$. Human inspection finds the assistant’s behavior to be correct. The assistant can handle concrete calculations even when k is a relatively complicated number (e.g., $\sqrt{2}-1$). The model also knows that the previous derivation cannot be carried out when $k=\sqrt{2}-2$, a negative number.

#### Professor Timothy Gowers.

Many of the strengths and weaknesses we observed in GPT-4 are ones that have been commented on several times (in connection with other LLMs as well). For instance, it is not good at calculation, it has a tendency to ignore facts that do not support its main conclusion (even if it itself has generated those facts), and to invent facts that do support it (27).

When it comes to building examples, it has another weakness, which is that instead of using a process of reasoning to constrain what the example can look like and only then exhibiting the example, it prefers to start by exhibiting the example and then provide the justification that it has the desired properties. If its initial suggestion is correct, then this may be all right (though its justifications are not always correct), but often the example it gives is not correct, and it typically follows it up with a “justification” that to a trained mathematician has very obvious flaws. This behavior supports the stochastic parrot view, since mathematical constructions are typically presented in the literature in the same unhelpful style—first the example, and then the verification that it works.

One can try to use prompt engineering to discourage GPT-4 from organizing its answers in this way, for example, asking the model not to provide an example immediately but to describe a general strategy first, and only then the implementation of the strategy, or to pretend to be a student and ask it to offer hints. While these did improve its output somewhat, they did not make a significant enough difference to affect our general conclusions, so we mainly used more straightforward prompts.

A further feature that has a negative effect on the experience of interacting with GPT-4 is that even when it gives correct answers, it often describes them and checks their properties in a laborious case-by-case way, and often those checks are not even necessary. For instance, if asked to construct a 3-regular graph with eight vertices, it will typically list all the vertices and edges, and then for each vertex, it will say what its neighbours are and comment that there are three of them. Or if it needs the fact that the matrix $\left(\begin{array}{cc} 0 & 1\\ 0 & 0 \end{array}\right)$ squares to the zero matrix, instead of just asserting that it does, it will write

$$\begin{array}{c} {\left(\begin{array}{cc} 0 & 1\\ 0 & 0 \end{array}\right)}^{2}=\left(\begin{array}{cc} 0 & 1\\ 0 & 0 \end{array}\right)\left(\begin{array}{cc} 0 & 1\\ 0 & 0 \end{array}\right)=\left(\begin{array}{cc} 0 & 0\\ 0 & 0 \end{array}\right), \end{array}$$

which is adding virtually nothing to a bald assertion, since no details are given of the computation of the matrix product (not that one would want them). Similarly, it sometimes “verifies” that a matrix is symmetric by writing out that matrix and its transpose and noting that the two matrices it has written out are identical.

In the more positive direction, there were several questions that GPT-4 answered well in a way that is hard to dismiss as mere parroting. To give a simple example, if it is asked to differentiate a function, it will typically do so very competently, suggesting at least some ability to generalize. (A possible explanation for why it is good at differentiation and bad at arithmetic is that there are many more worked examples of differentiation, complete with step-by-step explanations, than there are worked examples of arithmetic.)

More examples are discussed in *SI Appendix*, *Interactive Case Studies with Experts*, with some speculations about why GPT-4 is good at them.

### Key Findings.

We now synthesize the key findings across our two evaluations: insights drawn from our MathConverse dataset collected by applying CheckMate in the domain of maths, and expert case studies, where mathematicians deeply engage with GPT-4 to solve problems from particular angles.

#### [Key finding 1] Correctness and helpfulness of model responses are related, but can diverge in interesting ways.

When a human poses a query, what is the relationship between the perceived helpfulness and correctness of a model’s answer? We find that, across all human–model interactions, helpfulness and correctness ratings are highly correlated (with Pearson correlation coefficient r=0.83). This finding corroborates a similar observation in ref. 16, wherein although correctness lags behind perceived usefulness, for both per-step and fully generated proofs, the two qualities are intimately related. This trend underscores an important point: for mathematical language models to be useful assistants, a core quality is that they should consistently produce largely mathematically correct responses. We also observe an interesting phenomenon at the extremes (Fig. 2*C*): there are cases where generations are considered incorrect but helpful, or correct but unhelpful (see Examples 4 and 3 respectively in *SI Appendix*, *Investigating the Boundary between Easy and Hard Problems*). For instance, models can generate overly verbose answers that are deemed entirely mathematically correct and only moderately helpful. In expert case study Problem Perturbation to Probe Memorization in *SI Appendix*, *Interactive Case Studies with Experts*, we see models can be good at providing definitions and interestingly can produce helpful scaffolding for a problem (e.g., the right strategy or first few steps), even if details of the full proof are incorrect. These instances reveal that to create useful assistants, increasing the mathematical correctness of these models alone is not sufficient.

#### [Key finding 2] Lack of verification can induce errors.

The CheckMate ratings are determined from a first-person perspective: participants rate the generations they receive. But what if a participant cannot appropriately verify mathematical correctness? Before interacting with models, participants are asked to indicate their confidence at solving the problem on their own. We find instances where participants who indicated low confidence (i.e., confidence ≤3; see rating scale in *SI Appendix*, *Additional Details on Survey Set-Up*) in being able to solve the problem on their own ended up rating the generation as completely correct *even when it was not*. For such examples, as well as participant testimonials of this behavior, see *SI Appendix*, *Additional Survey Observations*. In case studies, even if the model does produce a correct solution, this solution is not always arrived at in a seemingly “human-like” way. For instance, the model may follow a guess-and-check approach rather than forward planning (e.g., Examples 1, 4, and 7 in *SI Appendix*, *Interactive Case Studies with Experts*). However, guess-and-check cannot work well if one cannot “check” solutions. Indeed, we see that, in general, challenges with algebraic manipulation plague in- and out-of-distribution performance (e.g., examples in *SI Appendix*, *Interactive Case Studies with Expert, Number Theory Evaluation*). Further, our studies highlight issues in interaction even when the *human user* attempts to correct the model. One case study illuminated intriguing behavior when the model was queried about uncertainty: the model began to apologize despite having been correct (*SI Appendix*, *Interactive Case Studies with Expert, Problem Perturbation to Probe Memorisation*).

#### [Key finding 3] The double-edged sword of reliance on memorized solutions.

Learning concepts and definitions is an important aspect of acquiring world knowledge. The MathConverse taxonomy revealed that queries about mathematical definitions are popular, and responses received are considered one of the most helpful among all the categories. In contrast to definitions, solutions to specific problems should ideally be understood in a fashion that can generalize and not be completely memorized (*SI Appendix*, *Interactive Case Studies with Experts*). By probing GPT-4 capabilities on slightly novel problems or those which involve building examples, we notice the model’s tendency to overrely on plausibly memorized examples or patterns. We caution that we cannot be definitely sure whether these examples are indeed “memorized” without direct access to the models’ training data. However, from the behavior, we have a strong suspicion this is the case.

## Discussion

We compile our key observations from both our structured and instance-based evaluations into a series of actionable takeaways, which—given the interdisciplinary nature of our study—we hope will appeal to a wide audience. We tailor these takeaways to audiences from different fields. To offer balance, we first note that the best LLMs we investigate do demonstrate some nontrivial ability in collaborating helpfully and correctly with users on undergraduate-level mathematical problems (Fig. 2*B*). Should the user be able to assess the validity of LLM-generated responses, they can meaningfully assist on some problems. Even if the answers are memorized and can be found somewhere on the internet, LLMs have the advantage of being flexible in their inputs and outputs over traditional search engines. We close with limitations of our methodology.

### Takeaways for ML Developers.

#### Enable models to communicate calibrated uncertainty and uptake corrections.

We observe cases where people attempted to correct the model when it made an error, the model apologized and proceeded to give an answer without the necessary corrections or asking for clarification. The pattern often repeated itself until the user seemed to get bored and aborted. To improve user experience, systems that can adequately respond to user corrections, for example, through uncertainty calibration (28–30), are compelling (31–35). Indeed, in the models we explored, it was not clear when the model was unsure. We include a discussion with participants about these challenges in a postsurvey questionnaire (*SI Appendix*, *Post-Survey Testimonials from Participants*). Communicating uncertainty is critical to ensure users know when they can trust the model output (28, 36), and help calibrate appropriate levels of trust (37, 38). However, obtaining accurate, calibrated uncertainty estimates from LLMs can be a difficult endeavor (39, 40).

#### Enable provision of rationales.

Several participants in MathConverse asked “why” a model undertook a particular proof step. Expanding on the justification for a choice could be a valuable educational tool. Generating compelling explanations, on-the-fly and on-request [provided those explanations are indeed representative and not misleading (41–45)], seems promising and desirable to explore in order to further boost the utility of these systems in partnership with mathematicians.

#### Strive for conciseness.

Both our survey and our expert case studies find that—while mathematical correctness appears to often be a crucial component of useful assistance in higher-level mathematics—it is not always sufficient. Responses that were overly verbose were sometimes deemed less helpful. Designing systems that generate concise responses to mathematical queries seems a promising future direction, particularly if coupled with the capability of showing its “work” if needed (related to rationales, see above). The applicability of such response behavior to other domains than mathematics remains to be investigated: it may be that responses of different degrees of verbosity are preferred in different domains [e.g., in medicine, longer responses laden with empathy may be preferable (8)].

### Takeaways for Mathematicians (Students, Educators, and Researchers).

#### Pay attention.

LLM are capable of generating remarkably compelling natural language—an incredible technical feat which ought not to be dismissed and can be helpful as we see in both our studies. However, such prowess belies the potential for coaxing the reader into not recognizing errors. Be careful not to fall into the trap of lazy checking (*SI Appendix*, *Post-Survey Testimonials from Participants* in participant testimonials). This is worth keeping in mind for users learning from or evaluating the generations of LLMs, for example, students and assignment markers. It is worth being cognizant of the risk of automation bias, that is, where a user may inappropriately overrely on the output of a model simply because it came from a model (46).

#### Take a nuanced view of when these models can help.

Reinforcing similar findings from ref. 47, we observe in this work that LLMs can be useful for retrieving definitions (*SI Appendix*, *Additional Taxonomy Observations*), and can occasionally provide a valuable scaffold for how to approach a problem (*SI Appendix*, *Additional Survey Observations* and *Interactive Case Studies with Experts*). It is important not to presume that a model which performs well in one realm of the task space will surely perform well in another (48, 49). Counterintuitively—á la Moravec’s Paradox (50)—it is possible that models will succeed at tasks perceived challenging by humans, but fail at tasks humans consider easy (e.g., derivation versus algebraic manipulation). Mathematicians can take advantage of our query profiles to learn how they could adapt their behaviors to get more out of language models for mathematics.

#### Be cautious when using current LLMs (alone) for heavy algebraic manipulations.

In particular, our studies further underscore the challenges of present models at algebraic manipulation, corroborating prior work (47, 51, 52). We believe it is therefore important that mathematicians take care if using these systems for tasks which involve substantial algebraic manipulations. We do not explore plug-ins (53) in this paper, nor alternative hybrid neuro-symbolic approaches (e.g., refs. 54, 55, 56, 57, 58, 59), which may prove a useful salve for some of these failure mode.

### Takeaways for LLM Development, Evaluation, and Deployment.

We conclude with broad takeaways for anyone developing, evaluating, or considering deploying LLMs in practice.

#### Carefully discern when assistance is needed (or even worth utilizing).

To build complementary systems (60), understanding when an AI-based assistant is helpful is of utmost importance: seldom will such an assistant be helpful in all settings (48). An important question is which settings such an assistant can be useful without undermining the agency of the mathematician, for example, of the kind already being proposed when considering using LLMs in coursework (61). Future work would benefit from considering how to build usable assistants that optimize for complementarity, providing support as and when needed (62).

#### Collaboration between ML practitioners and domain experts is valuable.

Conducting investigations in partnership with domain experts can be especially fruitful for characterizing model behavior (9, 18, 19), particularly by designing entirely new tasks, as our expert case studies demonstrate. We encourage forming such interdisciplinary partnerships in and beyond mathematics.

#### Incorporate interactivity into LLM capability assessments.

To truly comprehend the landscape of an LLM’s capabilities, we believe it is paramount to incorporate interactive evaluations. Our work further drives home the importance of interactive evaluation as a way to gain deeper insights into the strengths and weaknesses of these models, and probe characteristics which may be preferable for assistive settings. However, as we highlight here, the interactive study of LLMs not only serves to characterize model behavior, but it identifies ways in which *humans* may themselves choose to interact with these models and actually use these systems (63). A wave of works increasingly illuminates the sensitivity of these models to the choice of prompts (64–66). As such, it is important to consider the form and content of queries that humans may use to interact with these systems both to design systems more adapted to particular user queries, and to inform users of best practices. It may be valuable for system maintainers to recognize whether or not users are leveraging these tactics, in order to help better inform the techniques for boosting the quality of the response for their query.

We hope to see more works like ours and those of others (9, 11, 67) that study LLMs in the context of human–computer interactions. CheckMate offers a place to start, potentially complemented by free-form evaluation of the kind we conduct in our expert case studies.

## Limitations

While our study provides insights into how mathematicians may use language models—and opens doors for future interactive evaluation—our survey is simply an initial step in evaluating LLMs for mathematical assistance. Our sample size is small but informative; we consider MathConverse to be a preliminary dataset to spark further methodological and deployment-time considerations. For instance, we encourage scaling the number of problems per mathematical topic to better elucidate differential model performance across branches of mathematics (*SI Appendix*, *Interaction Ratings by Mathematics Topic*). Additionally, we ask each participant to rate generations provided during their own interaction trace; while this permits first-person evaluation of the kind called for in ref. 11, for those who do not already know how to solve the problem this means that they may be falsely judging the correctness of the generation. A sensible next step would be twofold: deploying our evaluation platform with students who have not already solved such problems, and sending the interaction traces off for external evaluation as well. We also encourage a reassessment of mathematician interactions over time; it is quite possible—in fact likely—that the kinds of interactions humans make with these systems will evolve as their capabilities grow. Additionally, our taxonomy categories are nonexhaustive; alternative categorizations are possible. Nevertheless, we found our categorization sufficient to draw helpful findings.

While our case studies offer invaluable insight into the prompting behavior of domain experts and further characterization of model performance, each individual may bring to bear their own expectations about models’ strengths and weaknesses, which could seep into the way probing behavior is chosen and model outputs are interpreted. We emphasize that, as with the survey results, these insights ought not to be taken as a firm testament about the capabilities nor potential of these models, much less all language models (we only consider a handful, and all from the OpenAI family). Rather, we hope the evaluation toolkits, expanded on from our studies, pave the way for further research into the use of LLMs as assistants for problem-solving, in mathematics and beyond.

## Conclusion

As LLMs are increasingly deployed in human-facing settings where they may serve as assistants, it is paramount that evaluation of their efficacy fundamentally includes evaluation in an interactive context (11). As we demonstrate, these interactive evaluations can be structured (e.g., leveraging CheckMate) or free-form (e.g., through sourced domain expert or target user interactions). LLMs, and foundation models broadly, are complex and often surprising in their behavior; so are humans. Hence characterizing potential failure modes in LLM and human interactions necessitates a multifactorial evaluation approach, which includes both interactive evaluation and classical, static-snapshot evaluation (10). Through our study, we extract insights which we hope can inform careful design and deployment when considering leveraging LLM-based mathematics assistants and reasoning engines. We believe that our study paves the way for further evaluation of the use of foundation models in mathematics and other domains, particularly through closer collaboration with domain experts.

## Materials and Methods

### CheckMate: Adaptable Platform for Interactive Evaluation.

We introduce CheckMate as an adaptable prototype platform to support *interactive* evaluation of language models.^#^ Humans can interact with and rate text generated by language models, and CheckMate records the “interaction traces.”^‖^

We design CheckMate to support two flavors of evaluation: studying the interactions with a single model, and studying preference across a bank of models. In this section, we first introduce the rating scheme for a single model. Then, we discuss how we support comparative evaluation over a suite of models. We focus on the domain of mathematical theorem proving; however, CheckMate can be extended more broadly (*SI Appendix*, *User Guide for CheckMate*).

#### Evaluation for a single model.

Evaluation begins with permitting the participant to freely interact with the model in order to solve a problem. We encourage participants to imagine they are trying to solve the problem—and elicit assistance. The participant can continue to explore assistance for up to 20 interaction exchanges.^**^ When the participant is satisfied with the level of assistance (or sufficiently unsatisfied that they wish to terminate the interaction), they proceed to evaluate *each step* of their entire interaction.

We design CheckMate to support a multidimensional evaluation over the interaction trace for the successive human query-model generation pairs. At present, the platform is designed with a mix of Likert scales and radio buttons (*SI Appendix*, *Additional Details on Survey Set-Up* and *User Guide for CheckMate*). However, CheckMate can be readily extended with alternative rating types, for instance, to handle individual error profiling (16) or additional interaction metrics, as proposed in refs. 12 and 11.

#### Comparative evaluation across models.

With an ever-growing suite of language models available for humans to leverage, it is important to compare capabilities across models, and how these compare to previous versions of a given model. When done, such comparisons typically involve single snapshots. CheckMate permits the study of preference *over the interaction trace* and can serve as a valuable tool to explore the evolution of assistance potential.

In CheckMate, participants provide a rank order over which model they preferred interacting with, after they have interacted with two or more models. This instantiation of the platform is set up such that participants interact with a different task per model (to avoid “bleed over” effects when considering the same problem multiple times). However, alternative designs, for example, rating models per task, or subsampling the models to evaluate, are possible adaptations to our paradigm (*SI Appendix*, *User Guide for CheckMate*). Importantly, participants are *blind* to which model they are evaluating at any time; this ensures they are not biased by preconceived notions of which model may be more performative.

In the rank order, participants can assign the same rank if they are unsure which model they prefer. Future work could consider more expansive comparative preference evaluation. We provide further details on CheckMate and hosting our survey in *SI Appendix*, *User Guide for CheckMate*.

#### Instantiating CheckMate for mathematics to collect MathConverse.

Survey participants are asked to prove a mathematical statement and to use an AI system to assist them in any way to carry out this task. Such free-form interactions can range from asking for help on the entire problem, to clarifying definitions, or asking for an explanation for a particular generated proof step. Participants are not provided with possible interaction behaviors in advance to avoid priming, as one of our key goals of the study was to elucidate the kinds of queries participants may naturally employ. When the participant is satisfied with the level of assistance (or sufficiently unsatisfied that they wish to terminate the interaction), they proceed to evaluate *each step* of their entire interaction. Participants solve a different problem for three models (Instruct-GPT, ChatGPT, and GPT-4), where the order of the models is shuffled and participants are blind to which model they are interacting with.

We next describe our task set-up over which we conduct evaluations. The study was conducted under the approval of the University of Cambridge Computer Science Ethics Division; all participants provided informed consent. *SI Appendix*, *Example Interface Screens* includes interface screens of CheckMate for mathematics.

#### Tasks.

We select 54 problems from ProofWiki, a corpus of *undergraduate-level* mathematics problems.^††^ Nine problems are selected from each of six mathematics topics (linear algebra, number theory, probability theory, algebra, topology, and group theory). We select these topics to span a range of subject areas in typical undergraduate mathematical curricula.^‡‡^

#### Rating scales.

Participants evaluate the *perceived helpfulness* and *mathematical correctness* of each step, selecting one “preference” and one “quality” metric, as defined in ref. 11. Cognitive load and biases are kept in mind at each stage of the design, for example, lightening the number of ratings per page, and randomizing model rating order to reduce possible ordering effects. Ratings are provided on a 7-point Likert scale, with the width chosen to ameliorate potential rating collapse [i.e., the phenomenon where participants hesitate to use scale endpoints (68)]. We select only two factors per step to avoid excess cognitive load while rating. Before responding, participants specify their confidence in being able to solve the problem on their own. After interacting with the three models on three different problems, participants are shown the full interaction traces with each model and (blind to the true underlying model types) indicate their rating about which model they would prefer as an assistant via a dropdown bar. We include full details of the scales in *SI Appendix*, *Survey Set-Up*. For all quantitative analyses (except for analyzing users’ stopping behavior in Fig. 2*D*) we filter out generations rated as zero for mathematical correctness, as that means that no mathematically relevant content was included; we find that these are typically responses to greetings or exclamations (e.g., after the user has thanked the model—see released data).

#### Language model selection and set-up.

Participants evaluate three popular language models: InstructGPT (20), ChatGPT (5), and GPT-4 (21) in chat mode. As new language models are introduced, the methodology of designing optimal prompts is rapidly evolving [e.g., to name a few (64–66)]. Since we are studying how *real domain users* (i.e., undergraduate mathematics students) would interact with these systems *in-the-wild*, we keep a sparse base prompt, only asking the model to be a helpful mathematical assistant in the prompt. Further details for the experimental setup can be found in *SI Appendix*, *Survey Set-Up*.

#### Participants.

We recruit volunteers to participate in our evaluation. In total, we received 25 entries comprising 261 human–model interactions; since we did not store a unique participant identifier, for example, the IP address, for privacy reasons (*SI Appendix*, *Survey Set-Up*), we cannot confirm that these are exactly 25 unique individuals. The mathematicians have experience levels ranging from current undergraduate students up to expert mathematics professors; based on our recruiting practice (*SI Appendix*, *Participant Recruitment and Additional Details*) even participants without a formal mathematics degree likely have some exposure to high-level mathematics. Each participant chooses one of the six topics and can evaluate as many questions as they like (up to the maximum of 9). Note, the range of expertise of our participants—up to world-class experts—coupled with the fact that our problems sit at a level where students majoring in mathematics might find them in textbooks or as exercises, means that some participants may be able to solve the problems already, others may not. If a participant knows how to solve the problem, we ask that they *imagine* what kind of assistance they would like had they been at the experience level of someone who does not know how to solve the problem. More details on recruitment, data processing, and expertise can be found in *SI Appendix*, *Survey Set-Up*.

### Deriving a Taxonomy from MathConverse.

We observe a wide spectrum of mathematicians’ interactions with AI assistants in MathConverse. We derive an initial taxonomy of these interactions and annotate each interaction according to the taxonomy. To build the taxonomy, a subset of our author team manually inspected each interaction (i.e., the user’s query) and identified 10 broad categories in which most interactions seemed to fall. These categories are specified in *Taxonomising User Behaviour*. We included an additional “Other” bucket for queries which did not fall into one of the 10; cases that fell into such a category were discussed among the annotators. Four of our paper’s coauthors then manually annotated each user query into these buckets. These annotators were asked to mark whether an interaction fell into a bucket, with an option to specify whether they were unsure. Each interaction was annotated by a single annotator; however, in the case of confusion or discrepancies, annotators discussed and came to an agreed coding. We release the annotated taxonomy in our https://github.com/collinskatie/checkmate. Full instructions given to annotators are included in *SI Appendix*, *Additional Details on Taxonomy Construction*.

### Interactive Case Studies with Experts.

While structured interactive evaluation permits nice quantitative findings, to deeply understand the capability of LLMs—in the context of mathematics and beyond—free-form interaction, like instance-level evaluation (10) can be particularly revealing. Here, we expand the scope of the evaluation and uncover glimmers of the boundary between problems that GPT-4 finds easy and those it seems to find hard. In our quantitative study with CheckMate, we observed a close relationship between mathematical correctness and perceived usefulness. But since correlation is not causation, we further explore the broader *mathematical reasoning capabilities* of these models as a bedrock to inform their utility as proof assistants. Our goal is 1) to offer one of real expert mathematician interactive case studies with LLMs to help guide the design of better mathematical assistants and inform their safe, trustworthy use by helping characterize their limitations; 2) to pave the way for further interactive evaluations; and 3) to highlight patterns of human–computer interaction not previously known to the community, particularly when the humans interacting are domain-leading experts. We hope the work will be of interest to ML engineers and researchers, cognitive scientists, human–computer interaction specialists, mathematicians, educators, and beyond.

A complete transcript of interactions for each case study example is included in Dataset S3. We maintain the original text of each case study author for authenticity, with only minor edits for precision and coherence. For the interactions with ProofWiki problems, we also host them with a https://albertqjiang.github.io/casestudies/pages/proofwiki21.html for clearer visualization.

First, our recruited experts conduct a deeper dive into some of the problems we explored in our structured evaluation with CheckMateover ProofWiki problems. Specifically, we use the problems as a playground to explore how much the model seems to “know” about relevant concepts and further characterize what interactions can yield better (or worse) performance and assistance experience. We focus on GPT-4 (in chat mode) because it showed the strongest overall performance in our quantitative study with CheckMate. The first case study is provided by Dr. William Hart, a number theorist by training; the second is contributed by Dr. Wenda Li, a computer scientist with expertise in formal mathematics, and the third one was conducted with Prof. Timothy Gowers, a Fields medalist and Professor in mathematics. Further details can be found in *SI Appendix*, *Interactive Case Studies with Experts*, with all raw interaction traces included in Dataset S3.

## Supplementary Material

Appendix 01 (PDF)

Dataset S01 (CSV)

Dataset S02 (CSV)

Dataset S03 (PDF)

## Data Availability

All data and code are already deposited as open-access at our repository: GitHub (https://github.com/collinskatie/checkmate) ( 69). All other data are included in the manuscript and/or supporting information.

## Footnotes

^*^The name alludes to the interactive manner of the evaluation, or “checking,” to be the kind you may do with a “mate.”

^†^At the time of our study, ChatGPT was often referred to as the 3.5 version. Specifically, we employed the “gpt-3.5-turbo” model, according to the OpenAI https://platform.openai.com/docs/api-reference/chat/create.

^‡^The dataset can be found at https://github.com/collinskatie/checkmate.

^§^This generation is from GPT-4; recall, participants were not told which model they were interacting with and rating, or even which models they could have been interacting with.

^¶^Note, “simple” here is relative to that of a trained mathematician.

^#^Base code can be found in our repository: https://github.com/collinskatie/checkmate.

^‖^We borrow the terminology of refs. 11 and 67 for “interaction traces.”

^**^We chose a limit of 20 expecting that participants may fatigue beyond that point, and to guard against the possibility that a participant could try to interact unfettered with the model for an extended period of time.

^††^https://proofwiki.org.

^‡‡^The problems can be found at https://github.com/collinskatie/checkmate/tree/main/data/problems.
