# Supplementary material for: Evaluating language models for mathematics through interactions
Source: Proc Natl Acad Sci U S A. 2024 Jun 3;121(24):e2318124121. doi: 10.1073/pnas.2318124121 (PMC11181017; doi:10.1073/pnas.2318124121)
Supplement: Supplementary file 1 — Appendix 01 (PDF) [file pnas.2318124121.sapp.pdf]

Supporting Information for

Evaluating Language Models for Mathematics through Interactions

Katherine M. Collins*, Albert Q. Jiang*, Simon Frieder, Lionel Wong, Miri Zilka, Umang Bhatt, Thomas Lukasiewicz, Yuhuai Wu, Joshua B. Tenenbaum, William Hart, Timothy Gowers, Wenda Li, Adrian Weller† and Mateja Jamnik†

Katherine M. Collins and Albert Q. Jiang.
E-mail: kmc61@cam.ac.uk, qj213cam.ac.uk

This PDF file includes:

Supporting text
Figs. S1 to S7
Table S1
Legends for Dataset S1 to S3
Legend for Software S1
SI References

Other supporting materials for this manuscript include the following:

Datasets S1 to S3
Software S1

Supporting Information Text

Primer on Proof Assistants

Push-button automation in mathematics has long been a dream of many, replete with an extensive history (1–11). The initial goal of specifying problems in a sufficiently expressive logic and solving them routinely with fully automated theorem provers was not readily realised (12). This led to a shift in focus towards *interactive* theorem provers (ITPs), or *"proof assistants"*: humans specify the high-level structures of proofs and rely on machines to close out tiny details and weave together components (13–15). In this way, humans and machines collaborate to produce mechanically-verifiable proofs. However, adoption in the mathematical community has been slow as ITPs traditionally suffered from two weak points. First, given the systems' precise nature and relatively weak automation, writing formal proofs in interactive theorem provers is an extremely arduous and expensive task (e.g., verifying the correctness of Hales's proof of the Kepler conjecture (16) took a group of mathematicians and computer scientists eleven years (17)). Secondly, while ITPs can check whether proofs are correct, they often provide little assistance for finding the proofs to truly difficult problems: people usually understand informal proofs before translating them into formal ones instead of directly working out formal proofs.

With the rise of language models, the role of machines in assisting mathematicians is undergoing reconsideration: can language models also automate high-level mathematical reasoning? While great strides have been made (18**?** –30), consistent full and correct automation has not yet been met; at present, many language models, on their own, struggle not only on truly "hard" graduate level problems (31), but also on simple mathematical concepts such as counting (32).

However, this does not rule out the possibility that they can be *useful*. The potential role of computerised mathematical *assistants* is being re-imagined – human-machine partnerships where neither comes up with the proof alone. To adequately begin to explore these kinds of relationships necessitates, we need more studies of actual mathematicians' interactions with these systems. While the assistance potential of ITPs has been evaluated with humans (33–35), we aim for a platform to facilitate rapid evaluation of LLMs in particular. The space of LLMs is changing rapidly, from new base models (36–39) to new ways of linking them together and leveraging the output of these systems, e.g., (40–43), to new prompting techniques (44–46), and more. As such, there is a need for a reliable scaffold to permit interactive evaluation of these human-machine interaction (47–51). It is this notion – interactive evaluation of humans with LLMs, specifically in the context of proof assistance – that our paper focuses on.

An Example Survey Problem

Let $x_1, x_2, \ldots, x_n \in \mathbb{R}$ be real numbers which are all positive.
Let A_n be the arithmetic mean of $x_1, x_2, \ldots, x_n$.
Let G_n be the geometric mean of $x_1, x_2, \ldots, x_n$.
Show that

$$A_n \geq G_n$$

with equality holding iff:

$$\forall i, j \in \{1, 2, \ldots, n\} : x_i = x_j,$$

that is, iff all terms are equal.

An Incorrect but Helpful Response

If U is a linear transformation between n-dimensional vector spaces, how do I show that U and its transpose have the same rank and nullity?

To show that a linear transformation U and its transpose U^T have the same rank and nullity, we can use the Rank-Nullity Theorem and properties of linear transformations.

Let $U\colon V \to W$ be a linear transformation between n-dimensional vector spaces V and W, with $\dim(V) = n$ and $\dim(W) = m$. Let $U^T\colon W* \to V*$ be the transpose of U, where $W*$ and $V*$ are the dual spaces of W and V, respectively.

The Rank-Nullity Theorem states that for any linear transformation $T\colon X \to Y$ between vector spaces X and Y, the following equation holds:

$\dim(X) = \text{rank}(T) + \text{nullity}(T)$

Now, let's apply the Rank-Nullity Theorem to U and U^T:

1. For U: $V \to W$, we have:

$\dim(V) = \text{rank}(U) + \text{nullity}(U)$

2. For U^T: $W* \to V*$, we have:

$\dim(W*) = \text{rank}(U^T) + \text{nullity}(U^T)$

Since the dimensions of the dual spaces are equal to the dimensions of the original spaces ($\dim(W*) = m$ and $\dim(V*) = n$), we can rewrite the equation for U^T as:

$\dim(W) = \text{rank}(U^T) + \text{nullity}(U^T)$

Now, we need to show that $\text{rank}(U) = \text{rank}(U^T)$ and $\text{nullity}(U) = \text{nullity}(U^T)$.

First, let's consider the rank. The rank of a linear transformation is the dimension of its image (or column) space. Since U and U^T are transposes of each other, their column spaces are the same, which means their rank is the same:

$\text{rank}(U) = \text{rank}(U^T)$

Next, let's consider the nullity. The nullity of a linear transformation is the dimension of its kernel (or null) space. The kernel of U is the set of vectors in V that are mapped to the zero vector in W, and the kernel of U^T is the set of vectors in $W*$ that are mapped to the zero vector in $V*$. Since the dimensions of V and W are the same, the dimensions of their dual spaces $V*$ and $W*$ are also the same. Therefore, the null spaces of U and U^T have the same dimension:

$\text{nullity}(U) = \text{nullity}(U^T)$

In conclusion, we have shown that U and its transpose U^T have the same rank and
`[GENERATION TERMINATED DUE TO LENGTH LIMIT]`

An Overly Verbose Response

The following conversation was between a human and GPT-4, the response by the latter was later considered overly verbose:

How should I go about finding such polynomial?

To find a polynomial, you need to have some information about its properties, such as its degree, coefficients, roots, or any specific behaviour it exhibits. Here are some general steps to follow:

1. Determine the degree of the polynomial: The degree of the polynomial is the highest power of the variable (usually x) in the polynomial. It determines the number of roots the polynomial has and the overall shape of its graph.

2. Identify any known roots: If you know any roots of the polynomial (i.e., values of x for which the polynomial equals zero), you can use them to write the polynomial in factored form. For example, if a polynomial has roots at x = 1 and x = -2, it can be written as $P(x) = a(x - 1)(x + 2)$, where a is a constant.

3. Use any additional information: If you have any other information about the polynomial, such as its value at a specific point or the behaviour of its graph, use this information to further refine the polynomial. For example, if you know that the polynomial passes through the point (3, 4), you can plug in x = 3 and y = 4 into the polynomial equation and solve for any unknown coefficients.

4. Write the polynomial in standard form: Once you have determined the polynomial's factored form and found any unknown coefficients, you can expand the polynomial to write it in standard form (i.e., as a sum of terms with decreasing powers of x).

If you can provide more specific information about the polynomial you are trying to find, I can help guide you through the process more precisely.

A Response about Intuition

The following conversation was between a participant and GPT-4, where the participant asks the model to explain the intuition behind its generated proof. The part of the conversation prior to this human query is omitted; all interaction traces with participants are included in the raw `MathConverse` data at our repository.

What is the intuition behind the proof of this statement?

The intuition behind the proof of the Weak Law of Large Numbers (WLLN) is that as the sample size increases, the sample mean becomes a better and more stable estimator of the population mean. This is because the influence of individual observations diminishes as more data points are included in the sample.

Here's a more detailed intuitive explanation:

1. The WLLN states that the sample mean converges in probability to the population mean. In other words, as the sample size (n) increases, the probability that the sample mean ($\bar{X}n$) is close to the population mean (μ) approaches 1.

2. The random variables X1, X2, ..., Xn are independent and identically distributed (i.i.d.), which means they have the same distribution and are not affected by each other. This ensures that the sample mean is an unbiased estimator of the population mean, i.e., $E(\bar{X}) = \mu$.

3. The proof of the WLLN often relies on Chebyshev's inequality, which provides an upper bound on the probability that a random variable deviates from its mean by more than a certain amount. Intuitively, Chebyshev's inequality states that most of the probability mass of a random variable is concentrated around its mean, and the probability of large deviations decreases as the deviation size increases.

4. When applying Chebyshev's inequality to the sample mean, we find that the probability of the sample mean deviating from the population mean by more than a certain amount (ϵ) decreases as the sample size (n) increases. This is because the variance of the sample mean ($\mathrm{Var}(\bar{X}n) = \frac{\sigma^2}{n}$) decreases with the sample size, making the sample mean more concentrated around the population mean.

5. As n approaches infinity, the probability of the sample mean deviating from the population mean by more than ϵ approaches 0, which means that the sample mean converges in probability to the population mean.

In summary, the intuition behind the proof of the WLLN is that as the sample size increases, the sample mean becomes a more accurate and stable estimator of the population mean, and the probability of large deviations between the sample mean and the population mean decreases. This is due to the properties of independent and identically distributed random variables and the concentration of probability mass around the mean.

User Guide for `CheckMate`

We include a user guide for those wishing to extend `CheckMate` for their own interactive evaluation tasks. Our guide maps directly onto our open-source repository.

We describe several hypothetical scenarios in which someone is extending `CheckMate`. We emphasise that any application of such interaction with real human participants ought to check with the affiliated institutions' ethics review.

Hypothetical extensions.

New base tasks If you would like to have different tasks than ProofWiki, you can replace `data/problems/` with your files. The most straightforward way to do so is to have each new base task as its own file; we encourage files to be associated with a unique task number ID. If you have a grouping for the tasks which you would like a participant to be able to select (e.g., in our case, participants selected a mathematical topic and were only shown problems from that topic), you can code which problems are associated with which topic in `problems_per_topic` in `experiment.py`.

New rating scales If you would like to change the ratings, you can change the question and add different rating endpoints in `constants.py`, and change the Gradio object type in `pipeline_for_model` in `experiment.py` (e.g., see that Likert scales like "ai_corr_rating" which holds user correctness per interaction is coded as a Radio button). That is all that is needed if you keep two ratings per generation; however, if you would like to add or remove ratings, you need to do a bit more programming. You can add new a new rating by going into `experiment.py` and adding a Gradio radio element with the new rating options. You can refer to how `ai_corr_rating` is constructed and stored. Similarly, to delete a rating, the easiest approach is to find

how its associated rating options are used in `experiment.py` and delete all related variables. Note, if changing ratings, you will also need to update saving and variable visibility toggling, which is somewhat hard-coded per challenges noted below.

Different models to evaluate If you would like to vary the models which are evaluated, you can do so by changing the model tags in `model_options` in `constants.py`, and adding associated API calls in `model_generate.py`. Note, if you would like to use a model with guarded API access, *you will need to provide your own API key*. Additional models can be used if you substitute the OpenAI calls with calls to the additional models. You can play with the prompt used in `model_generate.py`, and temperature can be varied if using the OpenAI Completion API in `constants.py`.

If you would like a different set-up than evaluating the preferences across three models, you can change the number of models before preferences by varying the models passed to `a_single_problem`.

If you would instead like to remove the final preferences entirely, and just have a rating per model, you can remove the "Final prefence" tab in `a_single_problem` in `experiment.py`.

New instructions It is particularly straightforward to swap out new experiment instructions. To do so, you can go to `constants.py` and see `plaintxt_instructions`. Each entry in the array is a new instruction page. You can track where this information is propogated through `instruction_pages` in `constants.py` and then in `experiment.py` as you adjust your instructions. Note, as we were circulating the study through volunteer channels, we also included text in a Google Webpage. This can be made quickly through Google Pages, and requires no coding experience.

Hosting `CheckMate` can be adapted to be hosted on a personal server or using a public offering, such as Hugging Face Space (52). Choosing a server that is geographically closer to the target audience may help reduce unwanted interaction lag.

Implementation Challenges. Additionally, we detail several implementation challenges we faced. While the interface can be readily co-opted for new tasks as discussed above, we hope that shedding light on some of the challenges we faced when designing the platform may be of use to those who wish to further customise `CheckMate` for their own tasks. Note, as the Gradio platform is rapidly evolving, these implementation challenges may become obsolete shortly; we recommend checking the latest capabilities.

- Multiple pages: A common structure in psychological studies is to have multiple pages of instructions and different tasks; the biggest challenge we faced was how to design multiple pages. In particular, the way that Gradio seemed to work – at least at the time of our construction – is that variables needed to be shared across pages. As such, we had to instantiate all key variables and the start and then iteratively show/hide components. It is possible that a more efficient way already was possible, or that one will be developed after the release of this working paper. At present, however, our code does offer a functional starting point for multiple pages.

- Saving: Relatedly, we needed to ensure that participants' responses were saved over the course of the study; however, due to state changes, this meant that we needed to be extra careful with saving and deduplicating the final results. Care in this step for any future users is essential.

- Latency: A common annoyance we heard from participants is that the study took too long to load. There are two core reasons for this: (1) each interaction queried a language model API which comes with inevitable overhead at present, and (2) as the web server was hosted in western United States, participants seeking to partake from other countries, e.g., France, reported higher latency. Frustrating wait times may help explain the low number of problems each participant took on. Better measuring and addressing latency, as suggested in (50, 53), are important grounds for future work.

Additional Details on Survey Set-Up

We hosted the study using Gradio (54). We ran the study between April 7, 2023 and April 24, 2023. We circulated a landing page, which included a link to the actual instance of `CheckMate`.

Rating Scales. We include the labels which were presented to participants for each of the rating dimensions, along with the question. Before participating, users rated their confidence in being able to solve the problem themselves. After interacting with a single model, they rated the correctness and perceived usefulness *of each generation*. And after interacting with the set of three models, they rated overall preference.

Before Generation. **"Question: Before interacting with the AI – how confident are you that *you* could solve this problem *entirely on your own*, with your current knowledge base and no extra assistance?"**

- "(0) Definitely could not solve on my own"
- "(1) Very unlikely to be able to solve on my own"
- "(2) Unlikely to be able to solve on my own"
- "(3) May be able to solve on my own"
- "(4) Likely be able to solve on my own"

- "(5) Very likely to be able to solve on my own"
- "(6) Definitely can solve on my own"

Per Generation Ratings, Per Model. **"Question 1: How correct (i.e., mathematically sound) is the generation?"**

- "(0) N/A - this response does not contain any mathematical information"
- "(1) Completely incorrect or nonsensical"
- "(2) Multiple critical maths errors"
- "(3) At least one critical math error or multiple small errors"
- "(4) One or more minor errors, but otherwise mostly correct"
- "(5) One or two minor errors, but almost entirely correct"
- "(6) Completely correct"

"Question 2: How helpful would this AI generated response be towards helping someone solve this problem? If you already know how to solve the problem, evaluate this as if you were an undergraduate mathematics student encountering this problem for the first time."

- "(0) Actively harmful"
- "(1) Very harmful"
- "(2) Somewhat harmful"
- "(3) Unlikely to help, but unlikely to hurt"
- "(4) Somewhat helpful"
- "(5) Very helpful"
- "(6) Definitely helpful"

Cross-Model Preference. After interacting blindly with the three models, participants were asked **"You will now rate which model(s) you prefer as a mathematical assistant. 1 = best, 3 = worst. You can assign the same rating if you think two (or more) models tied"**. Ratings were provided via drop-down options (including 1, 2, 3).

Participant Recruitment and Additional Details. We provide additional details on our participant recruitment and `MathConverse` data processing. All participation was unpaid and entirely voluntary, and provided informed consent. Participants were recruited via authors' connections: We circulated the study through the University of Cambridge Mathematics Department Mailing List, as well as the team channel for the Human-Oriented Automated Theorem Proving project. The study was also posted on the Lean Zulip channel, where a large community of mathematicians and computer scientists congregate to discuss issues related to formal mathematics. Additionally, the study was circulated amongst mathematics friends at MIT, Oxford, University College London, University of Vienna, École Polytechnique, and Carnegie Mellon University, and elsewhere in Berlin and Paris. We also sent the study to some machine learning students who had mathematics background at the University of Cambridge, MIT, and Princeton; here, participants may not have a formal degree in mathematics but have usually been exposed to mathematics to some degree (i.e., they are not pure amateurs). It is not clear who uptook the survey, as we did not save any personal information, beyond a participant's level of formal mathematics education and experience playing with AI systems, for privacy reasons[*]. However, we connected with some of the experienced mathematicians who participated for post-survey testimonials about their experience during the study (see the Post-Survey Testimonials below). In the end, we achieved decent coverage across the question topics (see Figure S1).

Additionally, of the 25 entries received, 15 resulted in at least one full round of model preferences (i.e., interacting with all three models, and ranking preference); we keep all 25 in the individual interaction analyses as they still provide rich data. Specifically, in cases where preference ratings were not provided, or were provided but a participant had not interacted with all three models (in which case, preference scores are meaningless), we keep the scores for the individual ratings and ignored the preference ratings. In one instance, a participant did not rate all generations from the model; we also discarded this case. We emphasise for anyone exploring `MathConverse` that – as expected with volunteer-based surveys – the data can be noisy. For instance, despite informing participants, some did not fully understand that the model was not aware of the problem statement unless the user entered relevant info into the chat window.

[*] We faced a tradeoff between user tracking for data correlation and privacy. It is possible that a unique participant number that can only be used to correlate participant sessions but does not personally identify participants could be employed in the future without compromising the integrity of our data protection and privacy objectives. Informed consent would be critical, though it is not clear how the perception of tracking, no matter how clearly users are informed of its benign nature, would affect the willingness of users to participate or interact freely with the models. We chose to maximise privacy, participation and freeness of interaction in this study by not tracking participants at all.

On average, we find that our volunteers evaluate 3.1 problems (± 2.2 problems corresponds to one standard deviation) before stopping; this corresponds to typically just one round of going through each model and providing final preferences. Participants were not informed which model they are evaluating at any time. For any given model, participants interacted for an average of 3.4 queries (± 2.4; maximum 12 interactions taken).

Language Model Prompting. We include further details on the prompts used for each language model, and the way in which we call the associated API. The model IDs were as follows: "text-davinci-003" (InstructGPT), "gpt-3.5-turbo" (ChatGPT), and "gpt-4" (GPT-4). Sampling temperature was set to 0.0, and the maximum number of tokens permitted to generate was 512. Note, language models were never given the mathematics problem directly; we leave the choice of whether to provide the question to the language model, and how to typeset the question, as one for the user.

InstructGPT We interact with Instruct-GPT through the Completion API call. Our instruction prompt starts with "Help a professional mathematician solve a problem:".

ChatGPT and GPT-4 We query both models with the ChatCompletion interface. The system was setup with the instruction, "You are an assistant to a professional mathematician." Further generations were elicited through the user-assistant paradigm.

Further Details on Tasks Chosen. The ProofWiki problems were chosen to be self-contained and relatively brief. They consist of a mixture of well-known theorems and less famous, exercise-type problems, to provide a spectrum of different problems. The six topics are what typical undergraduate mathematical curricula cover.

Example Interface Screens. We also include example screenshots of our interface in Figures S2 and S3.

Additional Details on Taxonomy Construction

We include additional details on our taxonomy annotation scheme. The 256 human queries were split into 5 batches of approximately 50 queries each. Four authors participated in the taxonomy annotation; three authors categorised approximated 50 queries each, and one author annotated the remaining approximately 100 queries. Annotators received a spreadsheet containing the human query per interaction, as well as the original problem-of-interest, the model's response for that interaction step, as well as all prior interactions from between that participant and the AI system on that problem. As noted in the Methods, confusions were discussed amongst annotators. During the process, a new bucket was added – "Asking how to do a step of the proof" – after an annotator noted that some participants seemed to decompose the problem into a single step, rather than asking for help over the full proof. One annotator who had already categorised their 50 queries went back and reconsidered them with this new bucket in mind.

We include the annotation guidelines provided to the authors who agreed to partake in the taxonomy annotation:

```
Please classify the user query/interaction (in the "user_query") column into one or more of the
annotation buckets.
If a query falls under a bucket, please add a "y" for "yes" to that column. If you are unsure, but
think it may, please mark "m" in that column (for maybe). By default, we assume that a query does not fall
into a bucket. A query may fall into more than one bucket; though we expect that most fall into only one.
If you think that a query falls into an abstract category that is not represented, please write in the
"Other" column.
If you need to look at the original problem statement, see the "problem_declaration" column.
The previous human-AI interactions, with the humans' ratings for the AI outputs, are in
"previous_interactions".
We expect that annotating 50 user queries will take approximately 15 minutes. If it's taking
significantly longer, please let us know! We appreciate any time you may have to annotate even a few of
these!
Note: we will release the annotations later (without names of the annotator). If you aren't
comfortable with this, please do not proceed.
```

Additional Survey Observations

We include additional details and analyses into the data we collect and release in `MathConverse`.

Potential Misleading Generations under Human Uncertainty. We include additional examples wherein participants who were uncertain of their abilities to solve the problem on their own (see *Main Text*) rated a generation as completely mathematically correct when in fact it was not. These examples further illuminate the need to heed caution when leveraging these models at present for assistance, particularly in the case where the user may not be able to validate correctness. Below each example, we include commentary from mathematics expert, William Hart, from our author team.

If f is a polynomial in one variable of degree n over $\mathbb{Z}_p$, can f have $n+1$ roots?

No, a polynomial f of degree n over $\mathbb{Z}_p$ can have at most n distinct roots in $\mathbb{Z}_p$. This is a consequence of the fact that $\mathbb{Z}_p$ is a field, and hence a polynomial of degree n can have at most n roots in any extension field. To see why this is true, suppose that f has $n+1$ distinct roots $a_1, a_2, \ldots, a_{n+1}$ in $\mathbb{Z}_p$. Then the polynomial $g(x) = (x - a_1)(x - a_2) \cdots (x - a_{n+1})$ is also a polynomial of degree n over $\mathbb{Z}_p$. However, $g(x)$ is identically zero on $\mathbb{Z}_p$ since it has $n+1$ distinct roots. This implies that $f(x)$ and $g(x)$ are identical polynomials in $\mathbb{Z}_p[x]$, which contradicts the assumption that f has degree n. Therefore, f can have at most n distinct roots in $\mathbb{Z}_p$.

Commentary: The model's statement about $g(x)$ having degree n is not correct; it clearly has degree $n+1$. A degree n polynomial cannot be identically zero, so the next statement is also not correct. I don't follow its argument that f and g should be identical polynomials.

Can you show that $1 + a^n = 2^m$ has no nontrivial (i.e. n, $m > 1$) solutions in natural numbers?

Yes, I can help you prove that the equation $1 + a^n = 2^m$ has no nontrivial solutions in natural numbers. Proof by contradiction: Assume that there exist nontrivial solutions in natural numbers for $n, m > 1$. Let's analyze the equation modulo 2. $1 + a^n \equiv 2^m (mod 2)$ Since 2^m is always even, the left side of the equation must also be even. This means that a^n must be odd. Therefore, a must be odd as well, since an even number raised to any power is always even. Now let's analyze the equation modulo 4. Since a is odd, we can write $a = 2k + 1$ for some integer k. Then: $a^n = (2k+1)^n$ By the binomial theorem, we can expand this expression: $(2k+1)^n = \sum_{i=0}^{n} [C(n,i) * (2k)^i]$ 4Notice that all terms in the sum with $i > 0$ are divisible by 2^i, and thus by 4, except for the first term: $C(n,0) * (2k)^0 = 1$ So, modulo 4, we have: $a^n \equiv 1 (\mathrm{mod}\, 4)$ Now, let's look at the right side of the equation, 2^m. Since $m > 1$, we have: $2^m \equiv 0 (\mathrm{mod}\, 4)$ Thus, we have: $1 + a^n \equiv 1 + 1 (\mathrm{mod}\, 4)$ $2 \equiv 2^m (\mathrm{mod}\, 4)$ But this is a contradiction, since 2^m is always divisible by 4 when $m > 1$, and 2 is not divisible by 4. Therefore, our assumption that there exist nontrivial solutions in natural numbers for $n, m > 1$ must be false. Hence, the equation $1 + a^n = 2^m$ has no nontrivial solutions in natural numbers.

Commentary: It is obvious only that all the terms with $i > 1$ are divisible by 4. Therefore the generation is incorrect unless n is even.

Can "Hermitian matrix" be interpreted in the context of abstract linear algebra?

Yes, a Hermitian matrix can be interpreted in the context of abstract linear algebra. In abstract linear algebra, a Hermitian matrix is a matrix that is self-adjoint, meaning that it is equal to its own adjoint. This means that the matrix is equal to its own transpose, and the elements on the main diagonal are real numbers and the elements off the main diagonal are complex conjugates of each other.

Commentary: No, the matrix is not necessarily its own transpose. That is incorrect. The rest is poorly explained.

Ties in Rank-Order Preferences. As noted in the *Main Text*, some participants ranked models with ties (which occurred in 5 of all 15 complete preference ratings). In Table S1, we show the full ratings provided by participants in the presence of ties.

Relationship Between Web Occurrences and Ratings. While investigating the data, we wondered whether the models' performance, as measured via the correctness and helpfulness ratings, may be related to the frequency by which the base ProofWiki problem appeared in the training set. While we do not have a transparent measure of dataset prevalence, given these models are largely trained on text from the web (55, 56), we used the number of Google search queries associated with the problem name[†] as a proxy (see Figure S7). Intriguingly, we do not observe a reliable correlation between search term and average procured correctness and helpfulness (i.e., the Pearson correlation is not statistically significant for any model-rating pairing, and never more than 0.2). However, we encourage future work to further explore the relationship between prevalence of related problems in a models' training datasets and the models' provided assistance quality.

Additional Investigations into the `MathConverse` Annotated Taxonomy. We include additional analyses into our annotated taxonomy; in particular, we consider whether users' perceived helpfulness and correctness of the models' responses varies depending on the underlying query type. Such a question is important for both designers of AI systems – e.g., to tailor model updates which make the system more usable on queries a user may desire – as well as users themselves, who can better learn what kinds of queries the model is best equipped, at present, to provide useful responses for. Indeed, we find in Figure S6 that the models' quality of responses do vary as a function of query type. For instance, the models are good at providing definitions (as we noted in the *Main Text*), and struggle to uptake corrections.

[†] These names can be found in the released data.

Handling Errors in Task Specification. Additionally, after launching the survey, we were informed that two problem statements were incorrect; some assumptions or constraints that are crucial for the correctness of the theorems were missing or too strict. The first incorrectly stated theorem is Hurwitz's. Our description of it is as follows:

> **Hurwitz's theorem*** Let ξ be an irrational number. Then show there are infinitely many relatively prime integers $p, q \in \mathbb{N}_{>0}$ such that:
>
> $$\left|\xi - \frac{p}{q}\right| < \frac{1}{\sqrt{5}q^2}$$

The relatively prime integers p and q should be allowed to be any integers instead of positive integers. This is due to a copy error when we reformatted the original ProofWiki statement[‡].

The second such statement is Tamref's Last Theorem:

> **Tamref's Last Theorem*** The Diophantine Equation:
>
> $$n^x + n^y = n^z$$
>
> has exactly one form of solutions in integers:
>
> $$2^x + 2^x = 2^{x+1}$$
>
> for all $x \in \mathbb{Z}$.

The theorem was not fully correct as stated on ProofWiki[§] the day we accessed the content (2 April 2023), as it did not ask for *non-trivial* solutions. The solution $n = 0$ trivially satisfies the Diophantine Equation.

We opted to leave these problems in the survey to act as "canaries" – permitting us to detect whether participants (or models) were able to identify that problems were incorrectly stated in the written form. We find that since these two theorems are relatively well-known or easily understood, our experienced mathematician participants tended to use the definitions they had remembered to override the conditions given in the statements: the incorrect or missing assumptions are amended or added mentally by the users. We find no significant difference in the user behaviours when they deal with these two problems. Sometimes, human users simply state the names of theorems or express them in natural language (e.g., saying "Weak Law of Large Numbers" instead of its full statement), thus ending up not propagating the errors to the LLMs.

For Tamref's Last Theorem, only GPT-4 made reasonable attempts in our collected responses. When prompted with the incorrect statements, GPT-4 seemed to be aware of the subtlety due to the lack of a non-triviality assumption: it either pointed out that there is a solution at $n = 0$ or explicitly said that it was looking for non-trivial solutions. For Hurwitz's Theorem, all LLMs follow the incorrect assumption of the positivity of p and q and fail to come up with good solutions, if they were given the incorrect problem statement.

Interaction Ratings by Mathematics Topic. Recall, participants selected a mathematics topic at the start of the survey and interacted with the models on problems from that topic. We decompose the performance of models by participant topic selection in Figure S5. We depict average correctness and perceived helpfulness ratings for interactions on problems in each topic. Due to limited number of problems seen within each topic, we cannot draw definitive conclusions about differential model performance across these topics; however, we include these analyses for completeness and encourage future work to scale the array of problems considered to better characterise how a model's assistive capabilities varies by area of mathematics.

Post-Survey Testimonials from Participants

We include additional quotes sourced from participants after completing our survey. Participants were reached out to via connections from the authors. We first include the complete question asked in the testimonial, followed by responses received. It is worth noting that testimonials were curated approximately one month after the interaction; we would encourage future work to consider such a survey immediately after completion of a `CheckMate` interactive session.

Why Stop Interacting?. *Full question: "Why did you stop interacting?"*

- `"I usually stopped when I had a distinct impression that improvement wasn't likely past the current point. That could be very early on, for example after a very poor initial response that demonstrated a profound mathematical shortcoming, or it could be after a longer interaction that convinced me no further improvement of the model's understanding or ability to accept and correct for its mistakes was likely with further prompting"`

- `"In the first experiment, I just tried to be fair, and give each model three queries (also because I thought I could just solve it). In later experiments, simply when it didn't seem productive anymore, or I felt there was nothing more to ask."`

‡ https://proofwiki.org/wiki/Hurwitz%27s_Theorem_(Number_Theory)
§ https://proofwiki.org/wiki/Tamref%27s_Last_Theorem

- "I feel like I have already gained enough ideas of the model's capability. My daily work with mathematical proofs involves more complex manipulations of mathematical objects and interactions with proof assistant, which is still beyond the powers of the existing GPT4 model."

- "Usually the model either gave a good answer after a few prompts or didn't seem to be able to give one. Sometimes it happened that I did not get any better explanation to additional questions at which point I stopped."

Human Uncertainty in Model Correctness?. *Full question: "Were there times where you didn't know whether the model's generation was correct? If so, how did you handle this?"*

- "Yes. I did look up some facts and proofs. The main reason was that if the study is to be scientific it should not rely on my incorrect assertions."

- "Of course, whenever I asked about a definition, or about a theorem I didn't know (I tried not to look much into external resources). First, I was also often too lazy to check the model's lengthy response (when it was a proof, I could be punctual with definitions, there is no way to be sure). But even if I check the proof and find a bug there, it doesn't mean that the theorem doesn't hold... Once the model really confused me, when I was solving some problem about rational approximation (I don't remember it exactly), the model suggested continued fractions which sounded reasonable but the standard error estimate for continued fractions was too weak. So I asked whether there exists a closer estimate, and the model provided me one, and just in the final review of the exchange, I figured out that it was false."

- "Yes, my biggest concern with LLMs is the uncertainty of the correctness of the generated answers. One way is, of course, to use the generated answer as a hint and then search with more reliable sources (e.g., textbook and peer-reviewed papers). An alternative way is to keep asking further questions and the same question from a different angle (like having a conversation with a real person). With rounds of Q&A, I can then gain a better sense of the original question."

- "Most times it was clear, however, sometimes the generations became quite lengthy and therefore difficult to check (especially if there was a mistake at the beginning). I found it similar to grading student homework which is easy when everything is correct (or false), but more difficult when explanations seem to contain valid arguments while still not being entirely correct. Study design feedback: I would have liked to make full text remarks after interacting with a model to be able to judge this more appropriately."

Correct, but Unhelpful?. *Full question: "Were there instances where a model's response was mathematically correct, but you found it unhelpful? If so, why was it unhelpful?"*

- "Not really, however some of the models state facts that they know on the given topic instead of answering the questions that were asked. This could be helpful in certain situations, but the models don't seem to discern when this is. It can be unhelpful if the answer to the actual question then gets cut off."

- "Not much but I remember that there was a problem about B-algebras (I think it was called that way), and proofwiki has a very atypical notion of a B-algebra. I didn't know what a B-algebra is, so I asked the model, and he told me some definition of a B-algebra that was likely mostly correct but it didn't fit the problem at all. Eventually, this was one of the cases where I just had to go to proofwiki to figure out what the problem means since neither I nor the model had an idea."

- "Yes, there is. The model is really good at retrieving mathematical definitions/statements that I cannot remember exactly. However, the model is yet good at proposing non-trivial proof strategies given those definitions - they can give some sound but not very helpful proof suggestions."

- "It would have been very helpful if answers had been presented more clearly (rendered formulae instead of latex code, more display formulae), but this is not due to the model. Sometimes I tried asking for a hint, which didn't produce good output. Therefore, I asked for a step-by-step solution which was then more helpful, however, this takes away the opportunity (for a student work with such a model) to actually also think about the question by themselves. "

Using LLM-Based Assistants in Practice?. *Full question: "Are there circumstances in which you would use one of the models you interacted with to assist you in mathematics? (or would have used in a particular way earlier in education, if available)"*

- "I'm going to try and use GPT-4, however I believe I will have to be very creative to do so. There's no sense in which one can just ask GPT-4 questions and expect reliable, carefully thought out or ingenious responses. For me it is an open question whether it can be genuinely helpful. I've already wasted a lot of time chasing up things GPT3/4 hallucinated."
- "With the high uncertainty about when the answer is true, I don't think it is usable in practice yet. By the way, I just tried another free language model (HuggingChat) about whether there is a reference to a particular theorem which I thought should be known. It gave me three references, and none of them was correct... I don't expect the models from the experiment to behave much better.¶"
- "I would definitely use them for initial pilot study (e.g., literature review, identifying potential proof routes and related well-known theorems). Nevertheless, given the unreliable nature of LLMs, I may always double-check the answers. Another big usage of LLMs is to polish my writings (even in mathematical writings), which is super helpful for a non-native speaker."
- "I'd consult such models mainly to check for preexisting, relatively well-known results that I might not know about as a complement to classical literature search. And as a tool to improve my writing of non-technical portions of mathematical texts. The current models are definitely to poor in logical reasoning to be employed for finding, checking or rewriting proofs. I'd be quite afraid to develop incorrect understandings by using a model which can present wrong arguments in a very convincing way."

Interactive Case Studies with Experts

We now include a complete listing of the case studies. A complete transcript of interactions for each case study example is included in *SI Dataset 3*. We maintain the original text wording from each case study's author for authenticity, with only minor edits for precision and coherence. As noted in the *Main Text*, each author provides general remarks as well; we do not re-include those here. We do keep all problems that the case study authors included (though one may notice that some are also included in the *Main Text*) to avoid breaking flow.

A Deep Dive into ProofWiki First, our recruited experts conduct a deeper dive into some of the problems we explored in our previous evaluation. Specifically, we use the problems as a playground to explore how much the model seems to "know" about relevant concepts and further characterise what interactions can yield better (or worse) performance and assistance experience. We focus on GPT-4 (in chat mode) because it has the strongest overall performance in the study above. The experts chose to refer to GPT-4 "*the assistant*" in the rest of this section.

The first batch of case studies are provided by Dr. William Hart, a number theorist by training; the second were primarily contributed by Dr. Wenda Li, a formal mathematics expert‖.

Number Theory Evaluation. *Contributed by William Hart*

We provide an in-depth analysis of a number of GPT-4 responses to number theoretical questions. Number theory is an area of mathematics where problems are often simply stated, but difficult to solve, involving arbitrarily deep mathematics in their solution. Whilst we didn't interact with the model to work on any problem requiring very deep methods for its solution, such as Fermat's Last Theorem famously proved by Andrew Wiles, we did have a chance to observe the model as it struggled with problems ranging from trivial to moderately challenging.

> **ProofWiki problem 21** Show that the equation
>
> $$1 + a^n = 2^m$$
>
> has no solutions in the integers for $n, m > 1$.

Problem 21 is a simple Diophantine equation. The problem is quite obscure (a verbatim Google search gives 10 results) and thus not likely to appear in training material repeatedly. The model took very reasonable steps towards solving the problem: it started by claiming the proof is by contradiction and proceeded to reason about the assumed solution for $n, m > 1$.

It begins well by reasoning that a must be odd because $1 + a^n$ is even. No explanation is given for this, but an experienced human wouldn't explain this step either given the routine nature of parity arguments in Number Theory.

The next step is to take an expression $(2k + 1)^n$ which has appeared and expand it using the binomial theorem. However, it does this in a surprising way, splitting the resulting sum into the first two terms and then a sum for the remaining terms.

$$(2k+1)^n = \sum_{i=0}^{n} \binom{n}{i} (2k)^i = 1 + n(2k) + \sum_{i=2}^{n} \binom{n}{i} (2k)^i$$

¶ This response was provided to us on May 19, 2023. It is feasible that HuggingChat has or will improve since then.

‖ Case studies were conducted between April and early May 2023.

This is impressive because GPT-4 is exhibiting some planning. It clearly has in mind to work modulo 4 and it can see that all of the terms of the final sum might vanish modulo 4. Indeed this is the very next claim that it makes.

Whilst it didn't explain why every term of the final sum is divisible by 4 it was asked on subsequent generations to explain this step and it correctly did so.

However, things do not go so well from here. It now claims that we can write the original equation $1 + a^n = 2^m$ as

$$1 + 2kn + 4s = 2^m$$

for some s. This is a beguiling step that a human might overlook as correct, but it is not. The expression $1 + 2kn + 4s$ is the expression for a^n not $1 + a^n$. GPT-4 has made an algebraic error. This sort of thing is unfortunately very common and lets GPT-4 down on many examples.

Asking GPT-4 to self correct did not help it notice and correct its mistake. To see if it could eventually produce a completely correct proof, it was asked numerous times to solve the problem. Whilst its overall strategy was good on each generation, different algebraic mistakes occurred each time so that a correct proof was not eventually reached.

ProofWiki problem 28 Show that

$$3 = \sqrt{1 + 2\sqrt{1 + 3\sqrt{1 + \cdots}}}.$$

Problem 28 is a more difficult problem and the model is completely unable to deal with it. It admits that problems involving nested radicals can be difficult and actually gives up after standard methods don't make any headway.

A consistent problem here is an inability to write down a correct expression for a recursive relation to describe the nested radical. GPT-4 seems to be convinced that the expression under each square root is the same, so that if we write the initial expression $3 = \sqrt{A}$ then we also have $3 = \sqrt{1 + 2\sqrt{A}}$ and $3 = \sqrt{1 + 2\sqrt{1 + 3\sqrt{A}}}$, etc**.

On subsequent attempts additional terms of the initial sequence were provided in the hope that it would pick up on the increasing sequence of constants that the square roots are multiplied by.

Whilst GPT-4 would confirm that it had noticed this pattern, it would always proceed ignoring this fact. On each generation, GPT-4 would finish off by noting it got the wrong answer and that this must be because it didn't take this increasing sequence of constants into account! It's as though GPT-4 only knows one way to handle nested radicals, and knowing that this won't work here, tries it anyway, inevitably getting the wrong answer.

To probe a little deeper, GPT-4 was instead prompted in a direction that might allow it to make partial progress. The hint was given to try peeling the expression on the right hand side one square root at a time, working backwards from the desired result that the full nested radical should have the value 3 to see if some pattern could be found in the values of the inner nested radicals.

It was easy to prompt it so that it heads in that direction but on every generation it made hopeless algebraic and numerical errors, once again illustrating that very often what holds it back is high school algebra rather than the depth of the mathematics.

As GPT-4 could not be coaxed into returning correct values for the sequence of inner nested radicals, the attempt to solve the problem using GPT-4 was abandoned.

ProofWiki problem 24 Let ξ be an irrational number. Then show there are infinitely many relatively prime integers $p, q \in \mathbb{N}_{>0}$ such that:

$$\left|\xi - \frac{p}{q}\right| < \frac{1}{\sqrt{5}q^2}$$

Finally, Problem 24 is another difficult problem. Its solution on the ProofWiki website requires a number of lemmas and some subtle reasoning. Solving a problem of this kind would require some planning capability, or at the very least the ability to backtrack and experiment with various ideas. This is something that GPT-4 doesn't appear to possess beyond what can be 'computed' within the model itself.

GPT-4 does make the completely reasonable first step of approaching this problem using a continued fraction expansion of the irrational number ξ. Many approximation problems of this kind do indeed proceed this way. Continued fractions yield a sequence of convergents p_n/q_n that converge to the irrational number ξ.

After picking a reasonable theorem from the theory of continued fractions and applying it, GPT-4 has the following expression

$$q_n q_{n+1} > \sqrt{5}q_n^2.$$

At this point it is clear that GPT-4 does not know how to proceed, but knows what it should end up with, so makes the unsubstantiated claim that this inequality is satisfied when $q_{n+1} > \sqrt{5}q_n$.

There is no reason to infer that this should be the case at this point in the problem and if the particular chosen approach is to work out, this would have to be proved. Instead of doing so, GPT-4 just asserts that it is true without attempting to prove it.

** Also highlighted in *Main Text, section Per-Expert Conclusions.*

When asked directly how to prove this statement GPT-4 clearly has no idea how to do so and makes a completely bogus claim that a sequence with linear growth will eventually outgrow a sequence with exponential growth. It seems to be common for GPT-4 to hallucinate details when things aren't working out or if it doesn't know a reasonable answer.

In other contexts we have observed that GPT-4 can produce better output if asked to stop itself if a particular mathematical approach does not seem to be working out and to try another approach. When prompted to do so in this particular case GPT-4 did indeed try numerous reasonable strategies but unfortunately it was still not ultimately successful. This was in part due to poor choices along the way and partially due to being plagued by algebraic errors which ended up misleading it.

For balance we mention that the failed attempts above were not entirely characteristic of GPT-4 which can in some cases produce perfect answers.

For example, Problem 27 is solved completely, although slightly inefficiently and Problem 23 is correct except for a single bogus explanation which was not particularly significant. However, it should be pointed out that Problem 27 is quite trivial, essentially requiring only the binomial theorem and Problem 23 is completely standard in many texts on elementary Number Theory. It is very unlikely that the standard proof would be hit upon at random, and the fact that GPT-4 generates it perfectly is probably an indication of the relative abundance of proofs in training material.

Problem Perturbation to Probe Memorisation. *Contributed by Wenda Li*

If a system simply memorises the answers to problems, its performance can greatly differ depending on whether the problems it is evaluated on are memorised. In this section, we evaluate GPT4's performance on variations of problems from ProofWiki, which are far less likely to be appear in training data since we make novel variations. Concretely, we varied the problems in three different ways to test the model's understanding of the problems: asking for definitions of concepts, loosening assumptions of problems, and instantiating abstract variables with values. Two problem instances and their variations are presented due to them being the most interesting and revealing examples from all that were tried.

> **ProofWiki problem 25** Let $a, b \in \mathbb{N}_{>0}$ such that there exists no $m, n \in \mathbb{N}_{>0}$ such that $a^m = b^n$. Prove that $\log_b a$ is irrational.

Presented the problem statement above, the assistant gave a perfect answer with step-by-step calculations. To test if the assistant has a true mathematical understanding of the problem, we first asked for definitions of concepts used, and then varied the original problem by loosening some of the assumptions made, and asked the assistant for a proof in the new setting.

Asking for definitions We found that the assistant gave the correct definitions in the theorem statement as well as in its own proof. Concretely, it gave the right answers for: the definition of logarithm; the range of a logarithm's base; the meaning of the set $\mathbb{N}_{>0}$; and whether $\log_b a = \frac{p}{q}$ can be a negative number (p and q are variables arising from the assistant's own proof).

Loosening assumptions We started by asking the assistant *whether the proof still holds if we instead have $a, b \in \mathbb{R}_{>0}$?* The assistant understood the meaning of $\mathbb{R}_{>0}$ and confirmed the derivation still held, so the original lemma/proposition has been generalised (since one of its assumption has been relaxed). Later, we attempted to generalise the proposition further by dropping the assumption $a \in \mathbb{R}_{>0}$ or $b \in \mathbb{R}_{>0}$:

We continued by asking *if dropping the assumption that $b \in \mathbb{R}_{>0}$ or $a \in \mathbb{R}_{>0}$ affects the original proof?* The assistant knew that these assumptions were necessary to make the log function well-defined, and pointed out that dropping either of the assumptions would invalidate our previous derivation.

These variations, though not impossible, are unlikely to appear together with the problem in the training data of the assistant. We think the assistant does have some understanding of the underlying mathematical concepts and its own proof, in the context of this problem.

> **ProofWiki problem 39** Let X be a random variable. Assume $\mathsf{E}(X) = \mu$ for some $\mu \in \mathbb{R}$ and $\mathsf{var}(X) = \sigma^2$ for some $\sigma^2 \in \mathbb{R}_{>0}$. Show that for all $k > 0$: $\Pr\left(|X - \mu| \geq k\sigma\right) \leq \dfrac{1}{k^2}$.

Given this problem statement, the assistant mentioned that we can use the Chebyshev's inequality, and then re-stated the problem in an almost identical way but with different variable names. This demonstrates a certain level of variable unification, which is an important concept in automatic theorem proving[††].

Variable instantiation We then checked whether the assistant knew how to instantiate variables by asking it whether the proof still holds when the following concrete values are assigned to k: 2, $\sqrt{2}$, $\sqrt{2}-1$, $\sqrt{2}-2$, and $(\sqrt{2}-2)^2$. Human inspection finds the assistant's behaviour to be correct. The assistant can clearly handle concrete calculations even when k is a relatively complicated number (e.g., $\sqrt{2}-1$). The model also knows that the previous derivation cannot be carried out when $k = \sqrt{2}-2$, a negative number[‡‡].

An interesting observation arose when the assistant was not confident of its derivations: we asked: "*are you sure $(\sqrt{2}-2)^2 > 0$?*" The answer should be affirmative, but the assistant started to apologise and revise its previous correct calculation by saying "*When $k = (\sqrt{2}-2)^2$, the value of k is indeed non-negative, but it is actually equal to 0, not greater than 0.*" When we asked again "*Why do you say your previous statement was incorrect and $k = 0$? I don't understand.*", the assistant corrected its previous mistake and produced the right evaluation.

[††] Also highlighted in *Main Text, section Per-Expert Conclusions.*
[‡‡] Also highlighted in *Main Text, section Per-Expert Conclusions.*

We found that the assistant is generally quite capable with variable instantiations and evaluating certain complex expressions, with the occasional mistake made with low confidence. We hypothesise that the mistake may be a defect of its reinforcement learning with human feedback (RLHF)(57–59) training: the human feedback is mostly assumed to be right, and when the feedback questions a true fact, the assistant concurs and alters its own (correct) response.

Investigating the Boundary between Easy and Hard Problems. *Contributed by Timothy Gowers*

If we want to understand how and to what extent large language models can help mathematicians, it is clearly important to understand what they can and cannot do. A range of views have been expressed on this topic, with some saying that they already show glimmerings of AGI (32), and others dismissing them as mere "stochastic parrots" (60). On the latter view, the successes that LLMs undoubtedly have solving mathematical problems are to be understood as very good guesses of what the outward form of a proof would look like, unaccompanied by any genuine understanding of what that proof means, even when it is correct.

A difficulty with evaluating the level of understanding of a language model is its opacity: for any particular answer it gives, we do not know the extent to which it is merely an amalgam of texts it has seen as part of its training data and the extent to which it has actually had to build its answer in a coherent way. One way to try to get round this problem is to ask the LLM questions that are deliberately designed to be "quirky" and non-standard. A good source of such questions is ones that ask whether mathematical objects of certain kinds exist with various artificial combinations of properties.

Timothy Gowers, a mathematician from our author team, tested GPT-4 on many such questions, attempting to identify a "boundary" between what it could and could not do. This imposed a further constraint on the questions: that they should ideally be modifiable in various ways, so that one can "turn the dial" until GPT-4's performance drops.

Example 1 Here is a prompt given to GPT-4.

> Can you find an example of a set A of positive integers that satisfies the following three conditions? (The LaTeX is compiled here but was uncompiled in the prompt.)
>
> 1. $|A| = \min(A)$.
> 2. A consists of prime numbers only.
> 3. A has cardinality at least 5.

The response was the following correct and concise answer.

> Yes, here's an example of a set A of positive integers that satisfies the given conditions:
>
> A = 5, 11, 17, 23, 29
>
> The cardinality of A is 5, which is also the minimum element in A.
>
> 1. All elements in A are prime numbers.
> 2. The cardinality of A is 5, which is at least 5.
> 3. Therefore, set A = 5, 11, 17, 23, 29 meets all three conditions.

Despite the fact that this answer is correct, it has a revealing feature. Why, one might ask, does GPT-4 not give the more obvious answer $\{5, 7, 11, 13, 17\}$? That is, why does it not take the five smallest prime numbers greater than or equal to 5?

A clue lies in the fact that the set it does choose, $\{5, 11, 17, 23, 29\}$ has the additional interesting property that it forms an arithmetic progression. This property has led over the years to the set being mentioned in many articles about arithmetic progressions that consist of primes, and presumably a significant fraction of those also mention that the number of elements is 5.

What is illustrated here is a preference by GPT-4 to use off-the-shelf examples, a preference that is strong enough that it will often do so even when the examples do not in fact have the required properties.

Why would most human mathematicians prefer to take the first five primes, starting at 5? It is because that example is easily generalisable: if we change the number 5 to any other number n, we simply have to choose a prime p that is at least as big as n and take the first p prime numbers from p onward.

To understand GPT-4's behaviour better, it is therefore natural to give it the following slightly modified prompt.

> Can you find an example of a set A of positive integers that satisfies the following three conditions?
>
> 1. $|A| = \min(A)$.
> 2. A consists of prime numbers only.
> 3. A has cardinality at least 6.

The response was

Yes, here is an example of a set A of positive integers that satisfies the given conditions:

A = 7, 11, 13, 17, 19, 23

1. |A| = 6, and the minimum element in A is 7, so |A| = min(A).

2. All elements in A are prime numbers: 7, 11, 13, 17, 19, and 23. 3. A has cardinality 6, which is at least 6.

The set A satisfies all three conditions.

Even after its error was pointed out, and a few more rounds of the conversation had taken place, GPT-4 did not manage to eliminate all mistakes and give a correct answer. Indeed, when told that 6 does not equal 7, it at first tried to explain why no such set could exist.

This example lends some weight to the stochastic-parrot view: the problem is extremely easy for a human, but it is also a sufficiently artificial question that GPT-4 will not be likely to have encountered it, or anything very like it, in the texts on which it has been trained. One might argue that it does at least give a set of consecutive primes this time, but that again is what one would expect, given that sets of consecutive primes will appear much more frequently in the literature than other sets (a tendency that one might guess is overridden when the sets start with 5 and have size 5).

Example 2 This was a successful attempt to trick GPT-4. It was first asked to evaluate the integral $\int_{-1}^{1} x^3 \cos x \, dx$. It did so by a laborious argument using iterated integration by parts, presumably because the integrand is typical of the integrands in integration-by-parts exercises. After getting everything right until close to the end, it made an elementary mistake, but with a bit of help it arrived at the correct answer, 0 (though still persisting in some of its incorrect calculations). On being asked whether there was a shorter argument, it gave the right response: that the integrand is an odd function and the integral symmetric about 0, so the integral is 0.

Then it was asked to evaluate the integral $\int_{-1}^{1}(x^2 - 1/3) \, dx$. Again it did so correctly, obtaining the answer 0. On being asked whether there was a simple reason for this, it once again pointed out that the integrand was an odd function, and even supplied a bogus proof that the function is odd.

This behaviour again fits the stochastic-parrot hypothesis quite well: almost always if one is asked for a simple reason that the integral of a function over a symmetric interval is zero, the correct response is that the function is odd. Whether or not it actually is odd is for GPT-4 a secondary consideration.

Example 3 A nice problem that mathematics undergraduates tend to find quite hard is to determine whether there is an order-preserving bijection between the rational numbers and the dyadic rationals. Surprisingly, the answer is yes, and the proof is by what is known as a back-and-forth argument. That is, one enumerates the rationals and the dyadic rationals, and then one alternates between choosing a match for the first unmatched rational and the first unmatched dyadic rational, making sure at each stage that the order is preserved.

When GPT-4 was asked the problem, it tried to prove that no such bijection could exist. Each time its mistakes were pointed out to it, it replaced its bogus argument by a slightly modified bogus argument.

Upon being asked whether it knew about back-and-forth arguments, it said that it did, and explained that they could be used to prove a theorem of Cantor, that any two countable dense subsets of the real numbers are order isomorphic. It did not seem to realise that the problem it had been asked was a special case of this theorem. It also tried to explain why one could not use a back-and-forth argument to prove that the rationals and the dyadic rationals are order isomorphic, but after its explanation was criticised, it proceed to give a convincing sketch of how such an argument would indeed work.

Example 4 When GPT-4 was asked whether there is a positive integer n such that $n + k$ is divisible by k for every integer k in the range $\{1, 2, \ldots, 100\}$, it said no, and offered bogus proofs. After being steered towards a positive answer via certain easier questions, it suggested taking n to be $\mathrm{LCM}(1, 2, \ldots, k) - 1$. This was interesting because $\mathrm{LCM}(1, 2, \ldots, k)$ would have been a correct answer, but the pointless subtraction of 1 ruined it. Furthermore, it gave an argument that would have been correct if the -1 had not been present.

What might explain this act of self sabotage? One idea is that GPT-4 is influenced by Euclid's proof that there are infinitely many primes, which assumes that $p_1, \ldots, p_k$ are all the primes and considers the number $p_1 p_2 \ldots p_k + 1$. An alternative argument would be to consider the number $\mathrm{LCM}(1, 2, \ldots, k) - 1$, where k is at least as large as the largest prime.

However, this explanation is rather speculative, and a Google search does not seem to back it up. When GPT-4 was asked why it had subtracted 1, it did not provide a convincing reason either.

More revealing was its behaviour when its example was criticised on the grounds that, for instance, $\mathrm{LCM}(1, 2, \ldots, k) - 1$ is odd, and therefore not divisible by 2. Instead of adjusting its answer, as a human mathematician might, it decided that no such n existed, and when its arguments for that conclusion were criticised, it went back to the example of $\mathrm{LCM}(1, 2, \ldots, k) - 1$. Even when asked whether $\mathrm{LCM}(1, 2, \ldots, k)$ would work better, it initially said no. So this was not really a "boundary" example, and more just a problem on which GPT-4 got into a rut and could not get out of it.

Example 5 A better example of a "boundary" problem was the following question: does there exist a graph with eight vertices such that every vertex has degree 3? Once again GPT-4 demonstrated its liking for off-the-shelf examples, giving the example of the 3-dimensional discrete cube. (An alternative approach is to take eight vertices joined in a cycle, and to join each vertex in addition to the vertex opposite it in the cycle.)

When asked whether there was a graph with eight vertices such that every vertex has degree 5, it performed far worse. It did not know of any off-the-shelf examples, and was probably incapable of tricks such as taking the complement of an 8-cycle (which works because in the cycle every vertex has two neighbours, so in the complement of the cycle it has five neighbours). That is, it does not appear to be capable of taking an off-the-shelf example and *modifying* it in a suitable way. Instead, it resorted to listing the vertices as A, B, C, D, E, F, G and H, and for each vertex giving a list of its neighbours. The trouble is that this kind of approach gave it many opportunities to fail as a result of familiar weaknesses such as a propensity to make calculation errors or to write down inconsistent statements. For instance, over its several attempts it would frequently list a vertex v as a neighbour of another vertex w, but without listing w as a neighbour of v. Eventually, probably with a slice of luck, it came up with an example that turned out to be the complement of the disjoint union of a 3-cycle and a 5-cycle. (Since the complement has to be regular of degree 2, it will always be a disjoint union of cycles.)

Example 6 It has been noted that GPT-4 likes well-known patterns, and that one way of inducing it to fail is to ask it questions that will tempt it to give answers that fit those patterns. The following attempt to lead it astray in that way was a partial success. It was asked to find integers a and b such that the sequence $(1, 3, a, 7, 9, b, 13, 15)$ is strictly increasing but not an arithmetic progression. It responded by choosing $a = 5$ and $b = 11$, thereby falling headlong into the trap. However, it then did a check by calculating all the successive differences. On observing that it obtained the difference sequence $(2, 2, 2, 2, 2, 2, 2)$, it then modified its choice of b to 12, after which it recalculated the difference sequence, obtaining $(2, 2, 2, 2, 3, 1, 2)$ and declaring itself satisfied.

This was another example where despite arriving at the correct answer, GPT-4 argued in a very non-human way. The main non-human feature was of course that it began by making the one guess that it needed to avoid (out of the strictly increasing possibilities). However, the whole approach of guessing and then verifying is inappropriate for the problem, since it is much more efficient to reason as follows: first, we note that *if* the sequence is to be an arithmetic progression, then it will have to have common difference 2 (since the first two terms differ by 2) so it is sufficient to ensure that $a \neq 5$. This kind of forward planning appears to be beyond the current capabilities of GPT-4, (though maybe it could be induced to some small extent with careful prompt engineering).

We briefly mention its response to a variant of the problem, where it was asked whether it was possible to find integers a, b and c such that the sequence $(1, a, b, c, 14)$ is an arithmetic progression. It answered yes, then set d to be the common difference, obtained the equation $14 = 1 + 4d$, solved for d, discovered that d was not an integer, and answered no, having apparently forgotten that it had previously answered yes. This showed a reluctance to plan in advance even in a situation where it was entirely capable of carrying out the required planning.

Example 7 GPT-4 was asked to find a colouring of the set $\{1, 2, 3, 4, 5, 6, 7, 8, 9\}$ using three colours and satisfying the conditions that each colour is used three times, and no integer n has the same colour as $n + 1$ or $n + 3$. The obvious way to solve this problem is trial and error, which for a typical human will work with a small amount of backtracking. It did indeed choose this approach at first, but failed quite badly because it was unable to check the conditions properly, which caused it to assign colours that were forbidden by the conditions, and, in the other direction, to claim that certain choices were forced when they were not. After a couple of failed attempts, it switched to trying to find a "systematic" approach. One such attempt was to split the set into even and odd numbers, but that did not help it find a correct colouring. It even tried splitting the numbers into the three sets $\{1, 4, 7\}$, $\{2, 5, 8\}$ and $\{3, 6, 9\}$ and assigning one colour to each set, which violated the $n + 3$ condition many times over.

In sum, its ability to check simple conditions was too unreliable for it to be able to push through a trial-and-error approach, and for this problem a guess-and-check approach has a very low chance of success.

Example 8 A somewhat similar question on which it performed badly was to find a sequence of nine distinct integers with no increasing or decreasing subsequence of length 4. Here it once again showed its taste for patterns: the problem was that it did not choose appropriate patterns. An example that was typical of its output was $(4, 1, 5, 2, 6, 3, 7, 8, 9)$. Interestingly, when, after a few failures, it was given a reason-step-by-step prompt, it produced the same example, this time after talking about interleaving sequences – an idea which, if used correctly, leads to solutions such as $(1, 4, 7, 2, 5, 8, 3, 6, 9)$. However, encouraging it to use interleaving just led to further incorrect guesswork, an extreme example of which was when it interleaved the sequences $(1, 3, 5)$ and $(2, 4, 6, 7)$ to obtain $(1, 2, 3, 4, 5, 6, 7)$, stuck 8 and 9 on the end, and proposed $(1, 2, 3, 4, 5, 6, 7, 8, 9)$ as a solution (complete with a "check" that it worked).

When given the hint that it might like to start its sequence with $(7, 8, 9)$, it immediately made the obvious suggestion $(7, 8, 9, 4, 5, 6, 1, 2, 3)$. When asked for a rigorous proof that this sequence has the desired property, it gave an inadequate answer, stating correctly that the longest increasing subsequences are those that begin with 7, 4 and 1, and stating incorrectly that the longest decreasing subsequences start with 9, 6 and 3. There was nothing resembling the argument that a decreasing sequence can contain at most one term out of each of the subsequences $(7, 8, 9), (4, 5, 6)$ and $(1, 2, 3)$.

Example 9 The following is a question on which GPT-4's performance was more or less perfect. It was asked to evaluate the sum $\sum_{r=2}^{n} \log(1 - 1/r)$. It noticed that $1 - 1/r$ can be rewritten as $(r - 1)/r$, which therefore leads to a telescoping sum and and eventual answer of $\log(1/n)$. It did not give a detailed explanation of why, as it put it, the denominator in each term cancelled with the numerator in the succeeding term, but when asked for one it gave a good one (though it argued for a telescoping product inside the logarithm rather than a telescoping sum, which might have been simpler).

This was interesting because it required putting together two tricks: one to rewrite $1 - 1/r$ and the second to use the telescoping. However, its performance on a follow-up question strongly suggested that it had seen the question in its training

data. It was asked whether it could evaluate the sum $\sum_{m=1}^{n} \log(1/m(m+1))$. This question was intended to trick it, since the sum $\sum_{m=1}^{n} 1/m(m+1)$ is famously evaluated by rewriting $1/m(m+1)$ as $1/m - 1/(m+1)$ in order to obtain a telescoping sum. However, instead of falling into the intended trap, it stated falsely that it could rewrite the sum as $\sum_{m=1}^{n} \log((m+1)/m)$, after which it obtained a telescoping sum very similar to that of the previous question.

When asked a further follow-up question to test how well it understood the principle of telescoping sums, it gave an answer that had good points and bad points, both of which indicated that it was good at capturing the look of a correct answer while not operating at the level of the underlying concepts. The question was to determine the sum $\sum_{n=1}^{N} g(n)$, when $g(n)$ had been defined to be $f(n) - 2f(n+1) + f(n+2)$. It is instructive to look at its complete answer.

Let's work out the sum. We have

$$\sum_{n=1}^{N} g(n) = \sum_{n=1}^{N} (f(n) - 2f(n+1) + f(n+2))$$

We can break this into three separate sums:

$$\sum_{n=1}^{N} f(n) - 2\sum_{n=1}^{N} f(n+1) + \sum_{n=1}^{N} f(n+2)$$

Let's shift the indices in the second and third sums to make the limits match up with the first sum:

1. For the second sum, we substitute $m = n+1$, so $n = m-1$. When $n = 1$, $m = 2$; when $n = N$, $m = N+1$.
2. For the third sum, we substitute $m = n+2$, so $n = m-2$. When $n = 1$, $m = 3$; when $n = N$, $m = N+2$.

Doing this, we get:

$$\sum_{n=1}^{N} f(n) - 2\sum_{m=2}^{N+1} f(m) + \sum_{m=3}^{N+2} f(m)$$

Now, we can write out the sums explicitly to see what's happening:

1. The first sum is: $f(1) + f(2) + f(3) + \cdots + f(N)$.
2. The second sum is: $2f(2) + 2f(3) + \cdots + 2f(N+1)$.
3. The third sum is: $f(3) + f(4) + \cdots + f(N+2)$.

By looking at these sums, we can see that most of the terms will cancel out. For example, the term $f(2)$ is added in the first sum and subtracted twice in the second sum. Similarly, the term $f(3)$ is added in the first sum, subtracted twice in the second sum, and added once in the third sum.

After all the cancellations, we are left with:

$$= f(1) - 2f(2) + f(N) - 2f(N+1) + f(N+2)$$

So, the sum $\sum_{n=1}^{N} g(n)$ simplifies to $f(1) - 2f(2) + f(N) - 2f(N+1) + f(N+2)$.

A first comment is that the final answer here is not just wrong, but clearly wrong to an experienced mathematician, because there is a symmetry in the problem that guarantees that the number of leftover terms at the two ends will be the same, whereas in GPT-4's answer there are two terms at the beginning and three at the end. Given its performance on other problems, it is no surprise that GPT-4 does not spot this anomaly.

On a more positive note, the first two steps – splitting up the sum into three parts and changing variables – are exactly the right thing to do, even if the purpose of changing of variables is not to get the limits to match up (in fact, it does the opposite). However, it then abandons this promising beginning in favour of writing out the sums in a more transparent notation, a move that is striking because it is what many human mathematicians would do if they did not immediately see how to proceed (but presumably GPT-4 has seen texts where this style of explanation is given for why telescoping sums work). It then correctly notes various cancellations, but demonstrates that it is not really understanding what it is saying by jumping to a final answer that is both incorrect and easily seen to be inconsistent with what it has just said. For instance, it notes that $f(2)$ is added once and subtracted twice, but its final answer includes a term $-2f(2)$ instead of $-f(2)$.

1. Additional Supplementary Files

SI Dataset S1 (math_converse_parsed_interactions.csv)
Participant interactions and queries collected in MathConverse.

SI Dataset S2 (annotated_taxonomy.csv)
Annotated participant queries in MathConverse.

SI Dataset S3 (case_studies.pdf)
Full interaction traces from our expert case studies.

SI Software S3 (checkmate_code.zip)
Code for our contributed CheckMate interactive evaluation platform can be included in a zip and is available at our GitHub respository.

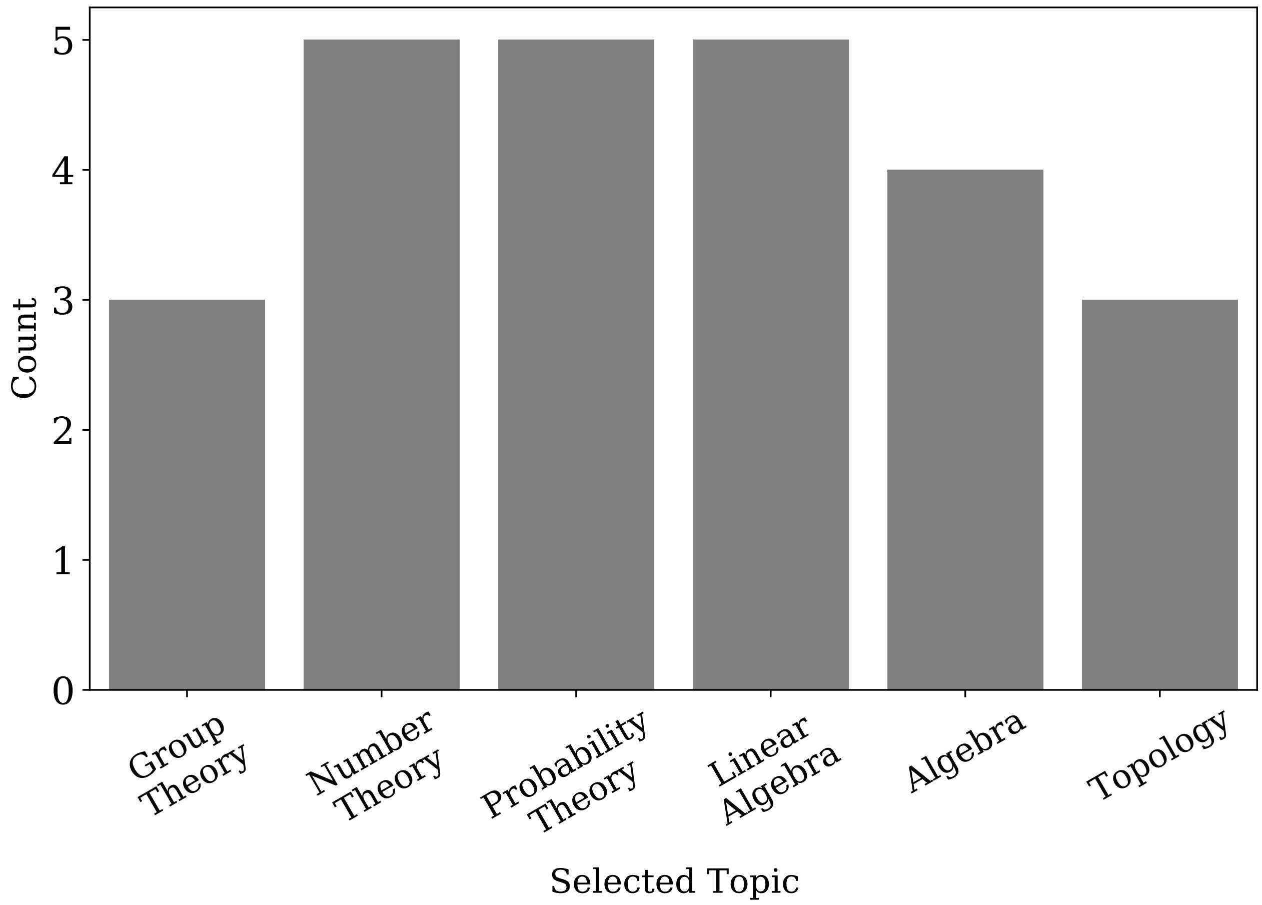

Fig. S1. Topics selected across participants; each participant selected a single topic to evaluate models over.

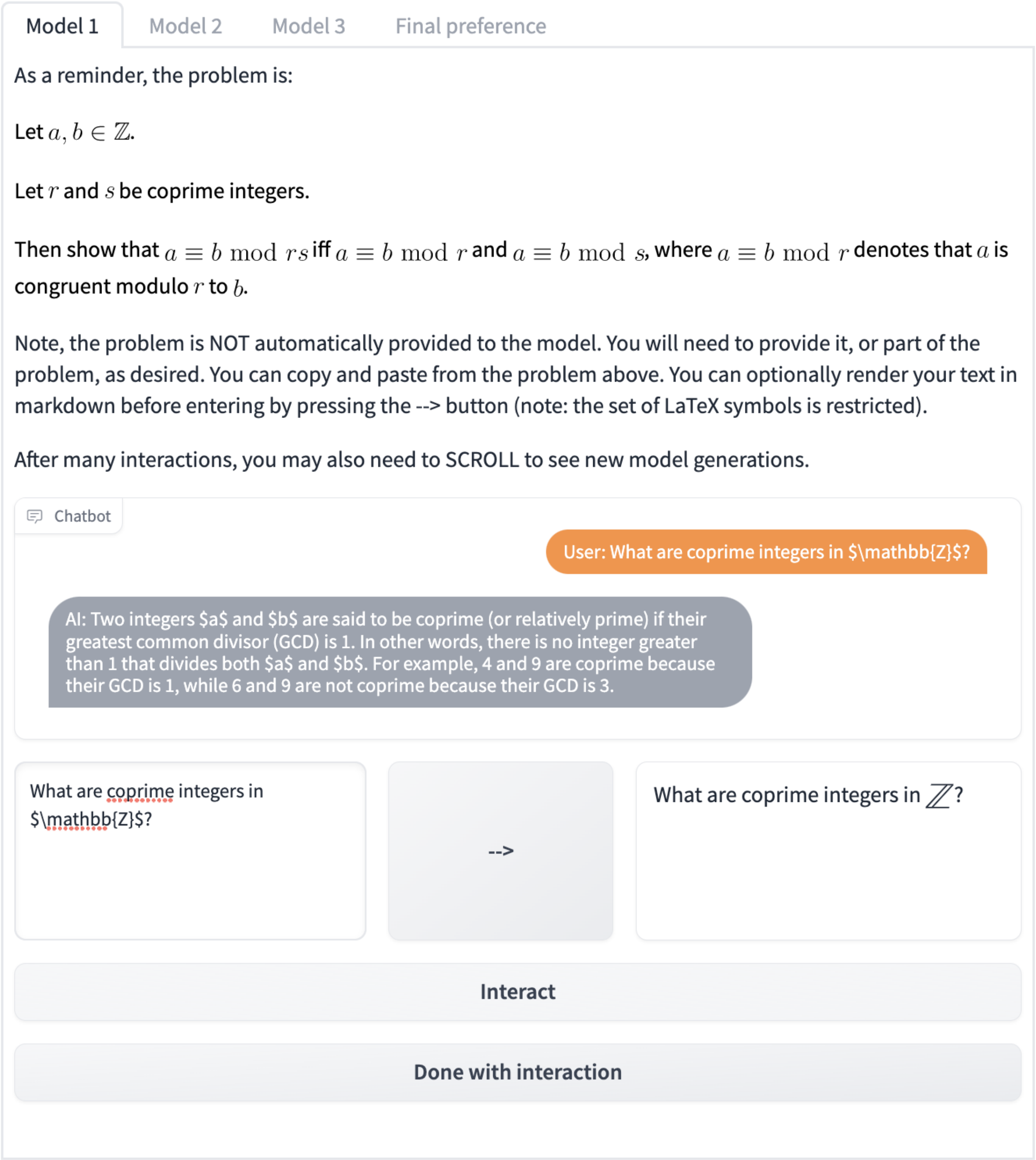

Fig. S2. Example chat interface to interact with an LLM. The participant is presented with the problem and a reminder of particularly relevant instructions, and enters their chat below (which they can see compiled in LaTeX). Chat history is presented for the user to refer to.

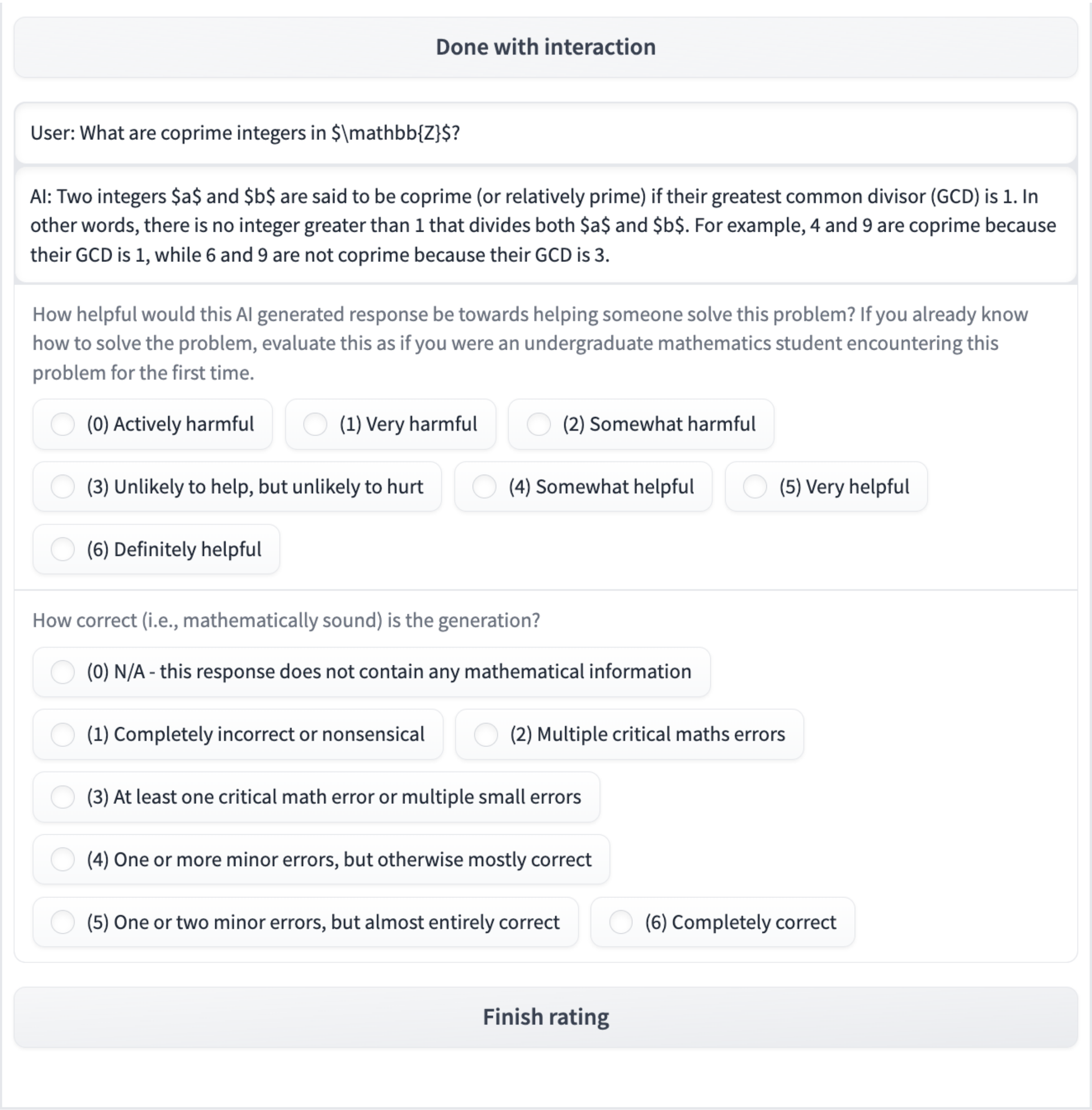

Fig. S3. Example page of post-interaction ratings of each generation, along multiple dimensions. The user is presented with each query-generation pair and rates according to mathematical correctness and perceived helpfulness.

InstructGPT	ChatGPT	GPT4
3	3	1
3	1	1
3	2	2
1	1	1
2	3	2

Table S1. Instances where a participant rated models with a tie. Lower means more preferable for assistance.

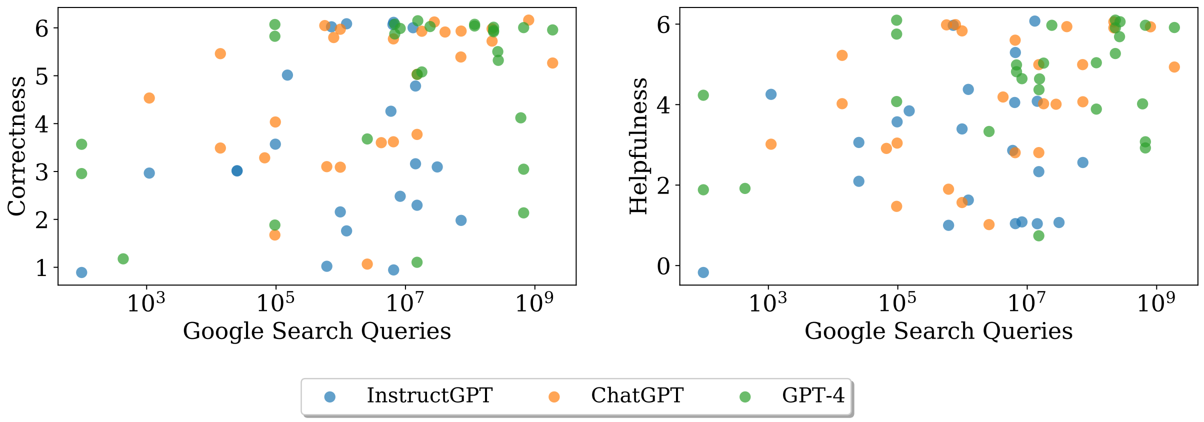

Fig. S4. Correctness and perceived helpfulness ratings, broken down by associated model, against the number of approximate number of Google search queries associated with the original problem at the time of submission. One point per problem, depicting the average rating for that problem. Google queries are conducted over the ProofWiki theorem name associated with the problem.

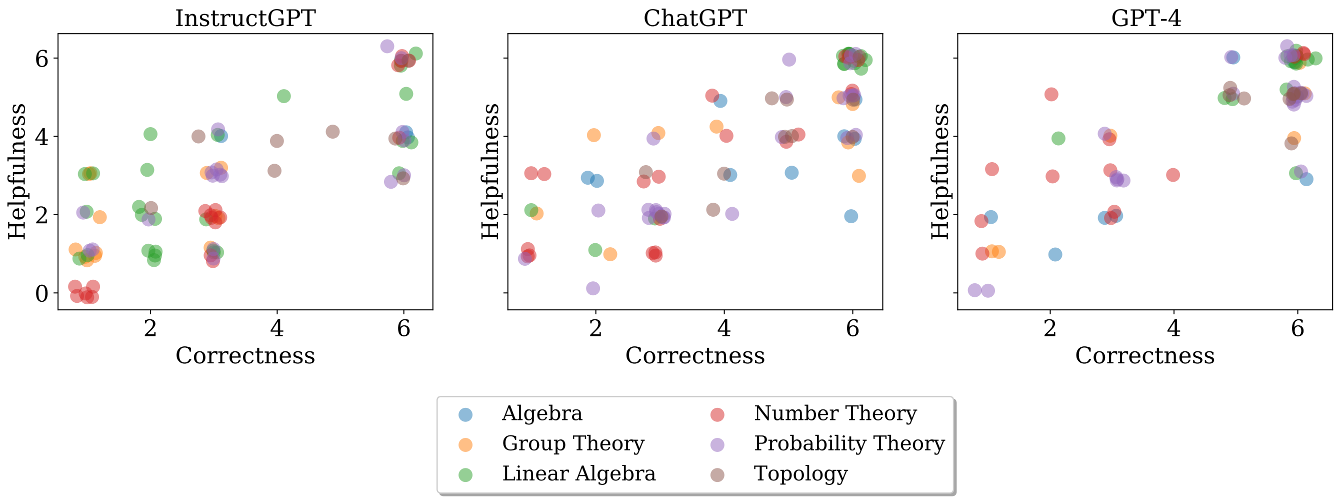

Fig. S5. Correctness and perceived helpfulness ratings, broken down by associated model and colored by problem topic. Slight random jitter is added to horizontal and vertical coordinates of each point to enhance visibility, given several points overlap; each point represents a problem rated by a user.

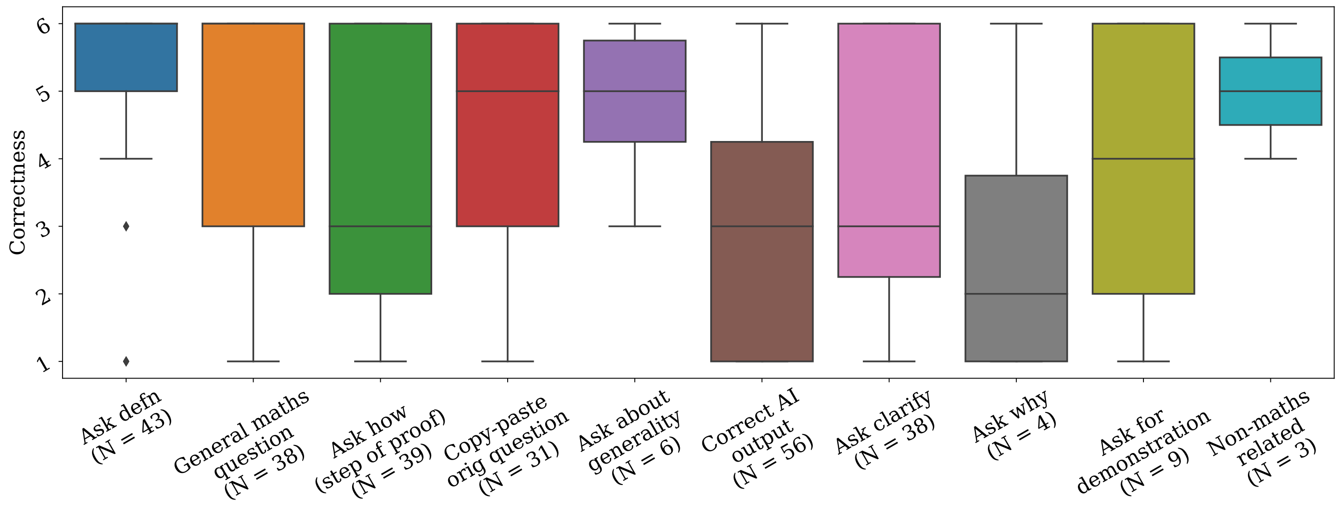

(a)

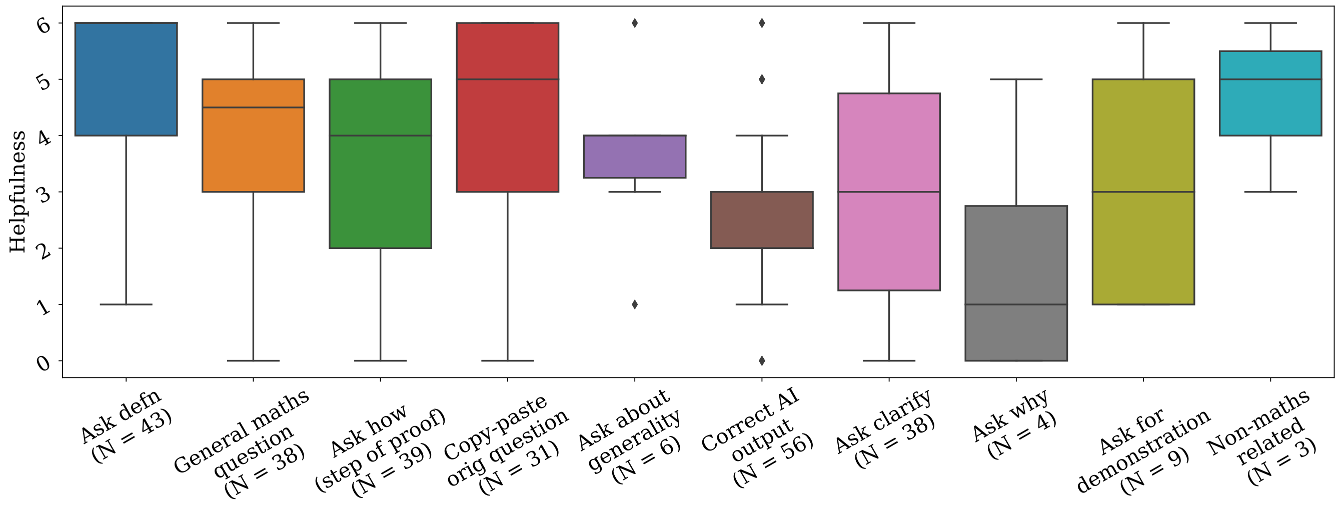

(b)

Fig. S6. Rating of model generations along correctness (a) and helpfulness (b), decomposed by the kind of query the user had asked about. Query type is determined from our taxonomy annotations. N specifies the number of interactions that were classified along a particular query, over the full interaction trace.

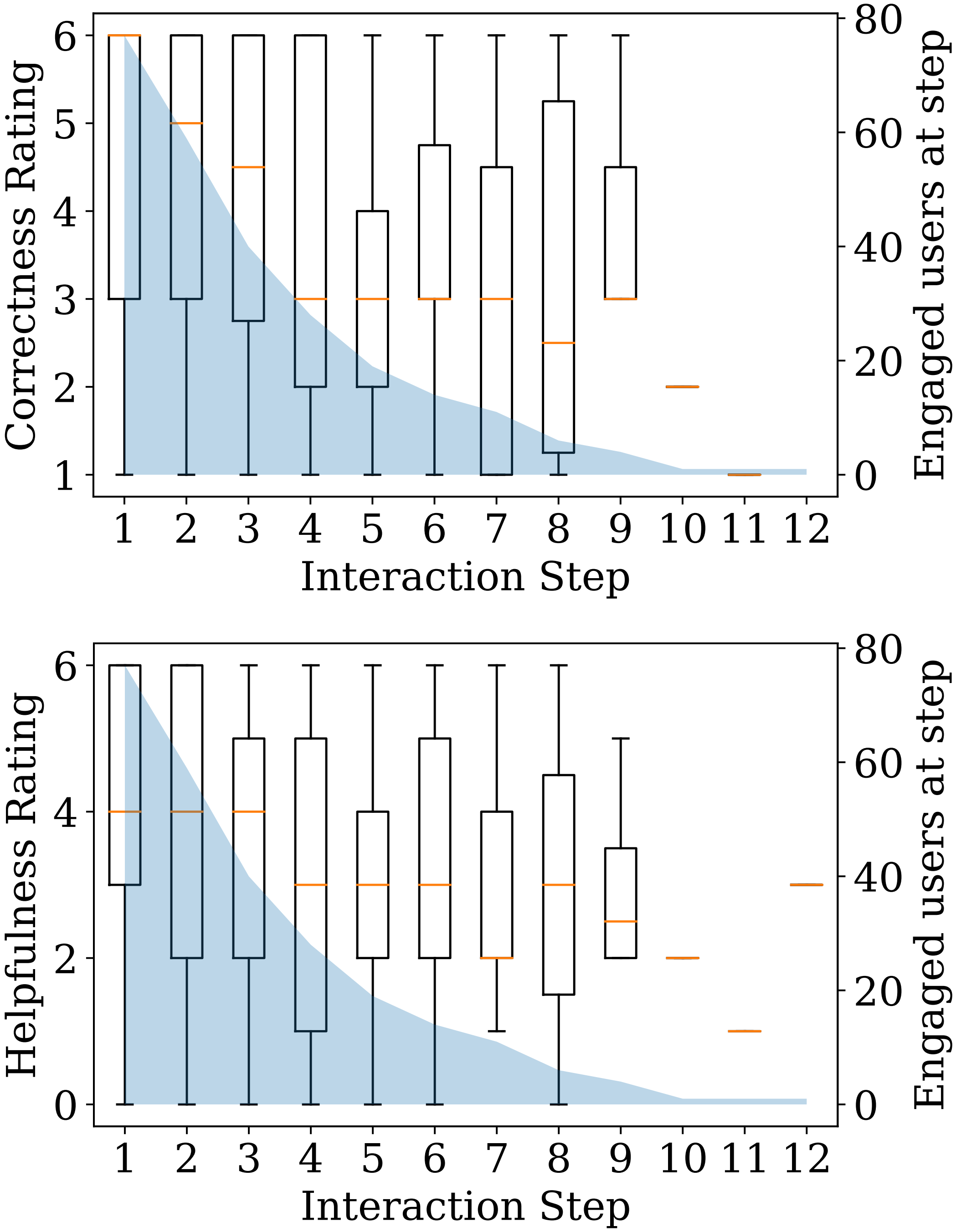

Fig. S7. Depiction of how the users' correctness and helpfulness ratings progress with time. On the horizontal axis is the index of the interaction step. The box plot represents the correctness/helpfulness rating distributions given by users at each step (vertical axis on the left), while the blue shade indicates how many users are still active at that given step (vertical axis on the right). Note, few users undertook more than 9 interactions (3 people undertook 9 interactions, and only one person did 12), hence the boxplot collapses to a line with a single user.

References

1. WW Bledsoe, Non-resolution theorem proving. *Artif. Intell.* **9**, 1–35 (1977).
2. A Bundy, The use of explicit plans to guide inductive proofs in *CADE.* (1988).
3. A Newell, H Simon, The logic theory machine–a complex information processing system. *IRE Transactions on information theory* **2**, 61–79 (1956).
4. A Bundy, The computer modelling of mathematical reasoning. (1983).
5. A Bundy, A Stevens, FV Harmelen, A Ireland, A Smaill, Rippling: A heuristic for guiding inductive proofs. *Artif. Intell.* **62**, 185–253 (1993).
6. H Wang, Toward mechanical mathematics. *IBM J. Res. Dev.* **4**, 2–22 (1960).
7. M Davis, G Logemann, D Loveland, A machine program for theorem-proving. *Commun. ACM* **5**, 394–397 (1962).
8. A Tarski, Truth and proof. *Sci. Am.* **220**, 63–77 (1969).
9. M Ganesalingam, WT Gowers, A fully automatic problem solver with human-style output. *CoRR* **abs/1309.4501** (2013).
10. LM de Moura, NS Bjørner, Z3: An efficient smt solver in *International Conference on Tools and Algorithms for Construction and Analysis of Systems.* (2008).
11. S Schulz, E - a brainiac theorem prover. *AI Commun.* **15**, 111–126 (2002).
12. J Harrison, J Urban, F Wiedijk, History of interactive theorem proving. in *Computational Logic.* Vol. 9, pp. 135–214 (2014).
13. AP Felty, Implementing tactics and tacticals in a higher-order logic programming language. *J. Autom. Reason.* **11**, 41–81 (1993).
14. D Delahaye, A tactic language for the system coq in *Logic for Programming and Automated Reasoning, 7th International Conference, LPAR 2000, Reunion Island, France, November 11-12, 2000, Proceedings*, Lecture Notes in Computer Science, eds. M Parigot, A Voronkov. (Springer), Vol. 1955, pp. 85–95 (2000).
15. LC Paulson, Three years of experience with sledgehammer, a practical link between automatic and interactive theorem provers in *Proceedings of the 2nd Workshop on Practical Aspects of Automated Reasoning, PAAR-2010, Edinburgh, Scotland, UK, July 14, 2010*, EPiC Series in Computing, eds. RA Schmidt, S Schulz, B Konev. (EasyChair), Vol. 9, pp. 1–10 (2010).
16. TC Hales, A proof of the kepler conjecture. *Annals Math.* **162**, 1063–1183 (2005).
17. T Hales, The flyspeck project (2014).
18. AQ Jiang, et al., Draft, sketch, and prove: Guiding formal theorem provers with informal proofs. *CoRR* **abs/2210.12283** (2022).
19. Y Wu, et al., Autoformalization with large language models. *CoRR* **abs/2205.12615** (2022).
20. M Mikula, et al., Magnushammer: A transformer-based approach to premise selection. *CoRR* **abs/2303.04488** (2023).
21. AQ Jiang, et al., Thor: Wielding hammers to integrate language models and automated theorem provers. *CoRR* **abs/2205.10893** (2022).
22. A Lewkowycz, et al., Solving quantitative reasoning problems with language models. *CoRR* **abs/2206.14858** (2022).
23. S Polu, I Sutskever, Generative language modeling for automated theorem proving. *CoRR* **abs/2009.03393** (2020).
24. JM Han, J Rute, Y Wu, EW Ayers, S Polu, Proof artifact co-training for theorem proving with language models in *The Tenth International Conference on Learning Representations, ICLR 2022, Virtual Event, April 25-29, 2022.* (OpenReview.net), (2022).
25. S Polu, et al., Formal mathematics statement curriculum learning. *CoRR* **abs/2202.01344** (2022).
26. P Lu, L Qiu, W Yu, S Welleck, K Chang, A survey of deep learning for mathematical reasoning. *CoRR* **abs/2212.10535** (2022).
27. Z Li, G Poesia, O Costilla-Reyes, ND Goodman, A Solar-Lezama, LEMMA: bootstrapping high-level mathematical reasoning with learned symbolic abstractions. *CoRR* **abs/2211.08671** (2022).
28. G Poesia, ND Goodman, Peano: Learning formal mathematical reasoning. *CoRR* **abs/2211.15864** (2022).
29. E Zelikman, Q Huang, G Poesia, ND Goodman, N Haber, Parsel: A unified natural language framework for algorithmic reasoning. *CoRR* **abs/2212.10561** (2022).
30. G Lample, et al., Hypertree proof search for neural theorem proving. *Adv. Neural Inf. Process. Syst.* **35**, 26337–26349 (2022).
31. S Frieder, et al., Mathematical Capabilities of ChatGPT (2023).
32. S Bubeck, et al., Sparks of Artificial General Intelligence: Early Experiments with GPT-4 (2023).
33. G Sutcliffe, C Suttner, Evaluating general purpose automated theorem proving systems. *Artif. intelligence* **131**, 39–54 (2001).
34. JS Aitken, P Gray, T Melham, M Thomas, Interactive theorem proving: An empirical study of user activity. *J. Symb. Comput.* **25**, 263–284 (1998).
35. B Beckert, S Grebing, F Böhl, A usability evaluation of interactive theorem provers using focus groups in *Software Engineering and Formal Methods: SEFM 2014 Collocated Workshops: HOFM, SAFOME, OpenCert, MoKMaSD, WS-FMDS, Grenoble, France, September 1-2, 2014, Revised Selected Papers 12.* (Springer), pp. 3–19 (2015).
36. OpenAI, GPT-4 Technical Report (2023).
37. R Anil, et al., PaLM 2 Technical Report (2023).
38. H Touvron, et al., LLaMA: Open and efficient foundation language models. *arXiv e-prints* pp. arXiv–2302 (2023).
39. R Taori, et al., Stanford alpaca: An instruction-following llama model (https://github.com/tatsu-lab/stanford_alpaca) (2023).

40. D Dohan, et al., Language model cascades. *ICML* (2022).
41. SM Kazemi, N Kim, D Bhatia, X Xu, D Ramachandran, LAMBADA: Backward Chaining for Automated Reasoning in Natural Language. *arXiv preprint arXiv:2212.13894* (2022).
42. BZ Li, W Chen, P Sharma, J Andreas, Lampp: Language models as probabilistic priors for perception and action. *arXiv e-prints* pp. arXiv–2302 (2023).
43. B Lipkin, L Wong, G Grand, JB Tenenbaum, Evaluating statistical language models as pragmatic reasoners. *arXiv preprint arXiv:2305.01020* (2023).
44. J Wei, et al., Chain-of-thought prompting elicits reasoning in large language models in *Advances in Neural Information Processing Systems.* (2022).
45. D Zhou, et al., Least-to-most prompting enables complex reasoning in large language models. *ICLR* (2023).
46. S Yao, et al., Tree of thoughts: Deliberate problem solving with large language models (2023).
47. M Lee, et al., Evaluating human-language model interaction. *arXiv preprint arXiv:2212.09746* (2022).
48. E Clark, AS Ross, C Tan, Y Ji, NA Smith, Creative writing with a machine in the loop: Case studies on slogans and stories in *Proceedings of IUI.* (2018).
49. AG Cohn, J Hernandez-Orallo, Dialectical language model evaluation: An initial appraisal of the commonsense spatial reasoning abilities of llms. *arXiv preprint arXiv:2304.11164* (2023).
50. H Shen, T Wu, Parachute: Evaluating interactive human-lm co-writing systems. *arXiv e-prints* pp. arXiv–2303 (2023).
51. M Lee, P Liang, Q Yang, CoAuthor: Designing a Human-AI Collaborative Writing Dataset for Exploring Language Model Capabilities in *Proceedings of the 2022 CHI Conference on Human Factors in Computing Systems.* pp. 1–19 (2022).
52. Hugging Face, Hugging face spaces (2021).
53. M Lee, et al., Evaluating human-language model interaction (2022).
54. A Abid, et al., Gradio: Hassle-Free Sharing and Testing of ML Models in the Wild. *arXiv preprint arXiv:1906.02569* (2019).
55. A Radford, et al., Language models are unsupervised multitask learners. (2019).
56. T Brown, et al., Language models are few-shot learners. *NeurIPS* **33**, 1877–1901 (2020).
57. PF Christiano, et al., Deep reinforcement learning from human preferences. *Adv. neural information processing systems* **30** (2017).
58. L Ouyang, et al., Training language models to follow instructions with human feedback (2022).
59. S Casper, et al., Open problems and fundamental limitations of reinforcement learning from human feedback. *arXiv e-prints* pp. arXiv–2307 (2023).
60. EM Bender, T Gebru, A McMillan-Major, S Shmitchell, On the dangers of stochastic parrots: Can language models be too big? in *Proceedings of the 2021 ACM conference on fairness, accountability, and transparency.* pp. 610–623 (2021).
