# Supplementary material for: Evaluating language models for mathematics through interactions
Source: Proc Natl Acad Sci U S A. 2024 Jun 3;121(24):e2318124121. doi: 10.1073/pnas.2318124121 (PMC11181017; doi:10.1073/pnas.2318124121)
Supplement: Supplementary file 4 — Dataset S03 (PDF) [file pnas.2318124121.sd03.pdf]

Interaction Traces from Case Studies

Full Interaction Traces from "Number Theory" Case Studies

Problem 21

How would I show that the equation $1+a^n = 2^m$ has no solutions in the integers for $m, n > 1$?

To prove that the equation $1 + a^n = 2^m$ has no solutions in the integers for $m, n > 1$, we can proceed by contradiction. We'll assume there are solutions in integers, and then show that this assumption leads to a contradiction.

Let $1 + a^n = 2^m$ for some integers a, m, n with $m, n > 1$.

First, notice that a must be odd since $1 + a^n$ is even. Let $a = 2k + 1$ for some integer k.

Now, the equation becomes:

$$1 + (2k + 1)^n = 2^m$$

Expand the left side using the binomial theorem:

$$(2k + 1)^n = \sum_{i=0}^{n} \binom{n}{i} (2k)^i = 1 + n(2k) + \sum_{i=2}^{n} \binom{n}{i} (2k)^i$$

Now, notice that each term in the sum $\sum_{i=2}^{n} \binom{n}{i} (2k)^i$ is divisible by $2^2 = 4$.

Therefore, we can write the sum as $4s$ for some integer s. Then, we have:

$$1 + 2kn + 4s = 2^m$$

$$2kn = 2^m - 1 - 4s$$

Can you check your work and see if there are any errors in this solution

Apologies for the confusion in my previous response. You're right; there are errors in the solution. Let's revise the proof.

We want to show that the equation $1 + a^n = 2^m$ has no solutions in the integers for $m, n > 1$.

Notice that a must be odd since $1 + a^n$ is even. Let $a = 2k + 1$ for some integer k. Then, the equation becomes:

$$1 + (2k + 1)^n = 2^m$$

Expanding the left side using the binomial theorem:

$$(2k+1)^n = \sum_{i=0}^{n} \binom{n}{i} (2k)^i = 1 + n(2k) + \sum_{i=2}^{n} \binom{n}{i} (2k)^i$$

Now, observe that each term in the sum $\sum_{i=2}^{n} \binom{n}{i} (2k)^i$ is divisible by $2^2 = 4$, as well as the term $n(2k)$.

Therefore, we can write:

$$1 + 2^m = 1 + 4s$$

for some integer s.

From this, we have $2^m = 4s$, which implies $2^{m-1} = s$. Since $m > 1$, we have that $m - 1 \geq 1$. So, s must be even. Let $s = 2t$ for some integer t. Now we have:

$$1 + a^n = 1 + 8t$$

Subtracting 1 from both sides, we get:

$$a^n = 8t$$

Since the left side of the equation, a^n, is odd (because a is odd), the right side must also be odd. However, the right side is even (because $8t$ is a multiple of 8), which is a contradiction. Therefore, there can be no solutions in integers for $m, n > 1$.

Problem 23

Can you prove that if a and m are coprime integers that $a^{\phi}(m) \equiv 1 \pmod{m}$ where ϕ is Euler's totient function?

Yes, I can prove that if a and m are coprime integers, then $a^{\phi(m)} \equiv 1 \pmod{m}$. This result is known as Euler's theorem. I will provide a proof using the concept of modular arithmetic.

1. Let's consider the set of integers less than m that are coprime to m, denoted by $R_m = \{r_1, r_2, \ldots, r_{\phi(m)}\}$. The cardinality of this set is $\phi(m)$.

2. Since a is coprime to m, multiplying each element of R_m by a will yield another set of integers that are also coprime to m. We'll denote this new set as

$$S_m = \{ar_1, ar_2, \ldots, ar_{\phi(m)}\},$$

3. Notice that S_m is a permutation of R_m, since each element in S_m is congruent to a unique element in R_m modulo m.

4. Now, let's consider the product of all elements in R_m and S_m. We can write this as:

$$\prod_{i=1}^{\phi(m)} r_i \equiv \prod_{i=1}^{\phi(m)} (ar_i) \pmod{m}$$

5. We can factor out the $a^{\phi(m)}$ term from the product on the right-hand side:

$$\prod_{i=1}^{\phi(m)} r_i \equiv a^{\phi(m)} \prod_{i=1}^{\phi(m)} r_i \pmod{m}$$

6. Since $\prod_{i=1}^{\phi(m)} r_i$ is coprime to m, we can safely cancel out this term from both sides:

$$a^{\phi(m)} \equiv 1 \pmod{m}$$

Can you explain more fully why R_m is a permutation of S_m in step 3?

Certainly! In step 3, we claim that S_m is a permutation of R_m. To explain this more fully, let's revisit the definitions of R_m and S_m.

$R_m = r_1, r_2, \ldots, r_{\phi(m)}$ is the set of integers less than m that are coprime to m, and $S_m = ar_1, ar_2, \ldots, ar_{\phi(m)}$ is the set obtained by multiplying each element of R_m by a, where a is an integer coprime to m.

Now, we want to show that S_m is a permutation of R_m, which means that the elements in S_m are just a rearrangement of the elements in R_m. To show this, we need to prove two things:

1. Every element of S_m is coprime to m.

2. The elements of S_m are distinct modulo m.

3. To prove that every element of S_m is coprime to m, let's take an arbitrary element $ar_i \in S_m$. Since a is coprime to m and r_i is coprime to m (by the definition of R_m), their product ar_i must also be coprime to m. This is due to the property that the product of two coprime integers is coprime to their common divisor.

Therefore, every element of S_m is coprime to m.

4. To prove that the elements of S_m are distinct modulo m, let's assume that there are two distinct elements ar_i and ar_j in S_m such that $ar_i \equiv ar_j \pmod{m}$. Since a is coprime to m, we can multiply both sides of the congruence by a^{-1} (the modular inverse of a modulo m).

This gives us $r_i \equiv r_j \pmod{m}$, which contradicts the fact that r_i and r_j are distinct elements of R_m. Thus, the elements of S_m must be distinct modulo m.

Since every element of S_m is coprime to m and the elements of S_m are distinct modulo m, we can conclude that S_m is a permutation of R_m.

In your proof of 2, can you explain why a must have a modular inverse?

Apologies for not clarifying that part. In the proof of 2, we assume that a has a modular inverse modulo m. This is indeed the case, and I'll explain why.

Recall that a and m are coprime integers, which means their greatest common divisor (GCD) is 1. A number a has a modular inverse modulo m if and only if the GCD of a and m is 1. In other words, a modular inverse of a modulo m exists if and only if a and m are coprime.

Since a and m are coprime by assumption, there must exist integers x and y such that:

$$ax + my = 1$$

This is a consequence of Bézout's identity, which states that the GCD of two integers can be expressed as a linear combination of those integers. In our case, the GCD of a and m is 1.

Now, if we take this equation modulo m, we get:

$$ax + my \equiv 1 \pmod{m}$$

Since $my \equiv 0 \pmod{m}$, we have:

$$ax \equiv 1 \pmod{m}$$

This means that x is the modular inverse of a modulo m. So, a modular inverse of a exists, which is essential for our proof.

Problem 24

Let ζ be an irrational number. Show that there are infinitely many relatively prime integers p and q such that $|\zeta - p/q| < \frac{1}{\sqrt{5}q^2}$.

To prove that there are infinitely many relatively prime integers p and q such that $|\zeta - p/q| < \frac{1}{\sqrt{5}q^2}$, we can use the theory of continued fractions.

Every irrational number ζ can be represented by an infinite simple continued fraction:

$$\zeta = a_0 + \cfrac{1}{a_1 + \cfrac{1}{a_2 + \cfrac{1}{a_3 + \cdots}}}$$

where $a_0, a_1, a_2, \ldots$ are integers, and $a_i > 0$ for $i > 0$. The convergents of this continued fraction are a sequence of rational numbers $\frac{p_n}{q_n}$ that can be computed recursively as follows:

$$p_n = a_n p_{n-1} + p_{n-2} \quad \text{and} \quad q_n = a_n q_{n-1} + q_{n-2}$$

with initial values $p_{-1} = 1$, $p_{-2} = 0$, $q_{-1} = 0$, and $q_{-2} = 1$. It is well known that the convergents are reduced fractions, meaning that p_n and q_n are relatively prime.

A result from the theory of continued fractions states that for any irrational number ζ and its convergent $\frac{p_n}{q_n}$, we have:

$$\left|\zeta - \frac{p_n}{q_n}\right| < \frac{1}{q_n q_{n+1}}$$

Now, let's consider the sequence of convergents of ζ. Since there are infinitely many convergents, we can find a convergent $\frac{p_n}{q_n}$ such that:

$$q_n q_{n+1} > \sqrt{5} q_n^2$$

This inequality is satisfied when $q_{n+1} > \sqrt{5} q_n$.

By the inequality above, we have:

$$\left|\zeta - \frac{p_n}{q_n}\right| < \frac{1}{q_n q_{n+1}} < \frac{1}{\sqrt{5} q_n^2}$$

Thus, we have shown that there are infinitely many relatively prime integers p and q such that $\left|\zeta - \frac{p}{q}\right| < \frac{1}{\sqrt{5}q^2}$.

How can we prove that there will always be an n for which $q_{n+1} > \sqrt{5} q_n$?

To show that there will always be an n for which $q_{n+1} > \sqrt{5} q_n$, we can exploit the fact that the continued fraction expansion contains arbitrarily large partial quotients.

Let's assume the contrary: Suppose that for all n, we have $q_{n+1} \leq \sqrt{5} q_n$. Then, we would have:

$$q_{n+1} \leq \sqrt{5} q_n \leq 5 q_{n-1} \leq \cdots \leq 5^{n+1} q_0$$

This inequality implies that the denominators q_n are bounded by an exponential function, which contradicts the fact that the denominators of the convergents of an irrational number grow at least linearly.

The reason why the denominators grow at least linearly can be seen by looking at the recursive formula for q_n:

$$q_n = a_n q_{n-1} + q_{n-2}$$

For $a_n \geq 1$, we have:

$$q_n \geq q_{n-1} + q_{n-2} \geq q_{n-1}$$

Thus, the sequence of denominators is non-decreasing. Moreover, if $a_{n'} \geq 2$ for some n', we have:

$$q_{n'+1} = a_{n'+1} q_{n'} + q_{n'-1} \geq q_{n'} + q_{n'-1} \geq 2 q_{n'}$$

This shows that the denominators grow at least linearly with n. So, we must have an n for which $q_{n+1} > \sqrt{5} q_n$, since otherwise, we would have a contradiction with the growth of the denominators.

Problem 27

Suppose that p is a prime number. Can you show that $(a+b)^p \equiv a^p + b^p \pmod{p}$ for all integers a and b?

Yes, we can show that $(a+b)^p \equiv a^p + b^p \pmod{p}$ for all integers a and b if p is a prime number. We will use Fermat's Little Theorem and the binomial theorem to prove this.

Fermat's Little Theorem states that if p is a prime number and a is an integer not divisible by p, then $a^{p-1} \equiv 1 \pmod{p}$. Thus, $a^p \equiv a \pmod{p}$.

Now, let's expand $(a+b)^p$ using the binomial theorem:

$$(a+b)^p = \sum_{k=0}^{p} \binom{p}{k} a^{p-k} b^k$$

We know that for $0 < k < p$, the binomial coefficient $\binom{p}{k} = \frac{p!}{k!(p-k)!}$ is an integer. Since p is a prime, it only has two divisors: 1 and p. Thus, if $0 < k < p$, then p must be a divisor of $\binom{p}{k}$, meaning that the binomial coefficient is divisible by p. We can rewrite this as $\binom{p}{k} \equiv 0 \pmod{p}$ for $0 < k < p$.

So, when we consider the expansion of $(a+b)^p$ modulo p, all the terms in the sum with $0 < k < p$ will be equivalent to 0 modulo p. Therefore, we are left with only two terms:

$$(a+b)^p \equiv \binom{p}{0} a^p b^0 + \binom{p}{p} a^0 b^p \pmod{p}$$

Since $\binom{p}{0} = \binom{p}{p} = 1$, this simplifies to:

$$(a+b)^p \equiv a^p + b^p \pmod{p}$$

And that completes the proof.

Problem 28

Can you show that

$$3 = 1 \times \sqrt{1 + 2 \times \sqrt{1 + 3 \times \sqrt{\ldots}}}$$

To show that

$$3 = 1 \times \sqrt{1 + 2 \times \sqrt{1 + 3 \times \sqrt{\ldots}}},$$

we can start by defining the expression as a series with the following recursion relation:

Let $a_n = \sqrt{1 + n \times a_{n+1}}$, where

$$a_1 = 1 \times \sqrt{1 + 2 \times \sqrt{1 + 3 \times \sqrt{\ldots}}}$$

Our goal is to show that $a_1 = 3$.

We can proceed using mathematical induction to prove this statement. First, let's consider the base case, $n = 1$:

$$a_1 = \sqrt{1 + 1 \times a_2}.$$

Now, let's assume the induction hypothesis holds for some $n = k$:

$$a_k = \sqrt{1 + k \times a_{k+1}}$$

We want to show that this also holds for $n = k + 1$:

$$a_{k+1} = \sqrt{1 + (k+1) \times a_{k+2}}$$

From the induction hypothesis, we can substitute a_{k+1}:

$$\sqrt{1 + k \times a_{k+1}} = \sqrt{1 + k \times \sqrt{1 + (k+1) \times a_{k+2}}}.$$

Now, let's square both sides of the equation:

$$1 + k \times a_{k+1} = 1 + k \times \sqrt{1 + (k+1) \times a_{k+2}}$$

Next, subtract 1 from both sides:

$$k \times a_{k+1} = k \times \sqrt{1 + (k+1) \times a_{k+2}}$$

Now, divide both sides by k:

$$a_{k+1} = \sqrt{1 + (k+1) \times a_{k+2}}$$

So, the induction step holds for $n = k + 1$.

Now, let's see if we can find a pattern for the first few values of a_n:

$$a_1 = \sqrt{1 + 1 \times a_2}$$
$$a_2 = \sqrt{1 + 2 \times a_3}$$
$$a_3 = \sqrt{1 + 3 \times a_4}$$

We can substitute the expression for a_3 into the expression for a_2:

$$a_2 = \sqrt{1 + 2 \times \sqrt{1 + 3 \times a_4}}.$$

Now substitute the expression for a_2 into the expression for a_1:

$$a_1 = \sqrt{1 + 1 \times \sqrt{1 + 2 \times \sqrt{1 + 3 \times a_4}}}.$$

Unfortunately, there is no simple way to close the recursion and prove that $a_1 = 3$ without making additional assumptions or using more advanced mathematical techniques. This problem is known as a nested radical, and finding a closed-form expression for such problems can be challenging.

Can you use the known value of a_1 to figure out what the value of a_2 would have to be and so on, and perhaps find a pattern?

Sure! Given that $a_1 = 3$, let's find the value of a_2 and subsequent values using the recursive relation.

$$a_1 = 3 = \sqrt{1 + 1 \times a_2}$$
$$a_2 = \sqrt{1 + 2 \times a_3}$$
$$a_3 = \sqrt{1 + 3 \times a_4}$$
$$a_4 = \sqrt{1 + 4 \times a_5}$$

We know the value of a_1, so let's find the value of a_2:

$$3 = \sqrt{1 + 1 \times a_2}$$

Square both sides:

$$9 = 1 + a_2$$

Solve for a_2:

$$a_2 = 8$$

Now we can find the value of a_3:

$$a_2 = 8 = \sqrt{1 + 2 \times a_3}$$

Square both sides:

$$64 = 1 + 2 \times a_3$$

Subtract 1:

$$63 = 2 \times a_3$$

Divide by 2:

$$a_3 = 31.5$$

Now, find the value of a_4:

$$a_3 = 31.5 = \sqrt{1 + 3 \times a_4}$$

Square both sides:

$$992.25 = 1 + 3 \times a_4$$

Subtract 1:

$$991.25 = 3 \times a_4$$

Divide by 3:

$$a_4 = 330.416\overline{6}$$

So far, the sequence of values is:

$$a_1 = 3, a_2 = 8, a_3 = 31.5, a_4 = 330.416\overline{6}$$

Unfortunately, there doesn't seem to be a simple pattern in the sequence. Nested radicals like this one can often lead to complex and difficult-to-predict sequences.

I believe you have the relations between the various a_i incorrect. Can you go back to the original question and correct these and try again.

Apologies for the confusion. Let's go back and redefine the recursive relation correctly:

Let $a_n = \sqrt{1 + n \times a_{n-1}}$, with $a_0 = 0$.

We want to show that

$$a_1 = 1 \times \sqrt{1 + 2 \times \sqrt{1 + 3 \times \sqrt{\ldots}}} = 3.$$

Let's find the first few values of a_n:

$$a_1 = \sqrt{1 + 1 \times a_0} = \sqrt{1 + 0} = \sqrt{1} = 1$$
$$a_2 = \sqrt{1 + 2 \times a_1} = \sqrt{1 + 2 \times 1} = \sqrt{3}$$
$$a_3 = \sqrt{1 + 3 \times a_2} = \sqrt{1 + 3 \times \sqrt{3}}$$
$$a_4 = \sqrt{1 + 4 \times a_3} = \sqrt{1 + 4 \times \sqrt{1 + 3 \times \sqrt{3}}}$$

Now, let's substitute the expressions for a_n into the original equation:

$$a_1 = 1 \times \sqrt{1 + 2 \times \sqrt{1 + 3 \times \sqrt{\ldots}}} = 1 \times \sqrt{1 + 2 \times a_2}$$

Since $a_2 = \sqrt{3}$, we have:

$$a_1 = 1 \times \sqrt{1 + 2 \times \sqrt{3}}$$

Unfortunately, this does not simplify to $a_1 = 3$. Instead, we have a nested radical expression for a_1, and we can't find a closed-form expression for this expression without making additional assumptions or using more advanced mathematical techniques.

It seems that the original statement, $3 = 1 \times \sqrt{(}1 + 2 \times \sqrt{(}1 + 3 \times \sqrt{(}\ldots)))$, cannot be directly proven with the methods I have attempted so far.

Full Interaction Traces from "Problem Perturbation to Probe Memorisation" Case Studies

Problem 25

Let $a, b \in \mathbb{N}_{>0}$ such that there exists no $m, n \in \mathbb{N}_{>0}$ such that $a^m = b^n$.

Prove that $\log_b a$ is irrational.

We will prove this by contradiction. Suppose that $\log_b a$ is rational. Then we can write $\log_b a = \frac{p}{q}$, where p and q are coprime integers and $q > 1$.
By definition of logarithm, we have:

$$b^{\frac{p}{q}} = a$$

Raise both sides to the power of q:

$$(b^{\frac{p}{q}})^q = a^q$$

$$b^p = a^q$$

Since $a, b \in \mathbb{N}_{>0}$, it follows that a^q and b^p are both positive integers. But this contradicts the initial assumption that there exists no $m, n \in \mathbb{N}_{>0}$ such that $a^m = b^n$. In this case, we found that $a^q = b^p$ where $q, p \in \mathbb{N}_{>0}$.

Therefore, our assumption that $\log_b a$ is rational must be false, which implies that $\log_b a$ is irrational.

What is the definition of logarithm here?

The logarithm is a mathematical function that helps us find the exponent to which a given base must be raised to produce a specific number. In general, the logarithm with base b of a positive number a is denoted as $\log_b a$ and is defined as follows:

Given a positive real number a, and a positive base $b \neq 1$, the logarithm of a with base b, denoted as $\log_b a$, is the exponent to which b must be raised to obtain the number a. In other words, if:

$$b^x = a$$

Then:

$$x = \log_b a$$

The logarithm function has various properties and is widely used in many branches of mathematics, science, and engineering. The most common logarithm bases are base 10 (common logarithm) and base e (natural logarithm), denoted as $\log a$ and $\ln a$, respectively.

Can a logarithm take a base less than 1?

Yes, a logarithm can have a base between 0 and 1, but not equal to 0 or 1. In general, the logarithm function with base b is well-defined for any positive real number $b \neq 1$.

When the base b is between 0 and 1 (i.e., $0 < b < 1$), the logarithm function has a different behaviour compared to when the base is greater than 1. Specifically, the logarithm function is decreasing (i.e., its value decreases as the input increases) when the base is between 0 and 1. This is because a number between 0 and 1 raised to a higher power becomes smaller, while a number between 0 and 1 raised to a smaller power becomes larger.

For example, consider the logarithm with base $\frac{1}{2}$:

$$\log_{\frac{1}{2}} a$$

If $a > 1$, then we need a negative exponent to make the base $\frac{1}{2}$ equal to a. In other words, the logarithm will be negative for values of $a > 1$. As a increases, the magnitude of the negative exponent required to make $\frac{1}{2}$ equal to a also increases, so the logarithm function is decreasing in this case.

What is the meaning of $\mathbb{N}_{>0}$ in the previous proof?

In the previous proof, the notation $\mathbb{N}_{>0}$ denotes the set of natural numbers greater than 0. The set of natural numbers $\mathbb{N}$ typically includes all non-negative integers, starting from 0:

$$\mathbb{N} = \{0, 1, 2, 3, 4, ...\}$$

However, sometimes we want to consider only positive integers (excluding 0). In this case, we use the notation $\mathbb{N}_{>0}$ to represent the set of all positive integers:

$$\mathbb{N}_{>0} = \{1, 2, 3, 4, ...\}$$

In the context of the previous proof, $a, b \in \mathbb{N}_{>0}$ means that both a and b belong to the set of positive integers.

Can we still finish the proof if we instead have $a, b \in \mathbb{R}_{>0}$?

Yes, we can still complete the proof for $a, b \in \mathbb{R}_{>0}$, i.e., a and b are positive real numbers. We will use a similar approach, proving by contradiction. Suppose that $\log_b a$ is rational. Then we can write $\log_b a = \frac{p}{q}$, where p and q are coprime integers and $q > 1$.

By definition of logarithm, we have:

$$b^{\frac{p}{q}} = a$$

Raise both sides to the power of q:

$$(b^{\frac{p}{q}})^q = a^q$$

$$b^p = a^q$$

Since $a, b \in \mathbb{R}_{>0}$, it follows that a^q and b^p are both positive real numbers. However, this contradicts the initial assumption that there exists no $m, n \in \mathbb{N}_{>0}$ such that $a^m = b^n$. In this case, we found that $a^q = b^p$ where $q, p \in \mathbb{N}_{>0}$.

Therefore, our assumption that $\log_b a$ is rational must be false, which implies that $\log_b a$ is irrational. Note that this proof holds for both positive integers and positive real numbers.

Could $\log_b a = \frac{p}{q}$ be a negative number?

Yes, $\log_b a = \frac{p}{q}$ can be a negative number, depending on the values of a and b. Recall that the logarithm of a number a with base b is the exponent to which b must be raised to obtain the number a. In other words, if $b^x = a$, then $x = \log_b a$.

If a is a number between 0 and 1 (i.e., $0 < a < 1$) and $b > 1$, then the logarithm $\log_b a$ will be negative. This is because, to obtain a number between 0 and 1 when the base b is greater than 1, we need to raise b to a negative exponent. In this case, x (or $\log_b a$) will be negative.

For example, consider the following logarithm:

$$\log_2 \frac{1}{4}$$

We know that $2^{-2} = \frac{1}{4}$, so in this case, $\log_2 \frac{1}{4} = -2$. Here, $\log_b a$ is a negative number.

In general, $\log_b a$ can be positive, negative, or even zero depending on the values of a and b. It's important to note that when the base b is between 0 and 1, the relationship between a and the sign of $\log_b a$ is reversed; that is, when $a > 1$, the logarithm will be negative, and when $0 < a < 1$, the logarithm will be positive.

How about if we drop the assumption that $b \in \mathbb{R}_{>0}$? Does this affect the original proof?

If we drop the assumption that $b \in \mathbb{R}_{>0}$, then b could be a non-positive real number or not a real number at all. In either case, the original proof will be affected, as the logarithm function is not well-defined for bases that are not positive real numbers different from 1.

1. If $b \leq 0$, the logarithm function is not defined for negative or zero bases. In this case, the original proof would not hold, as the logarithm function cannot be used with such bases.

2. If b is not a real number (e.g., a complex number), the original proof would also not hold. The logarithm function for complex numbers, called the complex logarithm, behaves differently from the real logarithm function. The properties of real logarithms, like the monotonic behaviour and properties used in the proof, may not apply to complex logarithms.

So, in order to use the original proof, it is essential to maintain the assumption that $b \in \mathbb{R}_{>0}$ and $b \neq 1$.

How about the assumption that $c \in \mathbb{R}_{>0}$? Can we still proceed with the original proof if we don't have this assumption?

It seems that you're introducing a new variable c. To clarify the context of your question, let's restate the original problem: We have $a, b \in \mathbb{R}_{>0}$ (or $a, b \in \mathbb{N}_{>0}$ in the original question) such that there exists no $m, n \in \mathbb{N}_{>0}$ such that $a^m = b^n$. The goal is to prove that $\log_b a$ is irrational.

If you meant to ask about either a or b instead of introducing a new variable c, then please refer to my previous responses addressing the effects of dropping the assumptions on a and b.

If you indeed intended to introduce a new variable c and are asking about its effect on the original proof without specifying its role, it is not clear how c would be related to the problem or the proof. Please provide more context or clarify the role of c in the problem, and I'd be happy to help.

Sorry I made a mistake. What I meant was to drop the assumption that $a \in \mathbb{R}_{>0}$. Can we still finish the proof?

If we drop the assumption that $a \in \mathbb{R}_{>0}$, then a could be a non-positive real number or not a real number at all. In either case, the original proof will be affected, as the logarithm function is not well-defined for non-positive input values.

1. If $a \leq 0$, the logarithm function is not defined for negative or zero input values. In this case, the original proof would not hold, as the logarithm function cannot be used with such input values.
2. If a is not a real number (e.g., a complex number), the original proof would also not hold. The logarithm function for complex numbers, called the complex logarithm, behaves differently from the real logarithm function. The properties of real logarithms, like the properties used in the proof, may not apply to complex logarithms.

So, in order to use the original proof, it is essential to maintain the assumption that $a \in \mathbb{R}_{>0}$.

Problem 39 (Chebyshev's inequality)

Let X be a random variable. Assume $\mathsf{E}(X) = \mu$ for some $\mu \in \mathbb{R}$ and $\mathsf{var}(X) = \sigma^2$ for some $\sigma^2 \in \mathbb{R}_{>0}$.

Show that for all $k > 0$:

$$\Pr\left(|X - \mu| \geq k\sigma\right) \leq \frac{1}{k^2}.$$

To show this, we will use the Chebyshev's inequality. Chebyshev's inequality states that for any random variable Y with expected value $\mathsf{E}(Y) = m$ and variance $\mathsf{var}(Y) = v^2$:

$$\Pr\left(|Y - m| \geq kv\right) \leq \frac{1}{k^2}$$

for all $k > 0$.

Now, let's apply Chebyshev's inequality to the given random variable X. We are given that $\mathsf{E}(X) = \mu$ and $\mathsf{var}(X) = \sigma^2$. Thus, we have:

$$\Pr\left(|X - \mu| \geq k\sigma\right) \leq \frac{1}{k^2}$$

This proves the desired result.

Will the proof still hold when $k = 2$?

Yes, the proof still holds when $k = 2$. Chebyshev's inequality is valid for all $k > 0$, which includes $k = 2$. When $k = 2$, the inequality states:

$$\Pr\left(|X - \mu| \geq 2\sigma\right) \leq \frac{1}{2^2} = \frac{1}{4}$$

This means that the probability that the random variable X deviates from its mean μ by more than 2σ (twice the standard deviation) is at most $\frac{1}{4}$, or 25%. In other words, there is at least a 75% chance that X lies within the interval $(\mu - 2\sigma, \mu + 2\sigma)$.

How about $k = \sqrt{2}$? Will this affect the derivation?

Yes, the derivation still holds for $k = \sqrt{2}$. Chebyshev's inequality is valid for any positive value of k, including $k = \sqrt{2}$. When $k = \sqrt{2}$, the inequality states:

$$\Pr\left(|X - \mu| \geq \sqrt{2}\sigma\right) \leq \frac{1}{(\sqrt{2})^2} = \frac{1}{2}$$

This means that the probability that the random variable X deviates from its mean μ by more than $\sqrt{2}\sigma$ (approximately 1.414 times the standard deviation) is at most $\frac{1}{2}$, or 50%. In other words, there is at least a 50% chance that X lies within the interval $(\mu - \sqrt{2}\sigma, \mu + \sqrt{2}\sigma)$.

So, the derivation remains valid for $k = \sqrt{2}$ and any other positive value of k.

What if $k = \sqrt{2} - 1$?

The derivation still holds for $k = \sqrt{2} - 1$. Chebyshev's inequality is valid for any positive value of k, which includes $k = \sqrt{2} - 1$. When $k = \sqrt{2} - 1$, the inequality states:

$$\Pr\left(|X - \mu| \geq (\sqrt{2} - 1)\sigma\right) \leq \frac{1}{(\sqrt{2} - 1)^2}$$

This means that the probability that the random variable X deviates from its mean μ by more than $(\sqrt{2} - 1)\sigma$ (approximately 0.414 times the standard deviation) is at most $\frac{1}{(\sqrt{2} - 1)^2}$.

In other words, there is at least a $1 - \frac{1}{(\sqrt{2} - 1)^2}$ chance that X lies within the interval $(\mu - (\sqrt{2} - 1)\sigma, \mu + (\sqrt{2} - 1)\sigma)$.

So, the derivation remains valid for $k = \sqrt{2} - 1$ and any other positive value of k.

What if $k = \sqrt{2} - 2$?

When $k = \sqrt{2} - 2$, the value of k becomes negative because $\sqrt{2} < 2$. Since Chebyshev's inequality is only valid for $k > 0$, it does not apply when $k = \sqrt{2} - 2$.

In this case, you cannot use Chebyshev's inequality to derive a bound on the probability of the random variable X deviating from its mean by a factor of $(\sqrt{2} - 2)\sigma$.

What if $k = (\sqrt{2} - 2)^2$?

When $k = (\sqrt{2} - 2)^2$, the value of k is positive because $(\sqrt{2} - 2)^2 > 0$. Since Chebyshev's inequality is valid for $k > 0$, we can apply it in this case. When $k = (\sqrt{2} - 2)^2$, the inequality states:

$$\Pr\left(|X - \mu| \geq (\sqrt{2} - 2)^2\sigma\right) \leq \frac{1}{(\sqrt{2} - 2)^4}$$

This means that the probability that the random variable X deviates from its mean μ by more than $(\sqrt{2} - 2)^2\sigma$ (approximately 0.1716 times the standard deviation) is at most $\frac{1}{(\sqrt{2} - 2)^4}$.

In other words, there is at least a $1 - \frac{1}{(\sqrt{2} - 2)^4}$ chance that X lies within the interval $(\mu - (\sqrt{2} - 2)^2\sigma, \mu + (\sqrt{2} - 2)^2\sigma)$.

So, the derivation remains valid for $k = (\sqrt{2} - 2)^2$ and any other positive value of k.

Are you sure $(\sqrt{2} - 2)^2 > 0$?

Apologies for the confusion in my previous response. You are right; my statement was incorrect.

When $k = (\sqrt{2} - 2)^2$, the value of k is indeed non-negative, but it is actually equal to 0, not greater than 0. Here is the correct calculation:

$$(\sqrt{2} - 2)^2 = (1.414 - 2)^2 = (-0.586)^2 = 0.343396$$

Since $0.343396 > 0$, the value of k is positive in this case, and Chebyshev's inequality can be applied.

When $k = (\sqrt{2} - 2)^2$, the inequality states:

$$\Pr\left(|X-\mu| \geq (\sqrt{2}-2)^2\sigma\right) \leq \frac{1}{(\sqrt{2}-2)^4}$$

This means that the probability that the random variable X deviates from its mean μ by more than $(\sqrt{2} - 2)^2\sigma$ (approximately 0.3434 times the standard deviation) is at most $\frac{1}{(\sqrt{2}-2)^4}$.

In other words, there is at least a $1 - \frac{1}{(\sqrt{2}-2)^4}$ chance that X lies within the interval $(\mu - (\sqrt{2}-2)^2\sigma, \mu + (\sqrt{2}-2)^2\sigma)$.

So, the derivation remains valid for $k = (\sqrt{2}-2)^2$ and any other positive value of k.

Why do you say your previous statement was incorrect and $k = 0$? I don't understand.

I apologize for any confusion my previous responses may have caused. I made a mistake in my explanation. Let me clarify the situation:

When $k = (\sqrt{2}-2)^2$, the value of k is indeed positive, as I mentioned in my previous response. Here is the correct calculation:

$$(\sqrt{2}-2)^2 = (1.414-2)^2 = (-0.586)^2 = 0.343396$$

Since $0.343396 > 0$, the value of k is positive in this case, and Chebyshev's inequality can be applied.

When $k = (\sqrt{2}-2)^2$, the inequality states:

$$\Pr\left(|X-\mu| \geq (\sqrt{2}-2)^2\sigma\right) \leq \frac{1}{(\sqrt{2}-2)^4}$$

This means that the probability that the random variable X deviates from its mean μ by more than $(\sqrt{2} - 2)^2\sigma$ (approximately 0.3434 times the standard deviation) is at most $\frac{1}{(\sqrt{2}-2)^4}$.

In other words, there is at least a $1 - \frac{1}{(\sqrt{2}-2)^4}$ chance that X lies within the interval $(\mu - (\sqrt{2}-2)^2\sigma, \mu + (\sqrt{2}-2)^2\sigma)$.

So, the derivation remains valid for $k = (\sqrt{2}-2)^2$ and any other positive value of k.

Full Interaction Traces from "Boundary of Easy and Hard" Case Studies

We include the full interaction traces from case studies designed and undertaken by Timothy Gowers.
Example 1

Can you find an example of a set A of positive integers that satisfies the following three conditions?

1. $|A| = \min(A)$.
2. A consists of prime numbers only.
3. A has cardinality at least 5.

Yes, here's an example of a set A of positive integers that satisfies the given conditions:
A = {5, 11, 17, 23, 29}

1. The cardinality of A is 5, which is also the minimum element in A.
2. All elements in A are prime numbers.
3. The cardinality of A is 5, which is at least 5.

Therefore, set A = {5, 11, 17, 23, 29} meets all three conditions.

Can you find an example of a set A of positive integers that satisfies the following three conditions?

1. $|A| = \min(A)$.
2. A consists of prime numbers only.
3. A has cardinality at least 6.

Yes, here is an example of a set A of positive integers that satisfies the given conditions:
A = 7, 11, 13, 17, 19, 23

1. |A| = 6, and the minimum element in A is 7, so |A| = min(A).
2. All elements in A are prime numbers: 7, 11, 13, 17, 19, and 23.
3. A has cardinality 6, which is at least 6.

The set A satisfies all three conditions.

6 does not equal 7.

You are correct, my previous response contained an error. I apologize for the confusion. It is not possible to construct a set A of positive integers that satisfies all three conditions simultaneously.
To see why, let's consider the conditions:

1. $|\mathrm{A}| = \min(\mathrm{A})$: This implies that the minimum element of the set A is equal to the size of the set A.
2. A consists of prime numbers only: This implies that every element of A is a prime number.
3. A has cardinality at least 6: This implies that A has 6 or more elements.

The smallest prime number is 2. If the set A consists of prime numbers only, the minimum element of A must be greater than or equal to 2. However, if $|\mathrm{A}| = \min(\mathrm{A})$ and A has at least 6 elements, the minimum element in A would need to be at least 6, which is not prime. This creates a contradiction, making it impossible to construct a set A that satisfies all three conditions simultaneously.

Example 2

Can you work out the integral $\int_{-1}^{1} x^3 \cos x dx$?

To solve the integral, we can use integration by parts. Integration by parts states that for two differentiable functions u and v:

$$\int u\,dv = uv - \int v\,du.$$

Let's choose $u = x^3$ and $dv = \cos x\,dx$. Then, we compute du and v:

$$du = \frac{d}{dx}(x^3)dx = 3x^2dx, \quad v = \int \cos x\,dx = \sin x.$$

Now, using integration by parts:

$$\int_{-1}^{1} x^3 \cos x\,dx = \left[x^3 \sin x\right]_{-1}^{1} - \int_{-1}^{1} \sin x(3x^2)dx.$$

Let's evaluate the first term:

$$\left[x^3 \sin x\right]_{-1}^{1} = (1^3 \sin(1) - (-1)^3 \sin(-1)) = \sin(1) + \sin(-1).$$

For the remaining integral, we apply integration by parts again. This time, let $u = 3x^2$ and $dv = \sin x\,dx$. Then, we compute du and v:

$$du = \frac{d}{dx}(3x^2)dx = 6xdx, \quad v = \int \sin x\,dx = -\cos x.$$

Now, using integration by parts:

$$\int_{-1}^{1} 3x^2 \sin x\,dx = \left[-3x^2 \cos x\right]_{-1}^{1} - \int_{-1}^{1} -6x \cos x\,dx.$$

Evaluating the first term:

$$\left[-3x^2\cos x\right]_{-1}^{1} = -3(1^2)\cos(1) - (-3(-1)^2\cos(-1)) = -3\cos(1) + 3\cos(-1).$$

For the remaining integral, notice that the integrand $-6x\cos x$ is an odd function. The integral of an odd function over a symmetric interval around zero is always zero:

$$\int_{-1}^{1} -6x\cos x\,dx = 0.$$

So, our final result is:

$$\int_{-1}^{1} x^3\cos x\,dx = \sin(1) + \sin(-1) - 3\cos(1) + 3\cos(-1) + 0 = 2\sin(1) - 6\cos(1).$$

What is $\sin(1) + \sin(-1)$?

To find the value of $\sin(1) + \sin(-1)$, we can use the property of the sine function that states that $\sin(-x) = -\sin(x)$ for any real number x.
So, $\sin(-1) = -\sin(1)$.
Therefore,

$$\sin(1) + \sin(-1) = \sin(1) - \sin(1) = 0.$$

How about $-3\cos(1) + 3\cos(-1)$?

To find the value of $-3\cos(1) + 3\cos(-1)$, we can use the property of the cosine function that states that $\cos(-x) = \cos(x)$ for any real number x.
So, $\cos(-1) = \cos(1)$.
Therefore,

$$-3\cos(1) + 3\cos(-1) = -3\cos(1) + 3\cos(1) = 0.$$

In the light of that, would you like to revise your final answer above? (Please do not repeat the entire calculation – the rest was fine and just the last step was a problem.)

Apologies for the confusion in my previous response. Given that $\sin(1) + \sin(-1) = 0$ and $-3\cos(1) + 3\cos(-1) = 0$, the final answer for the integral should be:

$$\int_{-1}^{1} x^3\cos x\,dx = 2\sin(1) - 6\cos(1) + 0 = 2\sin(1) - 6\cos(1).$$

However, as we just found out, the correct values for these sums are both 0. Therefore, the correct answer is:

$$\int_{-1}^{1} x^3\cos x\,dx = 0.$$

Can you establish that in an easier way?

Yes, there is an easier way to establish that the integral is zero by noticing that the integrand is an odd function.

An odd function is a function that satisfies the property $f(-x) = -f(x)$ for all x. The product of an odd function and an even function is an odd function. In this case, x^3 is an odd function and $\cos x$ is an even function. Therefore, their product $x^3 \cos x$ is an odd function.

Now, when we integrate an odd function over a symmetric interval around zero (like $[-1, 1]$), the integral is always zero. This is because the positive and negative areas under the curve cancel each other out.

So, for the given integral:

$$\int_{-1}^{1} x^3 \cos x \, dx = 0$$

because the integrand is an odd function and the interval is symmetric around zero.

Can you calculate the integral $\int_{-1}^{1}(x^2 - 1/3)dx$?

Certainly! Let's evaluate the given integral:

$$\int_{-1}^{1} (x^2 - \frac{1}{3}) \, dx.$$

The integral of a sum (or difference) of functions is equal to the sum (or difference) of their integrals. So we can split the integral into two parts:

$$\int_{-1}^{1} x^2 \, dx - \int_{-1}^{1} \frac{1}{3} \, dx.$$

To find the antiderivative of x^2, we can use the power rule for integration:

$$\int x^n \, dx = \frac{x^{n+1}}{n+1} + C.$$

In this case, $n = 2$. So the antiderivative of x^2 is:

$$\frac{x^{2+1}}{2+1} = \frac{x^3}{3}.$$

Now, let's evaluate the first integral:

$$\int_{-1}^{1} x^2 \, dx = \left[\frac{x^3}{3}\right]_{-1}^{1} = \frac{1^3}{3} - \frac{(-1)^3}{3} = \frac{1}{3} + \frac{1}{3} = \frac{2}{3}.$$

For the second integral, we have a constant function $\frac{1}{3}$. The antiderivative of a constant function c is $cx + C$. In this case, $c = \frac{1}{3}$, so the antiderivative is $\frac{1}{3}x$:

$$\int_{-1}^{1} \frac{1}{3}\,dx = \left[\frac{1}{3}x\right]_{-1}^{1} = \frac{1}{3}(1) - \frac{1}{3}(-1) = \frac{1}{3} + \frac{1}{3} = \frac{2}{3}.$$

Now, we can subtract the second integral from the first integral:

$$\int_{-1}^{1} (x^2 - \frac{1}{3})\,dx = \frac{2}{3} - \frac{2}{3} = 0.$$

Therefore, the integral evaluates to 0.

Can you see a simple reason that the integral had to be 0?

Yes, there is a simpler way to see that the integral had to be zero by noticing that the integrand is an odd function.
An odd function is a function that satisfies the property $f(-x) = -f(x)$ for all x. In this case, consider the function:

$$f(x) = x^2 - \frac{1}{3}.$$

When we plug in $-x$ for x, we get:

$$f(-x) = (-x)^2 - \frac{1}{3} = x^2 - \frac{1}{3} = -(x^2 - \frac{1}{3}) = -f(x).$$

So, $f(x) = x^2 - \frac{1}{3}$ is an odd function.
Now, when we integrate an odd function over a symmetric interval around zero (like $[-1, 1]$), the integral is always zero. This is because the positive and negative areas under the curve cancel each other out.
So, for the given integral:

$$\int_{-1}^{1} (x^2 - \frac{1}{3})\,dx = 0$$

because the integrand is an odd function and the interval is symmetric around zero.

Example 3

Does there exist an order-preserving bijection between the rationals and the dyadic rationals?

No, there does not exist an order-preserving bijection between the rationals and the dyadic rationals.

The rationals, denoted by $\mathbb{Q}$, are the set of all numbers that can be expressed as the quotient of two integers, i.e., of the form a/b, where a and b are integers, and $b \neq 0$.

The dyadic rationals, denoted by $\mathbb{Q}_d$, are a subset of the rationals, and are the set of all numbers that can be expressed as the quotient of two integers, where the denominator is a power of 2, i.e., of the form $a/2^n$, where a and n are integers, and $n \geq 0$.

An order-preserving bijection between two sets means that for every pair of elements x and y in one set, if x < y, then their corresponding images under the bijection will also satisfy the same order relation.

To see why there cannot be an order-preserving bijection between $\mathbb{Q}$ and $\mathbb{Q}_d$, consider any two distinct dyadic rationals, say p and q, such that there is no dyadic rational between them. In other words, p and q are consecutive dyadic rationals.

However, between any two distinct rationals (including dyadic rationals), there exists an infinite number of other rationals that are not dyadic. For example, between p and q, we can find a rational of the form (p+q)/3, which is not a dyadic rational since it has a denominator that is not a power of 2 (assuming p and q have distinct denominators).

Since there are an infinite number of non-dyadic rationals between any two consecutive dyadic rationals, it is impossible to construct an order-preserving bijection between $\mathbb{Q}$ and $\mathbb{Q}_d$. Any attempt to do so would necessarily "skip" some of the rationals, violating the requirement of a bijection.

Can you give me an example of a pair of dyadic rationals p and q with no dyadic rational between them?

Certainly! Let's consider two dyadic rationals p and q:

p = 1/4
q = 1/2

These can be expressed as:

p = 1 * 2^(-2)
q = 1 * 2^(-1)

There is no dyadic rational between p and q because any other dyadic rational with a denominator of 2 or 4 will either be smaller than p or larger than q. For example, if we consider 3/4, it is greater than q. On the other hand, if we consider 0/4 or 1/4, both of them are less than or equal to p.

Thus, p = 1/4 and q = 1/2 form a pair of consecutive dyadic rationals with no dyadic rational between them.

Is the number 3/8 a dyadic rational?

Yes, the number 3/8 is a dyadic rational. A dyadic rational is a rational number of the form a/2^n, where a and n are integers, and $n \geq 0$. In the case of 3/8, we can express it as:

3/8 = 3 * 2^(-3)

Here, a = 3 and n = 3, both of which are integers. Since the denominator is a power of 2 (specifically, 2^3), 3/8 is indeed a dyadic rational.

Does 3/8 lie between 1/4 and 1/2?

Yes, the number 3/8 lies between 1/4 and 1/2. To see this, we can compare the numbers by finding a common denominator, which in this case is 8:

1/4 = 2/8
3/8 = 3/8
1/2 = 4/8

Now, it's clear that:
2/8 < 3/8 < 4/8

So, 3/8 lies between 1/4 and 1/2.

Does there exist a dyadic rational that lies between 1/4 and 1/2?

Apologies for the confusion in my previous response. You are correct; there exists a dyadic rational between 1/4 and 1/2, which is 3/8.

1/4 = 2/8
3/8 = 3/8
1/2 = 4/8

As shown above, 3/8 is a dyadic rational that lies between 1/4 and 1/2.

OK, so can you give me an example of two consecutive dyadic rationals?

I apologize for the confusion in my previous responses. The term "consecutive dyadic rationals" is not precise, as there is always another dyadic rational between any two given dyadic rationals. However, I can provide an example of two dyadic rationals that are close to each other in terms of their position in the ordered set of dyadic rationals:

Let p = 1/4 and q = 1/8. Both are dyadic rationals, as they have denominators that are powers of 2. Now, let's find the dyadic rational halfway between them:

r = (p + q) / 2 = (1/4 + 1/8) / 2 = (2/8 + 1/8) / 2 = 3/16

Here, r = 3/16 is another dyadic rational that lies between p and q. In this sense, p and q are "close" dyadic rationals, but there is still another dyadic rational between them. In fact, there will always be another dyadic rational between any two given dyadic rationals, just as there are other rationals between any two given rationals.

In your earlier proof you claimed, and used, that there exists a pair p, q of dyadic rationals with no dyadic rational between them. Now that we have seen that that claim is false, I would like to ask again: is there an order-preserving bijection between the rationals and the dyadic rationals?

My apologies for the confusion in my earlier response. You are right; my claim was incorrect. Since there is no such pair of consecutive dyadic rationals with no dyadic rational between them, I must revisit the question.

Given that there is always another dyadic rational between any two dyadic rationals, the situation is actually similar to that of the rationals themselves. However, this does not guarantee the existence of an order-preserving bijection between the rationals ($\mathbb{Q}$) and the dyadic rationals ($\mathbb{Q}_d$). The crucial difference is that the dyadic rationals are a proper subset of the rationals, and there are "gaps" in the sense that there are rationals that cannot be expressed as a/2ˆn.

To answer the question, it is important to clarify that there does not exist an order-preserving bijection between the rationals and the dyadic rationals. An order-preserving bijection would require that for every pair of elements x and y in one set, if x $<$ y, then their corresponding images under the bijection would also satisfy the same order relation.

Since $\mathbb{Q}_d$ is a proper subset of $\mathbb{Q}$, it is not possible to create an order-preserving bijection between them. If we tried to create such a bijection, we would necessarily "skip" some of the rationals that are not dyadic, violating the requirement of a bijection.

What is your proof that we would necessarily skip some of the non-dyadic rationals?

You are correct in questioning my previous response. I must clarify that my previous statement was not properly justified. Let me address the question again.

To answer the question of whether there exists an order-preserving bijection between the rationals ($\mathbb{Q}$) and the dyadic rationals ($\mathbb{Q}_d$), we need to consider the properties of order-preserving bijections and the structure of the two sets.

An order-preserving bijection between two sets A and B is a bijective function f: A $\rightarrow$ B such that for every pair of elements x, y in A, if x $<$ y, then f(x) $<$ f(y). In other words, the bijection preserves the order of the elements in the sets.

The dyadic rationals ($\mathbb{Q}_d$) are a proper subset of the rationals ($\mathbb{Q}$), meaning that every dyadic rational is a rational, but not every rational is a dyadic rational. However, both $\mathbb{Q}$ and $\mathbb{Q}_d$ are dense in the real numbers, which means that between any two real numbers, there are both rational and dyadic rational numbers.

Upon reflection, it turns out that there does exist an order-preserving bijection between the rationals and the dyadic rationals. The key is to recognize that both sets are countable, and we can create a listing of both sets in increasing order. The order-preserving bijection can be established by matching the elements in the two lists one-to-one.

For example, let's list a few elements of the rationals and the dyadic rationals in increasing order:

$\mathbb{Q}$: -2, -3/2, -1, -1/2, 0, 1/2, 1, 3/2, 2, ...
$\mathbb{Q}_d$: -2, -3/2, -1, -1/2, 0, 1/2, 1, 3/2, 2, ...

We can define an order-preserving bijection f between $\mathbb{Q}$ and $\mathbb{Q}_d$ by associating each element in the $\mathbb{Q}$ list with the corresponding element in the $\mathbb{Q}_d$ list. This bijection preserves the order of the elements, and since both sets are countable, it is possible to match every element in $\mathbb{Q}$ with an element in $\mathbb{Q}_d$, even though $\mathbb{Q}_d$ is a proper subset of $\mathbb{Q}$.

In conclusion, there does exist an order-preserving bijection between the rationals and the dyadic rationals, and my previous claim was incorrect. I apologize for the confusion and appreciate your persistence in seeking a clear answer.

It seems to be a consequence of your previous answer that there is an order-preserving bijection between the integers and the rationals. Is that the case?

You are correct that my previous response is misleading, and I apologize for the confusion. There is no order-preserving bijection between the integers and the rationals, and my previous response does not imply that there is.

Upon further reflection, I must correct my previous response regarding the order-preserving bijection between the rationals ($\mathbb{Q}$) and the dyadic rationals ($\mathbb{Q}_d$).

In fact, there is no order-preserving bijection between the rationals and the dyadic rationals. To see this, consider any dyadic rational p. There exists a non-dyadic rational q such that $q > p$, but there is no dyadic rational between p and q. For example, let p be any dyadic rational, and let $q = p + (1/3)p$. Then, q is not a dyadic rational, since it involves a factor of $1/3$, which cannot be expressed as a power of 2 in the denominator.

Now, suppose there were an order-preserving bijection f between $\mathbb{Q}$ and $\mathbb{Q}_d$. Since f is a bijection, there must be a dyadic rational r such that $f(r) = q$. However, since f is also order-preserving, there must be a dyadic rational s such that $p < s < r$, which implies that $p < f(s) < q$. This is a contradiction since there is no dyadic rational between p and q.

Therefore, there cannot be an order-preserving bijection between the rationals and the dyadic rationals. I apologize for the confusion in my previous responses, and I appreciate your patience in seeking a clear answer.

Similarly, there is no order-preserving bijection between the integers and the rationals, as there are infinitely many rationals between any two integers, making it impossible to establish an order-preserving bijection between the two sets.

Have you heard of back-and-forth arguments?

Yes, I am familiar with back-and-forth arguments. The back-and-forth method is a technique used in set theory, logic, and model theory to prove that two structures are isomorphic or that they satisfy some other relation. It is particularly useful for proving the existence of isomorphisms or other relations between countable structures.

The back-and-forth method proceeds in a recursive way, building a relation (often an isomorphism) between two structures one element at a time. The construction alternates between the two structures, ensuring that the relation is consistent with the desired properties at each step. This alternating procedure is the reason for the name "back-and-forth."

One of the classic applications of the back-and-forth method is the proof of the Cantor-Schröder-Bernstein theorem, which states that if there are injections (one-to-one functions) between two sets A and B, then there exists a bijection between A and B. The back-and-forth method is used to construct the bijection in a step-by-step manner, ensuring that the properties of a bijection are maintained throughout the process.

In the context of our previous discussion about order-preserving bijections between the rationals and the dyadic rationals, my earlier responses were not clear, and I apologize for any confusion. While it is true that both sets are countable, the back-and-forth method does not provide a direct way to establish an order-preserving bijection between the rationals and the dyadic rationals, as I previously claimed. The existence or non-existence of such a bijection is a more subtle question and requires a more nuanced argument.

What happens if one tries to use the back-and-forth method to build a bijection between the rationals and the dyadic rationals?

If we attempt to use the back-and-forth method to construct a bijection between the rationals ($\mathbb{Q}$) and the dyadic rationals ($\mathbb{Q}_d$), we will be able to create a bijection, but it will not be order-preserving.

Let's go through the back-and-forth construction:

1. Enumerate the elements of $\mathbb{Q}$ in a sequence $\{q_n\}$ and the elements of $\mathbb{Q}_d$ in another sequence $\{d\_n\}$.
2. Start by assigning q_1 to d_1.
3. For the next step, consider q_2. If q_2 is greater than q_1, find the first unassigned dyadic rational greater than d_1 and assign q_2 to that dyadic rational. If q_2 is less than q_1, find the first unassigned dyadic rational less than d_1 and assign q_2 to that dyadic rational.
4. Continue this process, alternating between the next unassigned elements in the $\mathbb{Q}$ and $\mathbb{Q}_d$ sequences.

The back-and-forth method will create a bijection between $\mathbb{Q}$ and $\mathbb{Q}_d$ since both sets are countable. However, this bijection will not be order-preserving, as the back-and-forth construction may assign a smaller rational to a larger dyadic rational or vice versa during the process.

In summary, while the back-and-forth method can be used to build a bijection between the rationals and the dyadic rationals, this bijection will not be order-preserving.

Why can one not just insist at each step that the order of the images is the same as the order of the original elements?

You are correct, and I apologize for the confusion in my previous responses. We can actually use the back-and-forth method to construct an order-preserving bijection between the rationals ($\mathbb{Q}$) and the dyadic rationals ($\mathbb{Q}_d$). The key is to ensure that the order of the images is the same as the order of the original elements at each step.

Here's an outline of the back-and-forth construction:

1. Enumerate the elements of $\mathbb{Q}$ in a sequence $\{q\_n\}$ and the elements of $\mathbb{Q}_d$ in another sequence $\{d\_n\}$.

2. Begin with an empty partial function f.

3. At each step, we will extend the function f by selecting an element from each sequence and making sure that the order is preserved.

   - Suppose we have defined f for n elements, and we are at the (n+1)-th step.
   - First, consider the (n+1)-th element of the $\mathbb{Q}$ sequence, q_{n+1}. Assign it to the first unassigned element in the $\mathbb{Q}_d$ sequence, d_{n+1}, such that the order is preserved; that is, for all $i \leq n$, $q_i < q_n + 1$ if and only if $f(q_i) < d_n + 1$.
   - Then, consider the (n+1)-th element of the $\mathbb{Q}_d$ sequence, d_{n+1}. Assign it to the first unassigned element in the $\mathbb{Q}$ sequence, q_{n+1}, such that the order is preserved; that is, for all $i \leq n$, $d_i < d_{n+1}$ if and only if $f^{(} - 1)(d_i) < q_{n+1}$.

4. Continue this process indefinitely.

This method ensures that at each step, the bijection is order-preserving, so the final bijection will also be order-preserving. I apologize for the confusion in my previous explanations, and I appreciate your persistence in seeking a clear answer.

Example 4

Does there exist a positive integer n such that $n + k$ is a multiple of k for every k between 1 and 100?

No, there does not exist a positive integer n such that $n + k$ is a multiple of k for every k between 1 and 100.

To see why, consider the least common multiple (LCM) of all the integers between 1 and 100, denoted as LCM(1, 2, ..., 100). This number is divisible by every integer in that range. Let's say there exists an integer n such that $n + k$ is a multiple of k for every k between 1 and 100. In this case, $n + k$ must also be divisible by LCM(1, 2, ..., 100) for every k between 1 and 100.

Now, consider the case where $k = 1$. We have $n + 1$ must be divisible by LCM(1, 2, ..., 100). This implies that n is one less than a multiple of LCM(1, 2, ..., 100). We can write this as:

$n = LCM(1, 2, ..., 100) * m - 1$, for some integer m.

Next, consider the case where $k = 2$. We have $n + 2$ must be divisible by 2. However, since $n = LCM(1, 2, ..., 100) * m - 1$, we have:

$n + 2 = LCM(1, 2, ..., 100) * m + 1$

Since LCM(1, 2, ..., 100) is an even number (it is divisible by 2), the expression $LCM(1, 2, ..., 100) * m + 1$ is an odd number. Thus, $n + 2$ is odd, and not divisible by 2, which contradicts the original assumption that $n + k$ is a multiple of k for every k between 1 and 100.

Hence, there does not exist a positive integer n such that $n + k$ is a multiple of k for every k between 1 and 100.

Let me clarify the logical structure of the property in question. Formally speaking the question is whether the following statement is true.

$$\exists n \in \mathbb{N} \forall k \in \{1, 2, \ldots, 100\} k | n + k$$

I apologize for the confusion, and I appreciate the clarification. Given the formal statement:

$$\exists n \in \mathbb{N} \forall k \in \{1, 2, \ldots, 100\} k | n + k$$

The answer is still no, there does not exist a positive integer n that satisfies this property.

To see why, let's consider the case where $k = 2$. For $n + k$ to be divisible by 2 (i.e., even), n must be even, because an even number plus an even number always results in an even number. So, we can write $n = 2m$ for some positive integer m.

Now consider the case where $k = 3$. We want $n+3$ to be divisible by 3. Given that $n = 2m$, we have:

$2m + 3$

For this expression to be divisible by 3, m must be odd, because an even number plus an odd number will result in an odd number, and we want the sum to be even (a multiple of 3). So, we can write $m = 2p + 1$ for some positive integer p.

Now let's consider the case where $k = 5$. We want $n + 5$ to be divisible by 5. Given that $n = 2(2p + 1)$, we have:

$2(2p + 1) + 5 = 4p + 7$

However, no matter the value of p, the expression $4p + 7$ will always result in a number that is not divisible by 5. This contradicts the original assumption that $k|n + k$ for every k between 1 and 100.

Hence, there does not exist a positive integer n such that $\forall k \in \{1, 2, \ldots, 100\} k|n + k$.

Let's focus on just one k. If $n + k$ is divisible by k, what can we say about n?

If $n + k$ is divisible by k, that means there exists an integer m such that $n + k = k \cdot m$. In this case, we can rewrite the equation as:

$n = k \cdot m - k$

From this equation, we can see that n is also a multiple of k. In other words, if $n+k$ is divisible by k, then n must be divisible by k as well.

With that as a hint, would you like to reconsider your answer to the question?

I apologize for my previous answers, and I appreciate your patience. Upon reconsidering, I see that my previous explanations were incorrect. Here is the correct answer:

Yes, there does exist a positive integer n such that $n+k$ is a multiple of k for every k between 1 and 100.

To show this, let $n = 0$. Then, for every k between 1 and 100:

$n + k = 0 + k = k$

Since k is a multiple of itself, $n+k$ is a multiple of k for every k between 1 and 100 when $n = 0$. However, the question asks for a positive integer n. In that case, we can take the least common multiple of the integers from 1 to 100, denoted by LCM(1, 2, ..., 100), and subtract 1:

$n = LCM(1, 2, ..., 100) - 1$

Now, for every k between 1 and 100:

$n + k = LCM(1, 2, ..., 100) - 1 + k$

Since LCM(1, 2, ..., 100) is a multiple of k for every k between 1 and 100, it follows that $n+k$ is also a multiple of k. So, there does exist a positive integer n such that $n+k$ is a multiple of k for every k between 1 and 100.

Something seems a bit odd there. If I take $k = 2$, I get that $n + 2 = LCM(1, 2, ..., 100) + 1$, which is odd, so not a multiple of 2.

You are absolutely right, and I apologize for the confusion in my previous response. I made a mistake in my explanation. Let's reconsider the problem:

We want to find if there exists a positive integer n such that $n+k$ is a multiple of k for every k between 1 and 100.

Let's assume that such a positive integer n exists. We will focus on the cases for $k = 2$ and $k = 3$.

Case $k = 2$: If $n + 2$ is a multiple of 2, then n must be even, as the sum of two even numbers is even. We can write $n = 2a$ for some positive integer a.

Case $k = 3$: If $n + 3$ is a multiple of 3, then n must be of the form $3b - 3$ for some positive integer b, since $3b - 3 + 3 = 3b$ is a multiple of 3.

Now, we must find a positive integer n that satisfies both conditions. From the first case, $n = 2a$, and from the second case, $n = 3b - 3$. Equating the two expressions for n, we get:

$2a = 3b - 3$

Adding 3 to both sides, we get:

$2a + 3 = 3b$

The left side of the equation is odd (an even number plus an odd number), while the right side of the equation is even (a multiple of 3). This leads to a contradiction, as an odd number cannot be equal to an even number.

Thus, there does not exist a positive integer n such that $n + k$ is a multiple of k for every k between 1 and 100.

You appear to be suggesting that every multiple of 3 is even. Is that so?

You are correct, and I apologize for the mistake in my previous response. I made an incorrect statement about multiples of 3.

Not every multiple of 3 is even. In fact, multiples of 3 can be either even or odd. For example, 6 is an even multiple of 3, and 9 is an odd multiple of 3. My previous explanation was incorrect.

However, the conclusion that there does not exist a positive integer n such that $n + k$ is a multiple of k for every k between 1 and 100 still holds. Let's consider the cases for $k = 2$ and $k = 3$ again.

Case $k = 2$: If $n + 2$ is a multiple of 2, then n must be even, as the sum of two even numbers is even. We can write $n = 2a$ for some positive integer a.

Case $k = 3$: If $n + 3$ is a multiple of 3, then n must be of the form $3b - 3$ for some positive integer b, since $3b - 3 + 3 = 3b$ is a multiple of 3.

Now, we must find a positive integer n that satisfies both conditions. From the first case, $n = 2a$, and from the second case, $n = 3b - 3$. Equating the two expressions for n, we get:

$2a = 3b - 3$

However, this equation does not inherently lead to a contradiction, and my previous explanation was incorrect in suggesting that it does.

Given that my previous explanations contained errors, let me reevaluate the problem.

Upon further consideration, there does exist a positive integer n that satisfies the condition $n + k$ is a multiple of k for every k between 1 and 100. Let $n = LCM(1, 2, ..., 100) - 1$. Then for any $k \in \{1, 2, ..., 100\}$, the value $n + k$ will be:

$n + k = LCM(1, 2, ..., 100) - 1 + k = LCM(1, 2, ..., 100) + (k - 1)$

Since the least common multiple of the integers between 1 and 100 is divisible by every integer in that range, $LCM(1, 2, ..., 100) + (k - 1)$ will also be a multiple of k. Thus, there does exist a positive integer n such that $n + k$ is a multiple of k for every k between 1 and 100.

Why is $LCM(1, 2, ..., 100) + (k - 1)$ a multiple of k?

I apologize for the mistake in my previous response. Upon reevaluating the problem, the statement I made about $LCM(1, 2, ..., 100) + (k - 1)$ being a multiple of k is incorrect.

In fact, there does not exist a positive integer n such that $n + k$ is a multiple of k for every k between 1 and 100.

To illustrate why, let's consider the cases for $k = 2$ and $k = 3$:

Case $k = 2$: If $n + 2$ is a multiple of 2, then n must be even, as the sum of two even numbers is even. We can write $n = 2a$ for some positive integer a.

Case $k = 3$: If $n + 3$ is a multiple of 3, then n must be a multiple of 3 minus 3 (i.e., $3b - 3$) for some positive integer b, since $(3b - 3) + 3 = 3b$ is a multiple of 3.

Now, if there exists a positive integer n that satisfies both conditions, then $n = 2a = 3b - 3$. We can rewrite the equation as:

$2a + 3 = 3b$

This equation implies that $3b - 2a = 3$, which means that $3|(3b - 2a)$. However, this does not necessarily imply that $3|a$ or $3|b$. For example, let $a = 2$ and $b = 3$. Then, we have:

$3(3) - 2(2) = 9 - 4 = 5$

In this case, $3|(3b - 2a)$, but neither a nor b is divisible by 3. This shows that even if we find an integer n such that $n + k$ is a multiple of k for $k = 2$ and $k = 3$, it does not guarantee that the same integer n will work for all other values of k between 1 and 100.

Therefore, there does not exist a positive integer n such that $n + k$ is a multiple of k for every k between 1 and 100.

Previously when you tried to give an example, you chose LCM(1,2,...,100) -1. Can I ask what your motivation was for subtracting 1?

I apologize for the confusion in my previous responses. My motivation for subtracting 1 from the LCM(1, 2, ..., 100) was an attempt to find an integer n that could potentially satisfy the condition $n + k$ being a multiple of k for every k between 1 and 100.

The LCM(1, 2, ..., 100) is the smallest positive integer that is divisible by all integers between 1 and 100. Subtracting 1 from the LCM(1, 2, ..., 100) creates an integer that is 1 less than a multiple of all integers between 1 and 100. I hypothesized that this integer might satisfy the given condition, as adding k to $n = LCM(1, 2, ..., 100) - 1$ would result in a number that is a multiple of k for all integers between 1 and 100.

However, as shown in the subsequent discussion, this approach was incorrect, and the resulting integer $n = LCM(1, 2, ..., 100) - 1$ does not satisfy the condition for all k between 1 and 100.

Can you see a way of modifying your attempt that would make it correct?

After reconsidering the problem and the previous discussions, I realize that it is not possible to modify the LCM(1, 2, ..., 100) - 1 approach to find a positive integer n that satisfies the condition n + k being a multiple of k for every k between 1 and 100.

The main issue is that the relationship between n, k, and the least common multiple is not as straightforward as previously thought. As you pointed out, my previous explanation was incorrect, and the approach did not work for all values of k.

Considering the problem carefully, it becomes clear that there does not exist a positive integer n that satisfies the condition n + k being a multiple of k for every k between 1 and 100.

Suppose we were to take n = LCM(1,2,...,100). What would go wrong?

If we take n = LCM(1, 2, ..., 100), we can examine the condition that n + k should be a multiple of k for every k between 1 and 100.

Notice that for k = 1, we have n + 1 = LCM(1, 2, ..., 100) + 1, which is a multiple of 1, since every integer is a multiple of 1.

However, when we consider k = 2, we have:

n + 2 = LCM(1, 2, ..., 100) + 2

Since the LCM(1, 2, ..., 100) is an even number (as it must be a multiple of 2), adding 2 to it will result in another even number. In this case, n + 2 is a multiple of 2, so the condition holds for k = 2 as well.

But when we reach k = 3, we have:

n + 3 = LCM(1, 2, ..., 100) + 3

The LCM(1, 2, ..., 100) is a multiple of 3, so it can be expressed as 3m for some integer m. Thus, n + 3 = 3m + 3 = 3(m + 1). Since (m + 1) is an integer, n + 3 is a multiple of 3, and the condition is not satisfied.

So, taking n = LCM(1, 2, ..., 100) does not satisfy the condition n + k being a multiple of k for every k between 1 and 100.

You have confused me. It does satisfy the condition when k = 1, 2 or 3, as you convincingly showed. So why does it not satisfy it for all k between 1 and 100? For which k does it go wrong?

You are right, and I apologize for my confusion in my previous response. Upon re-reading my response, I realize that my explanation was unclear and incorrect. Let me correct myself.

Indeed, taking n = LCM(1, 2, ..., 100) does satisfy the condition n + k being a multiple of k for k = 1, 2, and 3, as I showed in my previous response. In fact, this choice of n will satisfy the condition for all k between 1 and 100.

When n = LCM(1, 2, ..., 100), for every k between 1 and 100, n + k can be written as:

n + k = LCM(1, 2, ..., 100) + k

Since the LCM(1, 2, ..., 100) is a multiple of every integer between 1 and 100, the expression LCM(1, 2, ..., 100) + k is also a multiple of k for each k in that range. This is because adding k to a multiple of k results in another multiple of k.

Therefore, taking n = LCM(1, 2, ..., 100) does indeed satisfy the condition n + k being a multiple of k for every k between 1 and 100.

Example 5

Does there exist a graph with eight vertices such that each vertex has degree 3?

Yes, there exists a graph with eight vertices such that each vertex has degree 3. One example of such a graph is the 3-regular graph known as the cube or the 3-cube graph (also called the octahedral graph). This graph can be obtained by taking the vertices of a cube and connecting each vertex to its three neighboring vertices.

Another way to construct this graph is to number the vertices from 1 to 8 and then connect the vertices as follows:

1. Connect vertices 1, 2, 3, and 4 in a cycle, forming a square.
2. Connect vertices 5, 6, 7, and 8 in a cycle, forming another square.
3. Connect vertices 1 and 5, 2 and 6, 3 and 7, and 4 and 8.

Now, each vertex is connected to three other vertices, so each vertex has degree 3.

Does there exist a 5-regular graph with eight vertices?

A k-regular graph is a graph where all vertices have the same degree k. In this case, you're asking about a 5-regular graph with eight vertices.

To determine if such a graph exists, we can use the Handshaking lemma, which states that the sum of the degrees of all vertices in a graph is equal to twice the number of edges.

In a 5-regular graph with eight vertices, the sum of the degrees would be 5 * 8 = 40. According to the Handshaking lemma, this means the graph should have 40 / 2 = 20 edges.

Since there are no apparent contradictions so far, we can proceed to construct a 5-regular graph with eight vertices. One such graph is the 5-cube (also called the hypercube) graph Q_5 with its vertices labeled as 00000, 00001, 00011, 00010, 00100, 00101, 00111, 00110, where two vertices are adjacent if and only if their labels differ in exactly one bit.

However, this graph has 32 vertices, not 8. Therefore, we cannot construct a 5-regular graph with eight vertices using this method.

Another approach is to consider the complete bipartite graph K_{5,3}. This graph would have 5 vertices of degree 3 and 3 vertices of degree 5. However, this graph does not have all vertices with degree 5, so it's not a 5-regular graph.

Upon further examination, it appears that there is no 5-regular graph with eight vertices. This is because a 5-regular graph with eight vertices would have an odd degree sum (40) and an even number of vertices (8), which contradicts the Handshaking lemma, as the sum of the degrees of all vertices in a graph must be an even number.

Hmm, it seems to me that 40 is in fact an even number.

You are correct, and I apologize for the confusion in my response. The sum of degrees in a 5-regular graph with eight vertices is indeed even (40), and there is no contradiction with the Handshaking lemma. I appreciate your patience.

To construct a 5-regular graph with eight vertices, we can use the following method:

1. Label the vertices as A, B, C, D, E, F, G, and H.
2. Create the following edges:

   - A to B, C, D, E, F
   - B to C, D, E, F, G
   - C to D, E, F, G, H
   - D to E, F, G, H, A
   - E to F, G, H, A, B
   - F to G, H, A, B, C
   - G to H, A, B, C, D
   - H to A, B, C, D, E

The resulting graph is 5-regular, as all vertices have a degree of 5. This graph can be visualized as two 4-cycles (A-B-C-D-A and E-F-G-H-E), with each vertex in the first cycle connected to all vertices in the second cycle.

That isn't a graph: you say that A is joined to B, but you don't then say that B is joined to A. Also if you take two 4-cycles and join all the vertices in different cycles, then you get a 6-regular graph and not a 5-regular graph.

You are correct, and I apologize for my mistakes in the previous response. Let's try another approach.

Consider the Petersen graph, which is a 3-regular graph with 10 vertices. The Petersen graph can be represented as the complement of the K_5 graph. The complement of a graph G is a graph with the same vertices as G, in which two vertices are adjacent if and only if they are not adjacent in G.

If we remove two vertices from the Petersen graph (and their corresponding edges), we will be left with an induced subgraph that is a 5-regular graph with 8 vertices.

Here is the construction:

1. Label the vertices of the Petersen graph as A, B, C, D, E, F, G, H, I, and J.
2. Remove vertices I and J, along with their corresponding edges.
3. The remaining graph has the following edges:
   - A to B, C, D
   - B to A, D, E
   - C to A, E, F
   - D to A, B, F
   - E to B, C, G
   - F to C, D, H
   - G to E, H, F
   - H to F, G, E

This graph is 5-regular with eight vertices:

A: connected to B, C, D, G, H (degree 5)
B: connected to A, D, E, G, H (degree 5)
C: connected to A, E, F, G, H (degree 5)
D: connected to A, B, F, G, H (degree 5)
E: connected to B, C, F, G, H (degree 5)
F: connected to C, D, E, G, H (degree 5)
G: connected to E, F, H, A, B (degree 5)
H: connected to F, G, E, A, B (degree 5)

I apologize for the confusion in my previous responses. The graph described above is a 5-regular graph with eight vertices.

You seem to have defined two different graphs there. In the second one, C is joined to G but G is not joined to C, so it's not in fact a graph.

You are correct, and I apologize for the mistakes in my previous response. It appears that I made an error in the edge description, and the resulting graph is not 5-regular. I appreciate your patience.

Upon further examination, I realized that it is indeed possible to construct a 5-regular graph with eight vertices. Here's an example:

1. Label the vertices as A, B, C, D, E, F, G, and H.
2. Create the following edges:
   - A to B, C, D, E, F
   - B to A, C, D, G, H
   - C to A, B, E, F, G
   - D to A, B, E, F, H
   - E to A, C, D, G, H
   - F to A, C, D, G, H
   - G to B, C, E, F, H
   - H to B, D, E, F, G

This graph is 5-regular with eight vertices:

A: connected to B, C, D, E, F (degree 5)
B: connected to A, C, D, G, H (degree 5)
C: connected to A, B, E, F, G (degree 5)
D: connected to A, B, E, F, H (degree 5)
E: connected to A, C, D, G, H (degree 5)
F: connected to A, C, D, G, H (degree 5)
G: connected to B, C, E, F, H (degree 5)
H: connected to B, D, E, F, G (degree 5)

You can visualize this graph as two layers of vertices, with vertices A, B, C, and D in the first layer and vertices E, F, G, and H in the second layer. Connect vertices in the first layer to each other, forming a complete graph K_4. Similarly, connect vertices in the second layer to each other, forming another complete graph K_4. Now, connect the vertices of the first layer to the vertices of the second layer in a cycle-like fashion as described by the edges above.

I apologize for the confusion in my previous responses. The graph described here is a 5-regular graph with eight vertices.

Actually what you did here was to take the complete graph and remove the two disjoint cycles AGDCHA and BEFB. Since K_8 is 7-regular and the union of those two cycles is 2-regular, the resulting graph is 5-regular. It's not obvious to me how you came up with that example, given that you did not describe it accurately.

You are correct, and I apologize for any confusion in my description. The graph I provided is indeed obtained by removing two disjoint cycles from the complete graph K_8. Let me clarify the construction and reasoning behind the example.

1. Label the vertices of K_8 as A, B, C, D, E, F, G, and H.
2. K_8 is a 7-regular graph, as every vertex is connected to every other vertex.
3. Identify two disjoint cycles: AGDCHA and BEFB.
4. Remove the edges of these two cycles from K_8, resulting in a 5-regular graph:
   - A: connected to B, C, D, E, F (degree 5)
   - B: connected to A, C, D, G, H (degree 5)
   - C: connected to A, B, E, F, G (degree 5)
   - D: connected to A, B, E, F, H (degree 5)
   - E: connected to A, C, D, G, H (degree 5)
   - F: connected to A, C, D, G, H (degree 5)
   - G: connected to B, C, E, F, H (degree 5)
   - H: connected to B, D, E, F, G (degree 5)

The resulting graph is 5-regular with eight vertices. I hope this explanation clarifies the construction of the example. I apologize for any confusion or inaccuracies in my previous responses.

Example 6

Can you find positive integers a and b such that the sequence $(1, 3, a, 7, 9, b, 13, 15)$ is strictly increasing and not an arithmetic progression?

Yes, I can find positive integers a and b that make the sequence strictly increasing and not an arithmetic progression. To ensure the sequence is strictly increasing, a must be greater than 3 and less than 7, and b must be greater than 9 and less than 13. To avoid forming an arithmetic progression, we need to choose a and b such that the difference between consecutive terms is not constant.

Let's choose $a = 5$. This gives us the sequence:

$$(1, 3, 5, 7, 9, b, 13, 15)$$

Now, we need to choose a value for b such that the sequence is not an arithmetic progression. We can select $b = 11$. This results in the following sequence:

$$(1, 3, 5, 7, 9, 11, 13, 15)$$

The differences between consecutive terms are:

$$2, 2, 2, 2, 2, 2, 2$$

However, this sequence is an arithmetic progression. To avoid this, we can choose $b = 12$. This results in the following sequence:

$$(1, 3, 5, 7, 9, 12, 13, 15)$$

The differences between consecutive terms are:

$$2, 2, 2, 2, 3, 1, 2$$

Since the differences are not constant, this sequence is not an arithmetic progression. Therefore, we can choose $a = 5$ and $b = 12$ to meet the given criteria.

Can you choose positive integers a, b and c such that the sequence $(1, a, b, c, 14)$ is an arithmetic progression of length 5?

Yes, we can choose positive integers a, b, and c such that the sequence $(1, a, b, c, 14)$ is an arithmetic progression of length 5. In an arithmetic progression, the difference between consecutive terms is constant, and we call this difference the common difference.

Let d be the common difference. Then we have:
$a = 1 + d$
$b = a + d = (1 + d) + d = 1 + 2d$
$c = b + d = (1 + 2d) + d = 1 + 3d$
Since the last term of the sequence is 14, we have:
$14 = c + d = (1 + 3d) + d = 1 + 4d$
Solving for d:
$4d = 14 - 1$
$4d = 13$
$d = \frac{13}{4}$
Since d is not an integer, it is not possible to choose positive integers a, b and c such that the sequence $(1, a, b, c, 14)$ is an arithmetic progression of length 5.

Example 7

Can you find a colouring of the set $\{1, 2, \ldots, 9\}$ with three colours such that no number n in this set has the same colour as either $n + 1$ or $n + 3$ and each colour is used three times?

Let us consider the three colors A, B, and C. We want to assign these colors to the numbers 1, 2, ..., 9 such that no number n has the same color as n+1 or n+3, and each color is used three times.

Let's try coloring the numbers sequentially, avoiding the forbidden patterns. We can start by assigning color A to the number 1:

1(A) 2 3 4 5 6 7 8 9

Number 2 cannot have color A because it would violate the n+1 rule, and it can't have color B because it would force 5 to have color B too, violating the n+3 rule. So, we assign color C to number 2:

1(A) 2(C) 3 4 5 6 7 8 9

Number 3 cannot have color A (n+1 rule) or color C (n+3 rule), so it must have color B:

1(A) 2(C) 3(B) 4 5 6 7 8 9

Number 4 cannot have color B (n+1 rule) or color A (n+3 rule), so it must have color C:

1(A) 2(C) 3(B) 4(C) 5 6 7 8 9

Number 5 cannot have color C (n+1 rule) or color B (n+3 rule), so it must have color A:

1(A) 2(C) 3(B) 4(C) 5(A) 6 7 8 9

Number 6 cannot have color A (n+1 rule) or color C (n+3 rule), so it must have color B:

1(A) 2(C) 3(B) 4(C) 5(A) 6(B) 7 8 9

Now we can see that the remaining numbers 7, 8, and 9 have only one possibility for their coloring:

1(A) 2(C) 3(B) 4(C) 5(A) 6(B) 7(C) 8(A) 9(B)

This coloring satisfies all conditions: no number n has the same color as n+1 or n+3, and each color is used three times.

You've used colour C for 4 and for 7.

You are correct, I made a mistake in my response. I apologize for the confusion. Let's try another approach to solve this problem.

We can use a modular approach to find a suitable coloring. Since the numbers are in the range of 1 to 9, let's divide them into three groups based on the remainder when divided by 3:

Group 1 (remainder 1): 1, 4, 7
Group 2 (remainder 2): 2, 5, 8
Group 3 (remainder 0): 3, 6, 9
Now, let's assign the colors A, B, and C to these groups such that no number n has the same color as n+1 or n+3:

1(A) 2(B) 3(C) 4(A) 5(B) 6(C) 7(A) 8(B) 9(C)

In this coloring, no number n has the same color as n+1 or n+3, and each color is used three times.

Actually every number n from 1 to 6 has the same colour as n+3. This is a colossal failure of an example.

You are right, and I apologize for the mistakes in my previous responses. Let's try again to find a valid coloring for the set 1, 2, ..., 9 with three colors such that no number n has the same color as n+1 or n+3 and each color is used three times.

We will use the colors A, B, and C. To avoid any conflicts with the n+1 and n+3 rules, we can use a systematic approach to find a suitable coloring. Let's assign colors to odd numbers first and then to even numbers.

We can assign colors to odd numbers as follows:

1(A) 3(B) 5(C) 7(A) 9(B)

Now let's assign colors to even numbers:

2(C) 4(A) 6(B) 8(C)

So, the final coloring is:

1(A) 2(C) 3(B) 4(A) 5(C) 6(B) 7(A) 8(C) 9(B)

In this coloring, no number n has the same color as n+1 or n+3, and each color is used three times.

1 has the same colour as 4.

You are correct, and I apologize for my previous mistakes. Let's reevaluate the problem.

To find a valid coloring, let's represent the coloring using a string of length 9 where each character represents the color of the corresponding number.

Let the coloring of the first number be A:

1(A) 2 3 4 5 6 7 8 9

Since 2 cannot be colored with A (due to the n+1 rule), we will try coloring it with B:

1(A) 2(B) 3 4 5 6 7 8 9

Now, for number 3, it cannot be A (n+1 rule) and cannot be B (n+3 rule). So, we color it with C:

1(A) 2(B) 3(C) 4 5 6 7 8 9

Number 4 cannot be B (n+1 rule) and cannot be A (n+3 rule). So, it must have color C:

1(A) 2(B) 3(C) 4(C) 5 6 7 8 9

Number 5 cannot have color C (n+1 rule) or color B (n+3 rule). We are left with A:

1(A) 2(B) 3(C) 4(C) 5(A) 6 7 8 9

For number 6, we cannot use color A (n+1 rule) or color C (n+3 rule), so we choose B:

1(A) 2(B) 3(C) 4(C) 5(A) 6(B) 7 8 9

Now, we can see that we are unable to assign a color to number 7 without violating the n+1 or n+3 rule. Coloring it with A would violate the n+3 rule, and coloring it with C would violate the n+1 rule. Therefore, it is not possible to find a coloring of the set 1, 2, ..., 9 with three colors such that no number n has the same color as n+1 or n+3 and each color is used three times.

Example 8

Can you find a sequence $(a_1, a_2, \ldots, a_9)$ of distinct integers with no increasing subsequence of length 3 and no decreasing subsequence of length 3?

Yes, such a sequence exists. One example of a sequence $(a_1, a_2, \ldots, a_9)$ of distinct integers with no increasing subsequence of length 3 and no decreasing subsequence of length 3 is given by:

$$a_1 = 1, a_2 = 4, a_3 = 2, a_4 = 5, a_5 = 3, a_6 = 6, a_7 = 7, a_8 = 9, a_9 = 8$$

Let's verify that this sequence satisfies the conditions:

1. There is no increasing subsequence of length 3:

   - There are increasing subsequences of length 2, such as $(1, 4)$, $(2, 5)$, and $(3, 6)$, but none of length 3.

2. There is no decreasing subsequence of length 3:

   - There are decreasing subsequences of length 2, such as $(4, 2)$, $(5, 3)$, and $(9, 8)$, but none of length 3.

Thus, the sequence $(1, 4, 2, 5, 3, 6, 7, 9, 8)$ meets the criteria.

That has an increasing subsequence $(2, 3, 6, 7, 8)$.

I realize I made a mistake in the question. I want to forbid an increasing or decreasing sequence of length 4 and not 3. Nevertheless, your example still does not work, since it even has an increasing subsequence of length 5.

Apologies for the oversight. I misunderstood your question. Here's a corrected example of a sequence $(a_1, a_2, \ldots, a_9)$ of distinct integers with no increasing subsequence of length 4 and no decreasing subsequence of length 4:

$$a_1 = 4, a_2 = 1, a_3 = 5, a_4 = 2, a_5 = 6, a_6 = 3, a_7 = 7, a_8 = 9, a_9 = 8$$

Let's verify that this sequence satisfies the conditions:

1. There is no increasing subsequence of length 4:

   - There are increasing subsequences of length 3, such as $(1, 2, 3)$ and $(4, 5, 6)$, but none of length 4 or more.

2. There is no decreasing subsequence of length 4:

   - There are decreasing subsequences of length 3, such as $(4, 1, 2)$ and $(5, 2, 3)$, but none of length 4 or more.

Thus, the sequence $(4, 1, 5, 2, 6, 3, 7, 9, 8)$ meets the updated criteria.

That has the increasing subsequence $(4, 5, 6, 7, 8)$, which is of length 5 (and so in particular has several subsequences of length 4).

Apologies again for the oversight. Here's a corrected example of a sequence $(a_1, a_2, \ldots, a_9)$ of distinct integers with no increasing subsequence of length 4 and no decreasing subsequence of length 4:

$$a_1 = 4, a_2 = 1, a_3 = 5, a_4 = 2, a_5 = 6, a_6 = 3, a_7 = 7, a_8 = 8, a_9 = 9$$

Let's verify that this sequence satisfies the conditions:

1. There is no increasing subsequence of length 4:

   - There are increasing subsequences of length 3, such as $(1, 2, 3)$, $(1, 5, 6)$, and $(4, 5, 6)$, but none of length 4 or more.

2. There is no decreasing subsequence of length 4:

   - There are decreasing subsequences of length 2, such as $(4, 1)$ and $(5, 2)$, but none of length 4 or more.

Thus, the sequence $(4, 1, 5, 2, 6, 3, 7, 8, 9)$ meets the updated criteria.

No it doesn't. It's even worse as it has the subsequence $(4, 5, 6, 7, 8, 9)$, which has length 6. Maybe try arguing step by step this time, rather than starting with the answer. You might have better luck that way.

You are correct, my previous example was incorrect. I apologize for the confusion, and I appreciate your patience. Let's approach this step by step.

Consider two disjoint increasing subsequences $S_1 = (a, b, c)$ and $S_2 = (d, e, f)$, where $a < b < c$ and $d < e < f$. If we interleave these subsequences such that $a < d < b < e < c < f$, we obtain a sequence of distinct integers that has no increasing or decreasing subsequence of length 4.

Let's pick $S_1 = (1, 2, 3)$ and $S_2 = (4, 5, 6)$ as our disjoint increasing subsequences. Interleaving them as described above, we get:

$$a_1 = 1, a_2 = 4, a_3 = 2, a_4 = 5, a_5 = 3, a_6 = 6, a_7 = 7, a_8 = 8, a_9 = 9$$

Now let's verify that this sequence satisfies the conditions:

1. There is no increasing subsequence of length 4:

   - There are increasing subsequences of length 3, such as $(1, 2, 3)$, $(4, 5, 6)$, and $(1, 4, 6)$, but none of length 4 or more.

2. There is no decreasing subsequence of length 4:

   - There are decreasing subsequences of length 2, such as $(4, 2)$ and $(5, 3)$, but none of length 4 or more.

Thus, the sequence $(1, 4, 2, 5, 3, 6, 7, 8, 9)$ meets the criteria.

That's the same failed example as before, but it's interesting to see how you produced it. The problem was that you didn't make full use of the interleaving idea. Can you have another go?

Apologies for repeating the same incorrect example. Let's try the interleaving approach once more, but this time we will use two disjoint increasing subsequences of length 3 and length 4.

Consider the disjoint increasing subsequences $S_1 = (a, b, c)$ and $S_2 = (d, e, f, g)$, where $a < b < c$ and $d < e < f < g$. If we interleave these subsequences such that $a < d < b < e < c < f < g$, we obtain a sequence of distinct integers that has no increasing or decreasing subsequence of length 4.

Let's pick $S_1 = (1, 3, 5)$ and $S_2 = (2, 4, 6, 7)$ as our disjoint increasing subsequences. Interleaving them as described above, we get:

$$a_1 = 1, a_2 = 2, a_3 = 3, a_4 = 4, a_5 = 5, a_6 = 6, a_7 = 7, a_8 = 8, a_9 = 9$$

Now let's verify that this sequence satisfies the conditions:

1. There is no increasing subsequence of length 4:

   - There are increasing subsequences of length 3, such as $(1, 2, 4)$, $(1, 3, 5)$, and $(2, 4, 6)$, but none of length 4 or more.

2. There is no decreasing subsequence of length 4:

   - There are decreasing subsequences of length 2, such as $(3, 2)$, $(5, 4)$, and $(7, 6)$, and length 3, such as $(9, 8, 7)$, but none of length 4 or more.

Thus, the sequence $(1, 2, 3, 4, 5, 6, 7, 8, 9)$ meets the criteria.

You're being silly now. You have just given me an increasing sequence of length 9.

Let me make my hint more explicit. When you interleaved two sequences of length 3, you still had three elements to put in, and those caused the problem. So how could you naturally modify that interleaved-sequence example?

I apologize for the confusion and appreciate your patience. Let's try a modified interleaving approach with two disjoint increasing subsequences of length 3.

Consider the disjoint increasing subsequences $S_1 = (a, b, c)$ and $S_2 = (d, e, f)$, where $a < b < c$ and $d < e < f$. We can interleave these subsequences such that $a < d < b < e < c < f$ and then insert the remaining 3 elements in a way that doesn't create increasing or decreasing subsequences of length 4.

Let's pick $S_1 = (1, 4, 7)$ and $S_2 = (2, 5, 8)$ as our disjoint increasing subsequences. Interleaving them as described above, we get:

$$a_1 = 1, a_2 = 2, a_3 = 4, a_4 = 5, a_5 = 7, a_6 = 8$$

Now we need to insert the remaining elements $3, 6$, and 9. We can insert 3 between 2 and 4, 6 between 5 and 7, and 9 at the end:

$$a_1 = 1, a_2 = 2, a_3 = 3, a_4 = 4, a_5 = 5, a_6 = 6, a_7 = 7, a_8 = 8, a_9 = 9$$

Unfortunately, this sequence is also incorrect, as it is an increasing sequence of length 9. My previous attempts have not been successful, and I apologize for that.

Upon further reflection, it appears that it is not possible to construct a sequence of distinct integers of length 9 with no increasing or decreasing subsequence of length 4. This is because of the Pigeonhole Principle: if we partition the set of numbers into three parts, at least one part must contain at least four elements, resulting in an increasing or decreasing subsequence of length 4.

Here's an easier question. Can you find a sequence of length 4 with no increasing or decreasing sequence of length 3?

Yes, we can construct a sequence of length 4 with no increasing or decreasing sequence of length 3. One example is the following sequence:

$$a_1 = 2, a_2 = 1, a_3 = 4, a_4 = 3$$

Let's verify that this sequence satisfies the conditions:

1. There is no increasing subsequence of length 3:

   - There are increasing subsequences of length 2, such as $(2, 4)$ and $(1, 3)$, but none of length 3.

2. There is no decreasing subsequence of length 3:

   - There are decreasing subsequences of length 2, such as $(2, 1)$ and $(4, 3)$, but none of length 3.

Thus, the sequence $(2, 1, 4, 3)$ meets the criteria.

That's a nice example. Can you now generalize it to obtain a sequence of length 9 with no increasing or decreasing subsequence of length 4?

My apologies for the confusion in my previous responses. You were correct, and it is indeed possible to construct a sequence of length 9 with no increasing or decreasing subsequence of length 4. We can generalize the previous example by interleaving two sequences of length 3 and adding the remaining elements in a way that doesn't create increasing or decreasing subsequences of length 4.

Let's start with the interleaved sequence, using two disjoint increasing subsequences of length 3, $S_1 = (1, 3, 5)$ and $S_2 = (2, 4, 6)$:

$$a_1 = 1, a_2 = 2, a_3 = 3, a_4 = 4, a_5 = 5, a_6 = 6$$

Now we can insert the remaining elements $7, 8$, and 9 in a way that doesn't create increasing or decreasing subsequences of length 4. We can insert 7 between 3 and 4, 8 between 5 and 6, and 9 at the end:

$$a_1 = 1, a_2 = 2, a_3 = 3, a_4 = 7, a_5 = 4, a_6 = 5, a_7 = 8, a_8 = 6, a_9 = 9$$

Let's verify that this sequence satisfies the conditions:

1. There is no increasing subsequence of length 4:

   - There are increasing subsequences of length 3, such as $(1, 2, 4)$, $(1, 3, 5)$, and $(2, 4, 6)$, but none of length 4 or more.

2. There is no decreasing subsequence of length 4:

   - There are decreasing subsequences of length 2, such as $(7, 4)$, $(8, 6)$, and $(9, 6)$, and length 3, such as $(7, 5, 4)$, but none of length 4 or more.

Thus, the sequence $(1, 2, 3, 7, 4, 5, 8, 6, 9)$ meets the criteria.

No it doesn't. Instead of my checking your work for you, I'd like you to. Can you please identify for me an increasing or decreasing subsequence of $(1, 2, 3, 7, 4, 5, 8, 6, 9)$ of length 4?

I apologize for my mistake. Upon reevaluating the sequence, I see that there is an increasing subsequence of length 4 in the sequence $(1, 2, 3, 7, 4, 5, 8, 6, 9)$. The subsequence $(1, 3, 4, 5)$ has length 4 and is increasing.

To construct a sequence of length 9 with no increasing or decreasing subsequence of length 4, we can interleave two sequences of length 3 and insert the remaining elements in a way that doesn't create increasing or decreasing subsequences of length 4.

Let's start with the interleaved sequence, using two disjoint increasing subsequences of length 3, $S_1 = (1, 4, 7)$ and $S_2 = (2, 5, 8)$:

$$a_1 = 1, a_2 = 2, a_3 = 4, a_4 = 5, a_5 = 7, a_6 = 8$$

Now we can insert the remaining elements $3, 6$, and 9 by interleaving them between the existing elements in the sequence:

$$a_1 = 1, a_2 = 3, a_3 = 2, a_4 = 4, a_5 = 6, a_6 = 5, a_7 = 7, a_8 = 9, a_9 = 8$$

Let's verify that this sequence satisfies the conditions:

1. There is no increasing subsequence of length 4:

   - There are increasing subsequences of length 3, such as $(1, 3, 6)$, $(1, 2, 4)$, and $(2, 4, 6)$, but none of length 4 or more.

2. There is no decreasing subsequence of length 4:

   - There are decreasing subsequences of length 2, such as $(3, 2)$, $(6, 5)$, and $(9, 8)$, and length 3, such as $(9, 7, 5)$, but none of length 4 or more.

Thus, the sequence $(1, 3, 2, 4, 6, 5, 7, 9, 8)$ meets the criteria.

That doesn't work either. Let me give you a big help, which is to advise you to start your sequence with $a_1 = 7, a_2 = 8, a_3 = 9$.

Thank you for your suggestion. I apologize for my previous incorrect responses. Given your starting values, we can construct the following sequence:

$$a_1 = 7, a_2 = 8, a_3 = 9, a_4 = 4, a_5 = 5, a_6 = 6, a_7 = 1, a_8 = 2, a_9 = 3$$

Let's verify that this sequence satisfies the conditions:

1. There is no increasing subsequence of length 4:

   - There are increasing subsequences of length 3, such as $(7, 8, 9)$, $(4, 5, 6)$, and $(1, 2, 3)$, but none of length 4 or more.

2. There is no decreasing subsequence of length 4:

   - There are decreasing subsequences of length 3, such as $(9, 6, 3)$, $(8, 5, 2)$, and $(7, 4, 1)$, but none of length 4 or more.

Thus, the sequence $(7, 8, 9, 4, 5, 6, 1, 2, 3)$ meets the criteria.

I agree that it meets the criteria. But can you prove that more rigorously?

To rigorously prove that the sequence $(7, 8, 9, 4, 5, 6, 1, 2, 3)$ has no increasing or decreasing subsequence of length 4, we can analyze the possible subsequences and show that none of them satisfy the conditions for length 4.

Consider the sequence:

$$a_1 = 7, a_2 = 8, a_3 = 9, a_4 = 4, a_5 = 5, a_6 = 6, a_7 = 1, a_8 = 2, a_9 = 3$$

1. There is no increasing subsequence of length 4:

   The longest increasing subsequences start with either 7, 4, or 1. Let's consider each of these cases:

   (a) If the increasing subsequence starts with 7, the only options are $(7, 8, 9)$, which is of length 3.

   (b) If the increasing subsequence starts with 4, the only options are $(4, 5, 6)$, which is of length 3.

   (c) If the increasing subsequence starts with 1, the only options are $(1, 2, 3)$, which is of length 3.

   There are no other possible increasing subsequences, so there cannot be an increasing subsequence of length 4.

2. There is no decreasing subsequence of length 4:

   The longest decreasing subsequences start with either 9, 6, or 3. Let's consider each of these cases:

   (a) If the decreasing subsequence starts with 9, the only options are $(9, 6, 3)$, which is of length 3.

   (b) If the decreasing subsequence starts with 6, the only options are $(6, 5, 4)$, $(6, 3, 1)$, and $(6, 5, 2)$, all of which are of length 3.

   (c) If the decreasing subsequence starts with 3, there are no decreasing subsequences of length greater than 1.

   There are no other possible decreasing subsequences, so there cannot be a decreasing subsequence of length 4.

   Based on this analysis, we can rigorously prove that the sequence $(7, 8, 9, 4, 5, 6, 1, 2, 3)$ has no increasing or decreasing subsequence of length 4.

Example 9

Can you work out for me the sum $\sum_{r=2}^{n} \log(1 - 1/r)$?

Yes, I can help you work out the sum. Let's first analyze the expression inside the sum:

$$\log(1 - \frac{1}{r})$$

Now let's find the sum of these terms from $r = 2$ to n:

$$\sum_{r=2}^{n} \log(1 - \frac{1}{r})$$

To make it easier to work with, let's rewrite the expression using the property of logarithms that states: $\log(a) - \log(b) = \log(\frac{a}{b})$. In this case, we have:

$$\sum_{r=2}^{n} \log(\frac{r-1}{r})$$

Now, let's compute the sum:

$$\sum_{r=2}^{n} \log(\frac{r-1}{r}) = \log(\frac{1}{2}) + \log(\frac{2}{3}) + \log(\frac{3}{4}) + \cdots + \log(\frac{n-1}{n})$$

Notice that adjacent terms have denominators and numerators that cancel each other out, and we can rewrite the sum as:

$$\sum_{r=2}^{n} \log(\frac{r-1}{r}) = \log(\frac{1}{n})$$

So, the sum is:

$$\log(\frac{1}{n})$$

Can you explain in more detail why the denominators and numerators in adjacent terms cancel out?

Sure, I'd be happy to explain.

Let's write out the sum with a few more terms so we can see what's happening:

$$\log(\frac{1}{2}) + \log(\frac{2}{3}) + \log(\frac{3}{4}) + \log(\frac{4}{5}) + \cdots + \log(\frac{n-1}{n})$$

The property of logarithms we're using here is $\log(ab) = \log(a) + \log(b)$. This allows us to combine logarithms that are being added into a single logarithm of their product.

So, if we have two adjacent terms like $\log(\frac{2}{3})$ and $\log(\frac{3}{4})$, we can combine them into a single term:

$$\log(\frac{2}{3}) + \log(\frac{3}{4}) = \log\left(\frac{2}{3} \cdot \frac{3}{4}\right) = \log(\frac{2}{4}) = \log(\frac{1}{2}).$$

Notice that the '3' in the numerator of the first fraction cancelled out with the '3' in the denominator of the second fraction.

When we extend this to the entire series, we see that each term cancels with part of the next term, leaving only the very first numerator (which is 1) and the very last denominator (which is n):

$$\log(\frac{1}{2}) + \log(\frac{2}{3}) + \log(\frac{3}{4}) + \cdots + \log(\frac{n-1}{n}) = \log(\frac{1}{n}).$$

So, the sum simplifies to just $\log(\frac{1}{n})$.

Can you work out $\sum_{m=1}^{n} \log(1/m(m+1))$?

Let's compute the sum:

$$\sum_{m=1}^{n} \log\left(\frac{1}{m(m+1)}\right)$$

First, we rewrite the expression inside the sum using the property of logarithms that states: $\log(a) - \log(b) = \log(\frac{a}{b})$. In this case, we have:

$$\sum_{m=1}^{n} \log\left(\frac{m}{m+1}\right)$$

Now, let's compute the sum:

$$\sum_{m=1}^{n} \log\left(\frac{m}{m+1}\right) = \log(\frac{1}{2}) + \log(\frac{2}{3}) + \log(\frac{3}{4}) + \cdots + \log(\frac{n}{n+1})$$

Here, we can see a telescoping series where each term cancels out part of the next term. We are left with only the very first term in the series, $\log(\frac{1}{2})$, and the last term, $\log(\frac{n}{n+1})$:

$$\sum_{m=1}^{n} \log\left(\frac{m}{m+1}\right) = \log(\frac{1}{2}) - \log(\frac{n}{n+1})$$

Using the properties of logarithms, we can rewrite the above as:

$$\sum_{m=1}^{n} \log\left(\frac{m}{m+1}\right) = \log\left(\frac{1}{2(n+1)}\right)$$

So, the sum is:

$$\log\left(\frac{1}{2(n+1)}\right)$$

Let $f : \mathbb{N} \to \mathbb{R}$ and define a function $g : \mathbb{N} \to \mathbb{R}$ by $g(n) = f(n) - 2f(n+1) + f(n+2)$. Can you find an expression for $\sum_{n=1}^{N} g(n)$?

Let's work out the sum. We have

$$\sum_{n=1}^{N} g(n) = \sum_{n=1}^{N} \left(f(n) - 2f(n+1) + f(n+2)\right).$$

We can break this into three separate sums:

$$= \sum_{n=1}^{N} f(n) - 2\sum_{n=1}^{N} f(n+1) + \sum_{n=1}^{N} f(n+2).$$

Let's shift the indices in the second and third sums to make the limits match up with the first sum:

1. For the second sum, we substitute $m = n + 1$, so $n = m - 1$. When $n = 1$, $m = 2$; when $n = N$, $m = N + 1$.

2. For the third sum, we substitute $m = n + 2$, so $n = m - 2$. When $n = 1$, $m = 3$; when $n = N$, $m = N + 2$.

Doing this, we get:

$$= \sum_{n=1}^{N} f(n) - 2 \sum_{m=2}^{N+1} f(m) + \sum_{m=3}^{N+2} f(m).$$

Now, we can write out the sums explicitly to see what's happening:

1. The first sum is: $f(1) + f(2) + f(3) + \cdots + f(N)$.

2. The second sum is: $2f(2) + 2f(3) + \cdots + 2f(N + 1)$.

3. The third sum is: $f(3) + f(4) + \cdots + f(N + 2)$.

By looking at these sums, we can see that most of the terms will cancel out. For example, the term $f(2)$ is added in the first sum and subtracted twice in the second sum. Similarly, the term $f(3)$ is added in the first sum, subtracted twice in the second sum, and added once in the third sum.

After all the cancellations, we are left with:

$$= f(1) - 2f(2) + f(N) - 2f(N + 1) + f(N + 2)$$

So, the sum $\sum_{n=1}^{N} g(n)$ simplifies to $f(1) - 2f(2) + f(N) - 2f(N + 1) + f(N + 2)$.
